# The Synthesis of Picolinamide-Supported Tetracoordinated Organoboron Complexes with Aggregation-Induced Emission Property

**DOI:** 10.3389/fchem.2022.856832

**Published:** 2022-03-22

**Authors:** Gaoqiang You, Liang Xu, Yu Wei

**Affiliations:** Key Laboratory for Green Processing of Chemical Engineering of Xin-Jiang Bingtuan, School of Chemistry and Chemical Engineering, Shihezi University, Shihezi, China

**Keywords:** tetracoordinated organoboron, AIE, picolinamide, potassium trifluoroborate, manganese

## Abstract

The picolinamide-supported tetracoordinated organoboron complexes containing diaryl boronyl segments have been synthesized for the first time. Aryl trifluoroborates were utilized as the BAr_2_ sources to introduce different aryl motifs with diverse functional groups. The optical experiments discovered these five-membered boron-containing complexes were aggregation-induced emission (AIE) active, thus affording a new class of AIE molecules.

## Introduction

Taking advantage of the vacant *p*-orbitals of the boron atoms, the chelating complexation between π-conjugated ligands and -BR_2_ units will generate tetracoordinated organoboron complexes containing X→B dative bonds that are able to lock the π-conjugated ligands. These modifications will enhance the molecular rigidity, extend the π-conjugation system, and thus afford molecules with superior photoluminescence properties (Chen et al., 2017; Dou et al., 2017; Mellerup and Wang, 2019; Yan et al., 2019; Huang et al., 2020). Therefore, over the past decades, the synthesis, improvement, and application of these tetracoordinated organoboron complexes have attracted a continuing interest from organic and materials communities. However, the emission of these planar structures in aggregates or the solid-state is usually spoiled due to the aggregation-caused quenching (ACQ) effects. This limits their applications in organic optoelectronic materials or other situations that demand solid-state emissions, such as organic lasers and organic light-emitting diodes (OLEDs), which necessitates the development of new series of these complexes with high solid-state luminescence efficiency.

**FIGURE 1:**
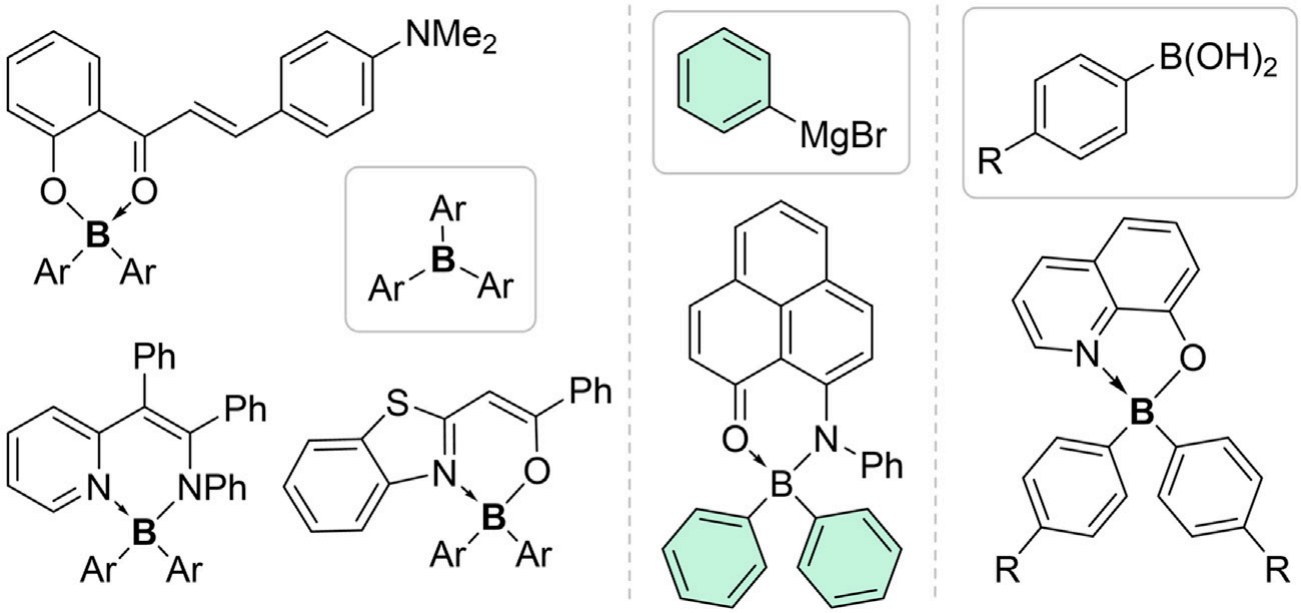
The tetracoordinated organoboron complexes containing BAr_2_ segments with AIE properties. The aryl precursors are indicated in the boxes.

Since the conceptualization of the aggregation-induced emission (AIE) effect by the Tang group in 2001 (Luo et al., 2001; Mei et al., 2015), many types of AIE-active compounds/units have been developed. In recent years, a number of AIE-active tetracoordinated organoboron complexes have been synthesized and applied as aggregate-state emitters (Virgili et al., 2013; Liu et al., 2019; Nitti et al., 2020; Ito et al., 2021; Yin et al., 2021; Min et al., 2022). These AIE-active complexes, which are usually obtained *via* linking AIE-active units such as tetraphenylethylene (TPE) or introducing rotational aryls onto the chelating backbones, mostly contain BF_2_ segments. The introduction of BAr_2_ segments, which had two bulky aryl rings on boron atoms, has been utilized to disrupt the intermolecular π−π stacking in aggregate states and thus enable more efficient AIE effects (Figure 1). Based on our survey, most of the known AIE-active complexes with BAr_2_ segments were six-membered. To the best of our knowledge, the only AIE-active five-membered cases have been disclosed very recently by Yang and Zou group (Qi et al., 2021). This was surprising since five-membered tetracoordinated organoboron compounds were very commonly encountered in organoboron-based molecules and materials. Moreover, BAr_2_ segments were usually introduced via Ar_3_B or aryl organometallic reagents (Kubota et al., 2012; Yan et al., 2014; Gong et al., 2015; Wang et al., 2015; Wu et al., 2015), limiting the accessibility and diversity of the target molecules (Yang et al., 2021).

On the other hand, innumerable chelating backbones have been applied in the complexation formation of tetracoordinated organoboron, to fine-tune the desirable luminescence properties. In this regard, it is surprising that picolinamide, one of the most available and widely-used chelating ligands in coordination chemistry, has never been used in the preparation of tetracoordinated organoboron, to the best of our knowledge (Yang et al., 2021). Thus, we hypothesized that complexation between picolinamide and BAr_2_ segments would afford a new series of tetracoordinated organoboron molecules. These complexes should adopt propeller structures and be consisted of electron-deficient pyridyl, electron-rich anilino groups, and the spiro linker centers of boron atoms, which potentially circumvent the trade-off between the complexation stability and the solid-state luminescence, leading to some special light-emitting molecules (Shiu et al., 2016). The proposal would pose at least two major challenges: 1) to properly introduce the relatively flexible picolinamide ligands and 2) to bypass the traditional organometallic BAr_2_ incorporation pathways.

## Results and Discussion

With these considerations in mind, the investigation was initiated by exploring the reaction between *N*-phenylpyridinecarboxamide **1a** and potassium phenyltrifluoroborate **2a** (Sawazaki et al., 2018; Wang et al., 2019), which has been demonstrated to be a competent BPh_2_ provider recently by Song group (Yang et al., 2018) and our group (Zu et al., 2020) (Table 1). After systematic evaluation of reaction variables, to our delight, under similar reaction conditions with our previous work (Zu et al., 2020), the target product **3a** could be isolated in 99% yield by using Mn (1.0 equiv), *p*-toluenesulfonyl chloride (TsCl, 2.5 equiv) and Na_2_CO_3_ (0.5 equiv) in acetonitrile (CH_3_CN) at 130°C for 24 h (entry 1). The propeller structure of **3a** was confirmed unambiguously *via* X-ray diffraction analysis of its crystals (Table 2).

**TABLE 1:** Optimization of reaction conditions^a^.

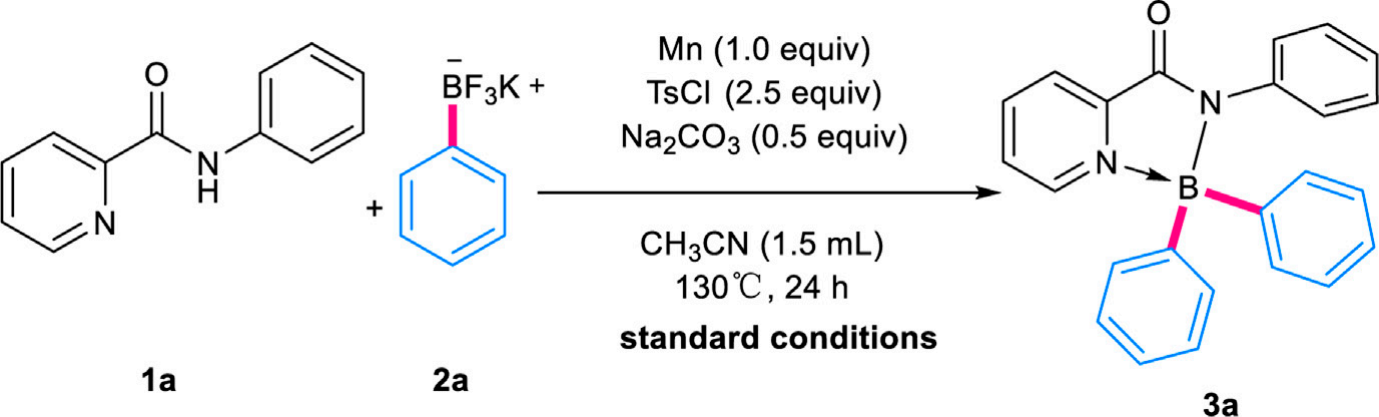

Entry	Variations from the standard conditions	Yields (%)^b^
1	None	99
2	Without Mn	81
3	Without TsCl	N.R.
4	Without Na_2_CO_3_	24
5	DMF instead of MeCN	Trace
6	DMA instead of MeCN	Trace
7	THF instead of MeCN	Trace
8	NMP instead of MeCN	Trace
9	DMSO instead of MeCN	Trace
10	HFIP instead of MeCN	Trace
11	90°C instead of 130°C	N.R.
12	110°C instead of 130°C	66
13	K_3_PO_4_ instead of Na_2_CO_3_	24
14	Cs_2_CO_3_ instead of Na_2_CO_3_	27
15	LiOH instead of Na_2_CO_3_	41
16	PhBF_3_K (4.0 equiv)	41
17	PhBF_3_K (3.0 equiv)	23

a Reaction conditions: **1a** (0.15 mmol), **2a** (0.75 mmol, 5 equiv), air, CH_3_CN (1.5 ml), 130°C, 24 h.

b Isolated yields.

**TABLE 2:** Optimization of reaction conditions^a^.

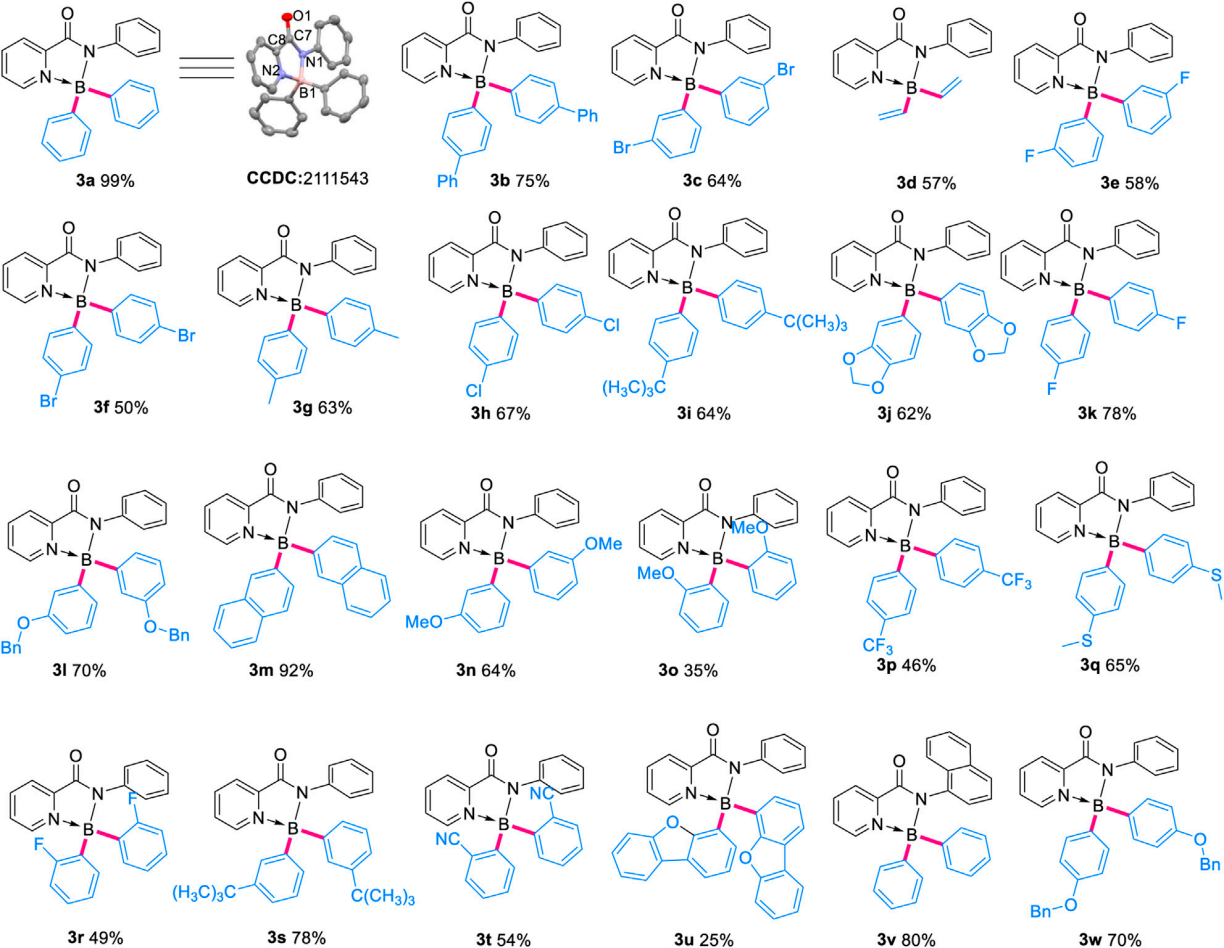

a Reaction conditions: pyridinecarboxamide **1** (0.15 mmol), potassium aryl trifluoroborate **2** (0.75 mmol, 5 equiv), air, CH_3_CN (1.5 ml), 130°C, 24 h.

Subsequently, control experiments were performed to elucidate the function of Mn, TsCl and Na_2_CO_3_ in these conditions (entry 2–4). It was found TsCl played a vital role in the formation of **3a**, in view that its absence would suppress the reaction totally. Omitting Mn from the conditions, still, 81% isolated yield of **3a** could be obtained, while the omission of Na_2_CO_3_ led to the formation of **3a** in only 24% yield. Further evaluation revealed the choice of solvent was also critical (entry 5–10). Other commonly-used organic solvents, including DMF, THF and DMSO, afforded only a trace amount of product **3a**. Moreover, the test of the reaction temperature showed the yield dropped sharply while lowering the temperature (entries 11–12). The investigation of a variety of bases showed that Na_2_CO_3_ performed best (entries 13–15). The reduction of the amount of **2a** also affected the yields remarkably (entries 16–17).

With the optimal conditions in hand, the substrate scope of this methodology was then investigated (Table 2). A broad scope of aryl trifluoroborates containing *para*- *ortho*- and *meta*-substituents were applied in the reactions successfully. Generally, the *ortho*-substituted aryl trifluoroborates gave lower isolated yields, in comparison with their *para*- and *meta*-substituted analogs, mainly due to the steric effect (**3e**, 58% vs. **3k**, 78% vs. **3r**, 49%; **3n**, 64% vs. **3o**, 35%). The reaction tolerated bromides and chlorides well (**3c**, **3f, 3h**), allowing the formation of halogenated products and thus leaving ample space for further decorations. No matter electron-withdrawing (-CF_3_, -halides, -CN) or -donating (-OMe, -^*t*^Bu, -OBn) groups were on the aryl trifluoroborates, the target boronyl complexes could be obtained in moderate to good yields. While aryl substrates containing π-extended systems were applied, products with more congested BAr_2_ segments, like **3b**, **3m** and **3u**, were obtained. Notably, potassium vinyl fluoroborate proceeded smoothly to provide **3d** in moderate yields. When the *N*-substituent of pyridinecarboxamide was changed to 1-naphthyl, 80% of **3v** could be isolated.

**FIGURE 2:**
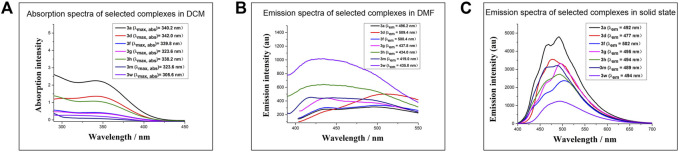
The absorption and emission spectra of selected complexes.

Then, photophysical properties of the obtained complexes in solvent and in the solid-state were measured. As shown in Figure 2 and Table 3, the selected complexes possessed very weak luminescence in organic solvent DMF along with very low quantum yield (Φ_liquid_ < 0.015), probably due to the non-radiative process in the solvent induced by intramolecular rotation. In contrast, the solid-state spectra of the BAr_2_-species showed intense emission bands centered around 490 nm with high quantum yield (Φ_solid_: 31–98%), indicating the existence of AIE phenomenon and the absence of strong intermolecular π-π interactions in the solid-state.

**TABLE 3:** Luminescence quantum yield and lifetime of selected complexes.

	3a	3d	3f	3g	3h	3m	3w
Ф_liquid_ (%)^a^	0.31	0.39	0.39	0.64	0.59	1.02	1.09
τ_liquid_ (ns)^a^	1.99	1.86	1.91	1.76	2.03	1.86	2.01
Ф_solid_ (%)	78.38	82.46	44.26	89.54	97.83	82.96	31.48
τ_solid_ (ns)	3.64	4.29	3.01	3.84	3.31	2.98	3.29

a Sample concentration 10^–4^ M.

**FIGURE 3:**
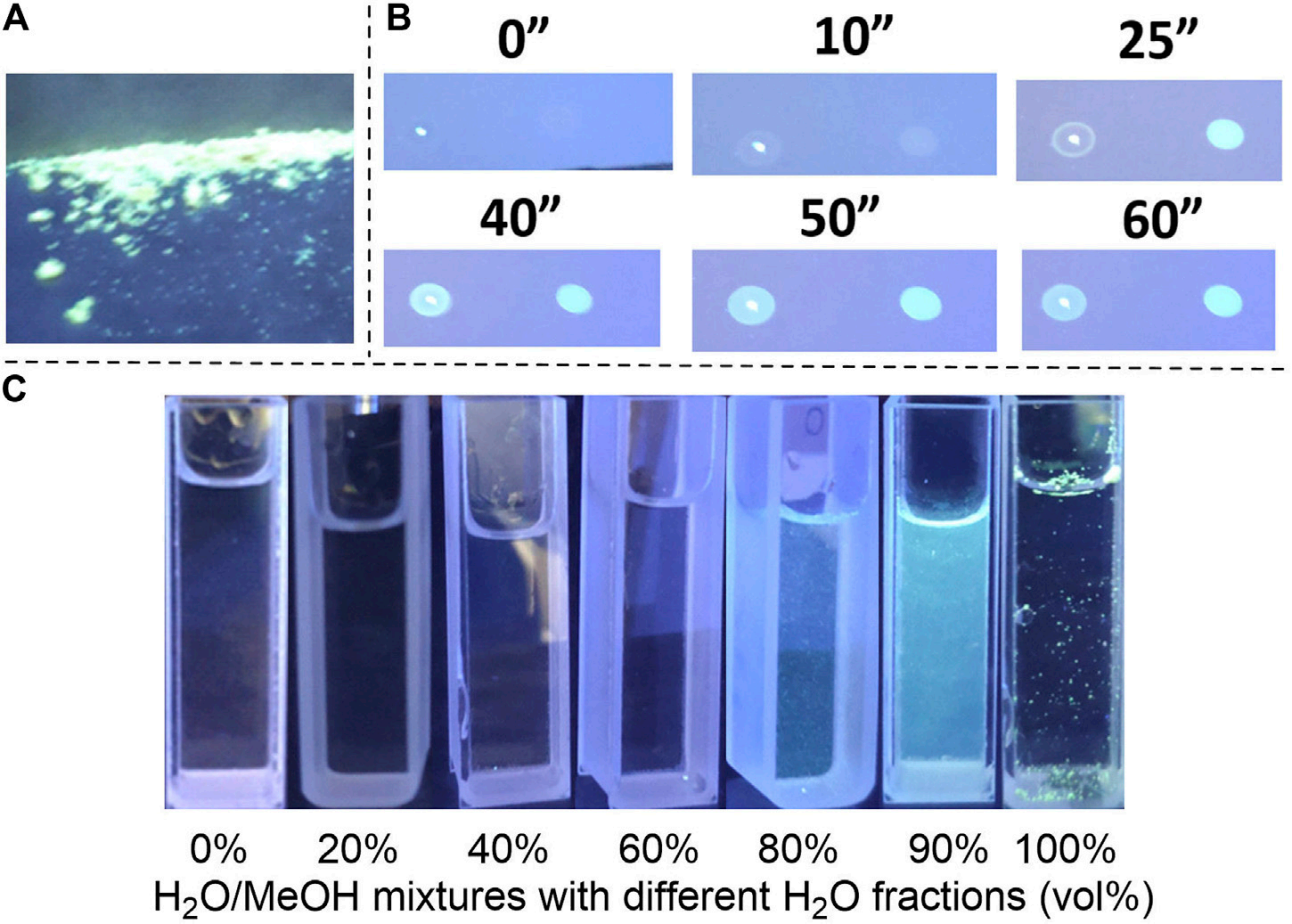
AIE behaviours of **3a**. **(A) 3a** solid at 365 nm. **(B)** Fluorescence comparison of **3a** solution at 365 nm at different time. **(C)** Fluorescence comparison of **3a** at 365 nm in different ratios of water/methanol.

Furthermore, the emission properties of **3a** were investigated in detail to prove its AIE behaviors (Figure 3). Firstly, under irradiation with a UV lamp at 365 nm, solid **3a** showed macroscopical fluorescence (Figure 3A). Then, **3a** was dissolved in H_2_O/MeOH mixtures with different H_2_O content (Figure 3C). When the H_2_O content is lower than 60%, **3a** was dissolved totally and the solution was transparent without observable fluorescence. As the water content increased from 80 to 90%, **3a** became insoluble in the solvents and began to aggregate. Correspondingly, the turbidity and luminescence (at 365 nm) of the solution increased, which could be observed with the naked eyes. **3a** would disperse in the pure water with its aggregation showing the same luminescence as the solid-state. The AIE behavior of **3a** was further verified by observing the fluorescent changes during the evaporation of MeOH and THF solution of **3a** (Figure 3B). On a thin-layer chromatography plate, as the solvent evaporated and **3a** molecules aggregated, a clear increase in the fluorescence was observed under UV irradiation at 365 nm.

**FIGURE 4:**
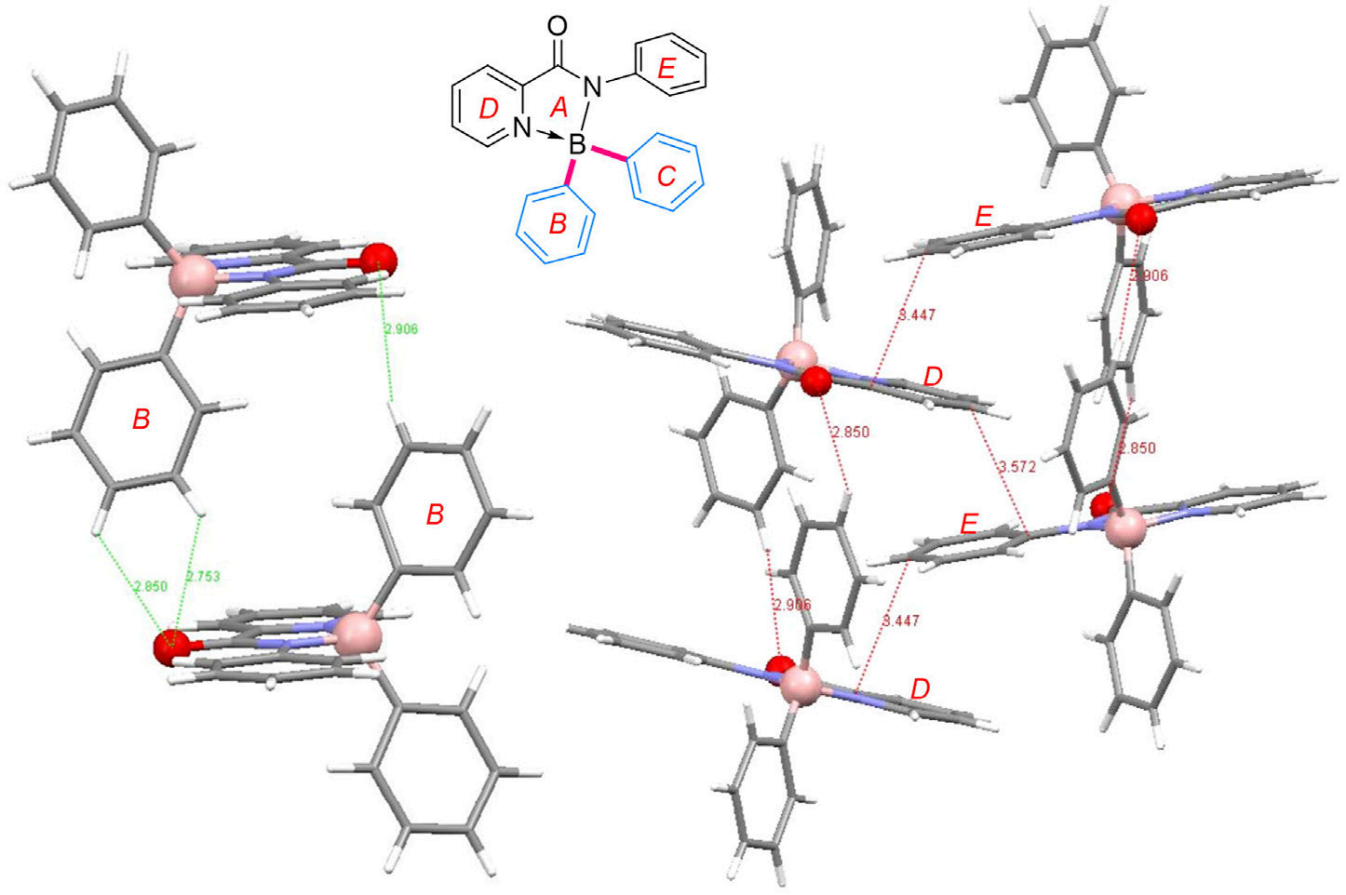
The crystal structure of **3a**.

In order to better understand the AIE properties of these complexes, X–ray crystallographic analysis of **3a** was performed. As shown in Figure 4, the boron atoms of **3a** adopted a typical tetrahedral geometry to form *N,N*–chelated five-membered rings. The pyridyl N→B bond lengths are around 0.03 Å longer than the amide N–B bonds. As anticipated, **3a** adopt twisted conformations and the two phenyl rings on the boron atom were not coplanar. The dihedral angles of A and B, A and C, B and C were 76.50°, 78.02° and 61.95°, respectively. As for the packing mode, to take advantage of the capacious space around the planar picolinamide motif, two **3a** molecules could assemble in the head-to-tail mode. The aryl hydrogens on the B rings could interact with the carbonyl oxygens of the other molecule to form H-bonding (O1 … H1, 2.85 Å; O1 … H2, 2.75 Å; O2 … H3, 2.91 Å). The aforementioned dimers would interlace zigzag to form weak intermolecular π–π interactions. The shortest distances between the aryl D and E rings were around 3.5 Å. These weak intermolecular interactions, like H-bonding and π–π stacking, were able to fix the molecular conformations in the solid-state, thus inhibiting the internal rotations and non-radiative relaxation and inducing the AIE property.

**FIGURE 5:**
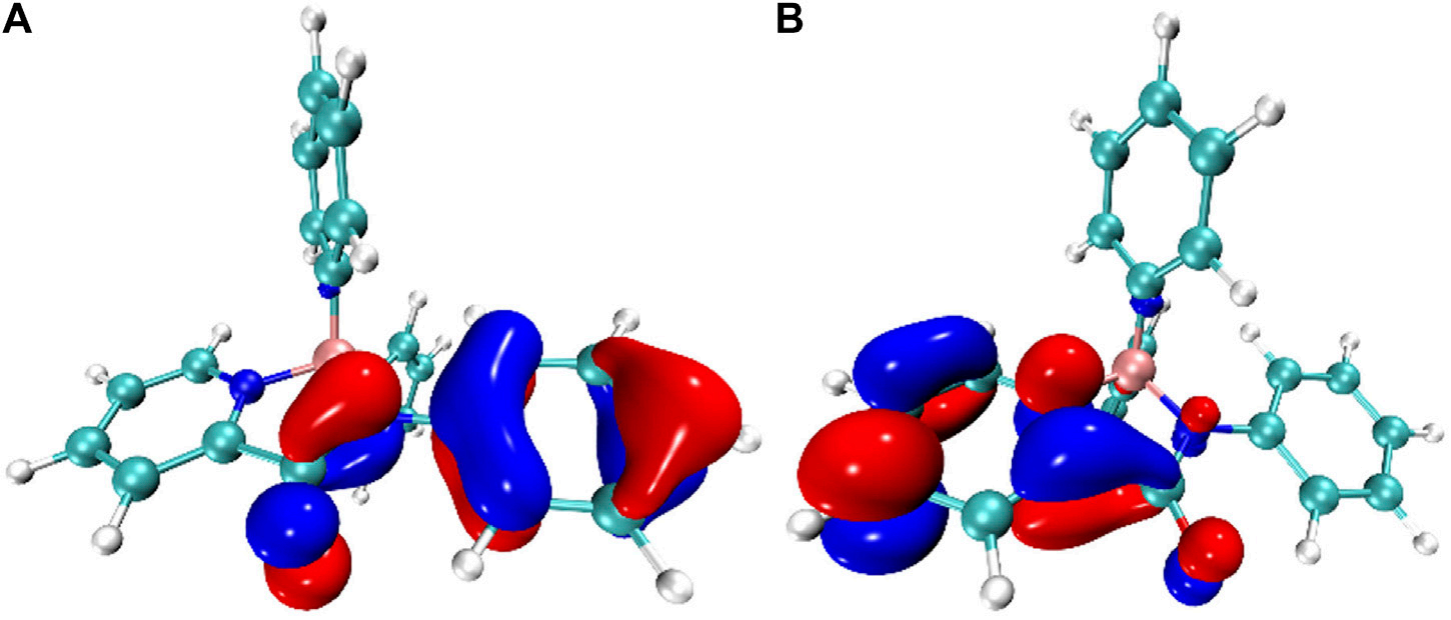
The calculated HOMO **(A)** and LUMO **(B)** orbitals of **3a**.

To gain additional insight, density functional theory (DFT) and time-dependent density functional theory calculations (TD-DFT) (Adamo and Jacquemin, 2013) of **3a** were performed with Gaussian09 suite of programs (Frisch et al., 2009). The combination of m062x/6–311 g** was applied in both cases (Zhao and Truhlar, 2008). As shown in Figure 5, both the HOMO and LUMO orbitals were delocalized over the picolinamide backbone. However, the electron density distribution of HOMO and LUMO was mainly localized at acyl aniline motif and pyridyl group, respectively. The electron-density contribution of boron in both orbitals were negligible, being 0.80% (HOMO) and 0.48% (LUMO). The HOMO and LUMO orbitals were spatially separated by the boron atom, rendering limited overlap between these two orbitals. Then, TD-DFT calculations on the DFT-optimized structure suggested that lowest energy transition (S0→S1) involves mostly the HOMO and the LUMO orbitals, indicating a possible ligand-to-ligand charge transfer character.

In conclusion, using readily available and stable aryl trifluoroborates as the BAr_2_ sources, the picolinamide-based diaryl boronyl complexes have been synthesized for the first time. These propeller-type five-membered complexes exhibit very weak fluorescence in organic solvents and intense fluorescence in their aggregation/solid-state, showing classical AIE properties, which should originate from the confinement of molecular conformations and the inhibited internal rotations when these molecules assemble.

## Experimental Section

### General Information

Unless otherwise noted, all reactions were carried out under an air atmosphere. Analytical thin-layer chromatography (TLC) was performed on glass plates coated with 0.25 mm 230–400 mesh silica gel containing a fluorescent indicator. Visualization was accomplished by exposure to a UV lamp. All the products in this article are compatible with standard silica gel chromatography. Column chromatography was performed on silica gel (200–300 mesh). Eluent generally contained ethyl acetate (EA), petroleum ether (PE) and triethylamine (TEA).

NMR spectra were measured on a Bruker Ascend 400 spectrometer and chemical shifts (δ) are reported in parts per million (ppm). ^1^H NMR spectra were recorded at 400 MHz in NMR solvents and referenced internally to corresponding solvent resonance, and ^13^C NMR spectra were recorded at 101 MHz and referenced to corresponding solvent resonance. Coupling constants are reported in Hz with multiplicities denoted as s (singlet), d (doublet), t (triplet), q (quartet), m (multiplet) and br (broad). Infrared spectra were collected on a Thermo Fisher Nicolet 6700 FT-IR spectrometer using ATR (Attenuated Total Reflectance) method. Absorption maxima (ν max) are reported in wavenumbers (cm^−1^). High resolution mass spectra (HRMS) were acquired on Thermo Scientific LTQ Orbitrap XL with an ESI source. Melting points were measured with a micro-melting point apparatus.

Commercial reagents, including picolinoyl chloride hydrochloride, aniline, naphthalen-1-amine, Mn, *p*-toluenesulfonyl chloride, Na_2_CO_3_ and All potassium trifluoroborate, were purchased from commercial sources and used as received unless otherwise stated.

### Preparation of 2-Pyridinecarboxamide

*N-phenylpicolinamide* ***(1a)*** *.* The target product was prepared according to a literature procedure (Li et al., 2014). The product was isolated by flash chromatography as a white solid (1.64 g, 90%): ^1^H NMR (400 MHz, CDCl_3_) δ 10.03 (s, 1H), 8.62 (d, *J* = 4.4 Hz, 1H), 8.31 (d, *J* = 7.6 Hz, 1H), 7.96–7.87 (m, 1H), 7.82–7.76 (m, 2H), 7.51–7.45 (m, 1H), 7.43–7.36 (m, 2H), 7.15 (t, *J* = 7.2 Hz, 1H). ^13^C{^1^H} NMR (101 MHz, CDCl_3_) δ 162.0, 149.9, 148.0, 137.8, 137.7, 129.1, 126.5, 124.3, 122.4, 119.7, 77.4, 77.1, 76.7.

*N-*(naphthalen-1-yl)picolinamide ***(1v)*** *.* The target product was prepared according to a literature procedure.^14^ The product was isolated by flash chromatography as a white solid (2.03 g, 89%): ^1^H NMR (400 MHz, CDCl_3_) δ 10.77 (s, 1H), 8.75–8.70 (m, 1H), 8.46–8.34 (m, 2H), 8.11 (d, *J* = 8.4 Hz, 1H), 7.98–7.89 (m, 2H), 7.71 (d, *J* = 8.4 Hz, 1H), 7.63–7.50 (m, 4H). ^13^C{^1^H} NMR (101 MHz, CDCl_3_) δ 162.3, 150.1, 148.2, 137.8, 134.1, 132.4, 128.9, 126.6, 126.4, 126.3, 126.3, 126.0, 126.0, 125.9, 125.1, 122.5, 120.8, 120.5, 119.0, 118.6, 109.7, 77.4, 77.1, 76.7.

### Typical Experimental Procedures

#### General Procedure A

A flame-dried 25 ml vial was placed with a magnetic stir bar. Then, *N*-phenylpicolinamide (29.7 mg, 0.15 mmol, 1.0 equiv), potassium trifluoro(phenyl)borate (138.0 mg, 0.75 mmol, 5.0 equiv), Mn (82.0 mg, 0.15 mmol, 1.0 equiv), *p*-toluenesulfonyl chloride (71.5 mg, 0.375 mmol, 2.5 equiv) and Na_2_CO_3_ (79.5 mg, 0.075 mmol, 0.50 equiv) as a base, and react with acetonitrile as the reaction solvent at 130°C for 24 h. After the completion of the reaction and concentration, the crude product was purified by column chromatography (silica gel) to give the target product, using PE/EtOAc/DCM the eluent.

Preparation and characterization data for isolated products:

Compounds **3a**—**3w** are unknown compounds. ^1^H/^13^C NMR, melting point, IR, data HRMS (ESI) m/z for these compounds are provided herein.

*1,1,2-triphenyl-1,2-dihydro-3H-1λ* ^*4*^ *,8λ* ^*4*^ *-[1,3,2]diazaborolo[1,5-a]pyridin-3-one* ***(3a)*** *.* The product was isolated by flash chromatography as a yellow-green solid (53.8 mg, 99%): mp: 186.4–189.4°C; ^1^H NMR (400 MHz, CDCl_3_) δ 8.41–8.34 (m, 2H), 8.19 (td, *J* = 8.0, 1.2 Hz, 1H), 7.58 (dd, *J* = 12.4, 6.4 Hz, 1H), 7.55–7.51 (m, 2H), 7.37 (dd, *J* = 7.6, 4.0 Hz, 4H), 7.27–7.20 (m, 6H), 7.16 (t, *J* = 7.9 Hz, 2H), 7.02 (dd, *J* = 1.2, 7.6 Hz, 1H). ^13^C{^1^H} NMR (101 MHz, CDCl_3_) δ 161.4, 142.4, 141.6, 140.0, 135.8, 133.7, 128.4, 127.9, 127.3, 127.2, 125.0, 124.8, 122.5, 77.5, 77.2, 76.8. HRMS (ESI), m/z calcd for C_24_H_19_BN_2_ONa^+^ (M + Na)^+^ 385.1483, found 385.1485. IR (cm^−1^): 3,074, 3,009, 1,663, 1,489, 1,348, 762, 702.

*1,1-di([1,1′-biphenyl]-4-yl)-2-phenyl-1,2-dihydro-3H-1λ* ^*4*^ *,8λ* ^*4*^ *-[1,3,2]diazaborolo[1,5-a]pyridin-3-one* ***(3b)*** *.* The product was isolated by flash chromatography as a yellow-green solid (57.9 mg, 75%): mp: 289.0–290.7°C; ^1^H NMR (400 MHz, CDCl_3_) δ 8.46 (dd, *J* = 15.6, 5.6 Hz, 2H), 8.28 (t, *J* = 7.2 Hz, 1H), 7.72–7.68 (m, 1H), 7.65–7.57 (m, 6H), 7.53–7.47 (m, 8H), 7.44–7.39 (m, 4H), 7.34–7.29 (m, 2H), 7.22 (dd, *J* = 7.6, 2.0 Hz, 2H), 7.06 (t, *J* = 7.2 Hz, 1H). ^13^C{^1^H} NMR (101 MHz, CDCl_3_) δ 161.3, 148.9, 142.4, 141.6, 141.2, 139.9, 139.8, 134.1, 128.7, 128.4, 127.2, 127.1, 127.0, 126.5, 125.0, 124.6, 122.5, 77.4, 77.2, 76.7. HRMS (ESI), m/z calcd for C_36_H_27_BN_2_ONa^+^ (M + Na)^+^ 537.2109, found 537.2111. IR (cm^−1^): 3,058, 3,015, 1,680, 1,495, 1,348, 740, 686.

*1,1-bis(3-bromophenyl)-2-phenyl-1,2-dihydro-3H-1λ* ^*4*^ *,8λ* ^*4*^ *-[1,3,2]diazaborolo[1,5-a]pyridin-3-one* ***(3c)*** The product was isolated by flash chromatography as a yellow-green solid (49.9 mg, 64%): mp: 203.5–204.5°C; ^1^H NMR (400 MHz, CDCl_3_) δ 8.48–8.40 (m, 1H), 8.38–8.28 (m, 3H), 7.78–7.72 (m, 2H), 7.43–7.35 (m, 6H), 7.25–7.19 (m, 2H), 7.17–7.08 (m, 3H). ^13^C{^1^H} NMR (101 MHz, CDCl_3_) δ 161.2, 148.6, 143.1, 141.6, 139.0, 135.9, 135.8, 132.0, 130.5, 129.8, 128.6, 127.6, 125.6, 124.9, 122.9, 77.4, 77.1, 76.8. HRMS (ESI), m/z calcd for C_24_H_17_BBr_2_N_2_ONa^+^ (M + Na)^+^ 542.9672, found 542.9680. IR (cm^−1^): 3,058, 1,674, 1,489, 1,348, 762, 692.

*2-phenyl-1,1-divinyl-1,2-dihydro-3H-1λ* ^*4*^ *,8λ* ^*4*^ *-[1,3,2]diazaborolo[1,5-a]pyridin-3-one* ***(3d)*** The product was isolated by flash chromatography as a yellow-green solid (22.4 mg, 57%): mp: 78.9–80.0°C; ^1^H NMR (400 MHz, CDCl_3_) δ 8.40 (d, *J* = 5.6 Hz, 1H), 8.34–8.22 (m, 2H), 7.93–7.85 (m, 2H), 7.75 (t, *J* = 6.0 Hz, 1H), 7.38–7.31 (m, 2H), 7.14 (t, *J* = 7.2 Hz, 1H), 6.30 (dd, *J* = 19.6, 13.2 Hz, 2H), 5.57 (dd, *J* = 13.2, 3.2 Hz, 2H), 5.32 (dd, *J* = 19.6, 3.6 Hz, 2H). ^13^C{^1^H} NMR (101 MHz, CDCl_3_) δ 160.6, 149.0, 142.1, 141.0, 140.3, 128.4, 126.5, 124.7, 124.6, 123.7, 122.3, 77.4, 77.1, 76.8. HRMS (ESI), m/z calcd for C_16_H_15_BN_2_ONa^+^ (M + Na)^+^ 285.1170, found 285.1173. IR (cm^−1^): 3,047, 2,928, 1,668, 1,495, 1,360, 757, 686.

*1,1-bis(3-fluorophenyl)-2-phenyl-1,2-dihydro-3H-1λ* ^*4*^ *,8λ* ^*4*^ *-[1,3,2]diazaborolo[1,5-a]pyridin-3-one* ***(3e)*** The product was isolated by flash chromatography as a yellow-green solid (34.6 mg, 58%): mp: 152.3–154.6°C; ^1^H NMR (400 MHz, CDCl_3_) δ 8.43 (d, *J* = 8.0 Hz, 1H), 8.37–8.27 (m, 2H), 7.72 (t, *J* = 6.4 Hz, 1H), 7.44 (d, *J* = 7.6 Hz, 2H), 7.25–7.16 (m, 4H), 7.13–7.04 (m, 3H), 7.00 (dd, *J* = 10.4, 1.2 Hz, 2H), 6.92 (td, *J* = 8.4, 2.0 Hz, 2H). ^13^C{^1^H} NMR (101 MHz, CDCl_3_) δ 164.0, 161.6, 161.2, 148.8, 143.0, 141.5, 139.3, 129.6, 129.5, 128.9, 128.9, 128.5, 127.4, 125.4, 124.8, 122.8, 119.8, 119.6, 114.3, 114.1, 77.4, 77.1, 76.8. ^19^F NMR (376 MHz, CDCl_3_) δ -113.78. HRMS (ESI), m/z calcd for C_24_H_17_BF_2_N_2_ONa^+^ (M + Na)^+^ 421.1294, found 421.1297. IR (cm^−1^): 3,068, 1,680, 1,495, 1,354, 762, 702.

*1,1-bis(4-bromophenyl)-2-phenyl-1,2-dihydro-3H-1λ* ^*4*^ *,8λ* ^*4*^ *-[1,3,2]diazaborolo[1,5-a]pyridin-3-one* ***(3f)*** The product was isolated by flash chromatography as a yellow-green solid (39.0 mg, 50%): mp: 250.0–251.3°C; ^1^H NMR (400 MHz, CDCl_3_) δ 8.42 (d, *J* = 8.0 Hz, 1H), 8.34–8.28 (m, 2H), 7.72 (t, *J* = 6.4 Hz, 1H), 7.47–7.41 (m, 2H), 7.36 (d, *J* = 8.0 Hz, 4H), 7.23–7.16 (m, 6H), 7.07 (t, *J* = 7.6 Hz, 1H). ^13^C{^1^H} NMR (101 MHz, CDCl_3_) δ 161.1, 148.8, 142.9, 141.4, 139.3, 135.2, 131.0, 128.5, 127.4, 125.4, 124.6, 122.8, 121.9, 77.4, 77.1, 76.7. HRMS (ESI), m/z calcd for C_24_H_17_BBr_2_N_2_ONa^+^ (M + Na)^+^ 542.9672, found 542.9680. IR (cm^−1^): 3,068, 1,674, 1,489, 1,354, 762, 698.

*2-phenyl-1,1-di-p-tolyl-1,2-dihydro-3H-1λ* ^*4*^ *,8λ* ^*4*^ *-[1,3,2]diazaborolo[1,5-a]pyridin-3-one* ***(3g)*** The product was isolated by flash chromatography as a yellow-green solid (36.9 mg, 63%): mp: 240.9–241.9°C; ^1^H NMR (400 MHz, CDCl_3_) δ 8.41–8.35 (m, 2H), 8.24–8.18 m, 1H), 7.64–7.56 (m, 1H), 7.28 (d, *J* = 8.0 Hz, 4H), 7.20–7.14 (m, 2H), 7.07 (d, *J* = 7.6 Hz, 4H), 7.05–7.00 (m, 1H), 2.31 (s, 6H). ^13^C{^1^H} NMR (101 MHz, CDCl_3_) δ 161.2, 148.8, 142.2, 141.5, 140.1, 136.6, 133.7, 128.6, 128.2, 127.0, 124.8, 124.6, 122.3, 77.4, 77.1, 76.8, 21.3. HRMS (ESI), m/z calcd for C_26_H_23_BN_2_ONa^+^ (M + Na)^+^ 413.1796, found 413.1799. IR (cm^−1^): 3,020, 1,674, 1,489, 1,348, 768, 686.

*1,1-bis(4-chlorophenyl)-2-phenyl-1,2-dihydro-3H-1λ* ^*4*^ *,8λ* ^*4*^ *-[1,3,2]diazaborolo[1,5-a]pyridin-3-one* ***(3h)*** The product was isolated by flash chromatography as yellow-green solid (43.3 mg, 67%): mp: 202.0–206.3°C; ^1^H NMR (400 MHz, DMSO-*d6*) *δ* 8.74 (d, *J* = 5.6 Hz, 1H), 8.56 (t, *J* = 7.6 Hz, 1H), 8.42 (d, *J* = 7.8 Hz, 1H), 7.99 (t, *J* = 6.4 Hz, 1H), 7.47 (d, *J* = 8.0 Hz, 2H), 7.35–7.25 (m, 8H), 7.19 (t, *J* = 7.6 Hz, 2H), 7.02 (t, *J* = 7.2 Hz, 1H). ^13^C{^1^H} NMR (101 MHz, DMSO-*d6*) δ 161.6, 147.3, 145.0, 142.8, 140.3, 135.5, 132.5, 129.5, 128.7, 128.1, 124.9, 124.1, 123.0, 40.6, 40.4, 40.2, 40.0, 39.8, 39.6, 39.4. HRMS (ESI), m/z calcd for C_24_H_17_BCl_2_N_2_ONa^+^ (M + Na)^+^ 453.0703, found 453.0707. IR (cm^−1^): 3,080, 1,674, 1,489, 1,360, 1,082, 768.

*1,1-bis(4-(tert-butyl)phenyl)-2-phenyl-1,2-dihydro-3H-1λ* ^*4*^ *,8λ* ^*4*^ *-[1,3,2]diazaborolo[1,5-a]pyridin-3-one* ***(3i)*** The product was isolated by flash chromatography as a yellow-green solid (45.5 mg, 64%): mp: 222.6–223.4°C; ^1^H NMR (400 MHz, CDCl_3_) δ 8.42–8.34 (m, 2H), 8.17 (td, *J* = 8.0, 1.2 Hz, 1H), 7.62–7.54 (m, 3H), 7.32 (d, *J* = 8.4 Hz, 4H), 7.28–7.23 (m, 4H), 7.20–7.13 (m, 2H), 7.05–6.97 (m, 1H), 1.29 (s, 18H). ^13^C{^1^H} NMR (101 MHz, CDCl_3_) δ 161.3, 149.7, 148.7, 142.1, 141.7, 140.2, 133.5, 128.2, 126.9, 124.7, 124.6, 124.6, 122.2, 77.4, 77.1, 76.8, 34.4, 31.4. HRMS (ESI) m/z calcd for C_32_H_35_BN_2_ONa^+^ (M + Na)^+^ 497.2735, found 497.2740. IR (cm^−1^): 3,080, 2,966, 1,674, 1,489, 1,354, 762, 686.

*1,1-bis(benzo[d][1,3]dioxol-5-yl)-2-phenyl-1,2-dihydro-3H-1λ* ^*4*^ *,8λ* ^*4*^ *-[1,3,2]diazaborolo[1,5-a]pyridin-3-one* ***(3j)*** The product was isolated by flash chromatography as a yellow-green solid (41.9 mg, 62%): mp: 227.6–229.4°C; ^1^H NMR (400 MHz, CDCl_3_) δ 8.37–8.33 (m, 2H), 8.20 (td, *J* = 8.0, 0.8 Hz, 1H), 7.65–7.59 (m, 1H), 7.58–7.52 (m, 2H), 7.23–7.16 (m, 2H), 7.04 (t, *J* = 7.6 Hz, 1H), 6.85 (dd, *J* = 7.6, 1.2 Hz, 2H), 6.79 (d, *J* = 1.2 Hz, 2H), 6.71 (d, *J* = 8.0 Hz, 2H), 5.85 (s, 4H). ^13^C{^1^H} NMR (101 MHz, CDCl_3_) δ 161.1, 148.6, 147.3, 146.8, 142.5, 141.5, 139.8, 128.3, 127.1, 125.0, 124.6, 122.4, 113.1, 108.2, 100.4, 77.4, 77.1, 76.8. HRMS (ESI) m/z calcd for C_26_H_19_BN_2_O_5_Na^+^ (M + Na)^+^ 473.1279, found 473.1281. IR (cm^−1^): 3,063, 2,884, 1,680, 1,484, 1,348, 1,234, 1,039, 768.

*1,1-bis(4-fluorophenyl)-2-phenyl-1,2-dihydro-3H-1λ* ^*4*^ *,8λ* ^*4*^ *-[1,3,2]diazaborolo[1,5-a]pyridin-3-one* ***(3k)*** The product was isolated by flash chromatography as a yellow-green solid (46.6 mg, 78%): mp: 176.1–177.9°C; ^1^H NMR (400 MHz, CDCl_3_) δ 8.40 (d, *J* = 7.6 Hz, 1H), 8.33 (d, *J* = 5.6 Hz, 1H), 8.31–8.24 (m, 1H), 7.71–7.65 (m, 1H), 7.49–7.44 (m, 2H), 7.34–7.27 (m, 4H), 7.22–7.15 (m, 2H), 7.08–7.02 (m, 1H), 6.96–6.90 (m, 4H). ^13^C{^1^H} NMR (101 MHz, CDCl_3_) δ 163.8, 161.3, 161.1, 148.7, 142.7, 141.4, 139.7, 135.2, 135.1, 128.7, 128.4, 127.3, 125.2, 124.6, 122.6, 114.9, 114.7, 77.4, 77.1, 76.8. ^19^F NMR (376 MHz, CDCl_3_) δ -113.78. HRMS (ESI) m/z calcd for C_24_H_17_BF_2_N_2_ONa^+^ (M + Na)^+^ 421.1294, found 421.1298. IR(cm^−1^): 3,042, 1,680, 1,495, 1,364, 1,164, 774.

*1,1-bis(3-(benzyloxy)phenyl)-2-phenyl-1,2-dihydro-3H-1λ* ^*4*^ *,8λ* ^*4*^ *-[1,3,2]diazaborolo[1,5-a]pyridin-3-one* ***(3l)*** The product was isolated by flash chromatographyas a yellow-green solid (60.3 mg, 70%): mp: 72.6–74.2°C; ^1^H NMR (400 MHz, CDCl_3_) δ 8.36 (d, *J* = 7.6 Hz, 1H), 8.26 (d, *J* = 5.6 Hz, 1H), 8.20 (td, *J* = 8.0, 1.2 Hz, 1H), 7.59–7.50 (m, 3H), 7.38–7.27 (m, 10H), 7.23–7.15 (m, 4H), 7.07 (t, *J* = 7.2 Hz, 1H), 6.98–6.94 (m, 4H), 6.90–6.84 (m, 2H), 4.97–4.89 (m, 4H). ^13^C{^1^H} NMR (101 MHz, CDCl_3_) δ 161.2, 158.3, 148.7, 142.4, 141.6, 139.9, 137.3, 128.9, 128.5, 128.4, 127.8, 127.6, 127.1, 126.3, 125.1, 124.9, 122.4, 120.0, 113.5, 77.4, 77.1, 76.8, 69.8. HRMS (ESI) m/z calcd for C_38_H_31_BN_2_O_3_Na^+^ (M + Na)^+^ 597.2320, found 597.2322. IR (cm^−1^): 3,063, 3,030, 1,680, 1,489, 1,348, 1,229, 762, 692.

*1,1-di(naphthalen-2-yl)-2-phenyl-1,2-dihydro-3H-1λ* ^*4*^ *,8λ* ^*4*^ *-[1,3,2]diazaborolo[1,5-a]pyridin-3-one* ***(3m)*** The product was isolated by flash chromatography as a yellow-green solid (63.8 mg, 92%): mp: 215.5–217.0°C; ^1^H NMR (400 MHz, CDCl_3_) δ 8.44 (dd, *J* = 4.8, 4.0 Hz, 2H), 8.20 (td, *J* = 7.6, 0.8 Hz, 1H), 7.98–7.95 (m, 2H), 7.83–7.78 (m, 2H), 7.77–7.71 (m, 4H), 7.65–7.61 (m, 2H), 7.60–7.55 (m, 1H), 7.51 (dd, *J* = 8.4, 1.2 Hz, 2H), 7.48–7.40 (m, 4H), 7.19–7.12 (m, 2H), 7.05–6.98 (m, 1H). ^13^C{^1^H} NMR (101 MHz, CDCl_3_) δ 161.4, 148.9, 142.5, 141.7, 139.9, 133.5, 133.4, 133.0, 131.0, 128.4, 128.1, 127.6, 127.1,127.1, 125.7, 125.6, 125.1, 124.8, 122.6, 77.4, 77.1, 76.8. HRMS (ESI) m/z calcd for C_32_H_23_BN_2_ONa^+^ (M + Na)^+^ 485.1796, found 485.1797. IR (cm^−1^): 3,047, 1,668, 1,495, 1,354, 762, 469.

*1,1-bis(3-methoxyphenyl)-2-phenyl-1,2-dihydro-3H-1λ* ^*4*^ *,8λ* ^*4*^ *-[1,3,2]diazaborolo[1,5-a]pyridin-3-one* ***(3n)*** The product was isolated by flash chromatography as a yellow-green solid (40.5 mg, 64%): mp: 170.9–172.2°C; ^1^H NMR (400 MHz, CDCl_3_) δ 8.378 (t, *J* = 5.2 Hz, 2H), 8.20 (td, *J* = 400, 1.2 Hz, 1H), 7.64–7.58 (m, 1H), 7.55–7.49 (m, 2H), 7.21–7.15 (m, 4H), 7.07–7.01 (m, 1H), 6.96–6.89 (m, 4H), 6.79–6.75 (m, 2H), 3.67 (s, 6H). ^13^C{^1^H} NMR (101 MHz, CDCl_3_) δ 161.3, 159.1, 148.7, 142.5, 141.7, 139.9, 128.9, 128.3, 127.1, 125.9, 125.1, 124.9, 122.4, 119.4, 112.2, 77.5, 77.1, 76.8, 55.0. HRMS (ESI) m/z calcd for C_26_H_23_BN_2_O_3_Na^+^ (M + Na)^+^ 445.1694, found 445.1699. IR (cm^−1^): 3,009, 1,668, 1,343, 1,229, 762, 757.

*1,1-bis(2-methoxyphenyl)-2-phenyl-1,2-dihydro-3H-1λ* ^*4*^ *,8λ* ^*4*^ *-[1,3,2]diazaborolo[1,5-a]pyridin-3-one* ***(3o)*** The product was isolated by flash chromatography as a yellow-green solid (22.2 mg, 35%): mp: 249.1–250.7°C; ^1^H NMR (400 MHz, CDCl_3_) δ 9.13 (d, *J* = 5.6 Hz, 1H), 8.29 (d, *J* = 7.6 Hz, 1H), 8.15 (t, *J* = 6.8 Hz, 1H), 7.62 (d, *J* = 7.6 Hz, 2H), 7.55 (t, *J* = 6.0 Hz, 1H), 7.43–7.39 (m, 2H), 7.23–7.18 (m, 2H), 7.12 (t, *J* = 7.6 Hz, 2H), 6.95 (t, *J* = 7.6 Hz, 1H), 6.84 (t, *J* = 7.2 Hz, 2H), 6.76 (d, *J* = 8.0 Hz, 2H), 3.48 (s, 6H). ^13^C{^1^H} NMR (101 MHz, CDCl_3_) δ 162.7, 162.3, 149.6, 142.7, 141.3, 140.9, 137.2, 128.6, 127.9, 125.9, 124.1, 124.0, 121.3, 120.3, 110.4, 77.4, 77.1, 76.8, 54.8. HRMS (ESI) m/z calcd for C_26_H_23_BN_2_O_3_Na^+^ (M + Na)^+^ 445.1694, found 445.1695. IR (cm^−1^): 3,058, 2,933, 1,674, 1,495, 1,360, 1,234, 757.

*2-phenyl-1,1-bis(4-(trifluoromethyl)phenyl)-1,2-dihydro-3H-1λ* ^*4*^ *,8λ* ^*4*^ *-[1,3,2]diazaborolo[1,5-a]pyridin-3-one* ***(3p)*** The product was isolated by flash chromatography as a yellow-green solid (34.4 mg, 46%): mp: 230.3–232.0°C; ^1^H NMR (400 MHz, CDCl_3_) δ 8.47 (d, *J* = 7.6 Hz, 1H), 8.37 (dd, *J* = 7.6, 1.2 Hz, 1H), 8.35–8.31 (m, 1H), 7.79–7.74 (m, 1H), 7.50 (d, *J* = 8.0 Hz, 4H), 7.46–7.40 (m, 6H), 7.24–7.18 (m, 2H), 7.09 (t, *J* = 7.6 Hz, 1H). ^13^C{^1^H} NMR (101 MHz, CDCl_3_) *δ* 161.1, 148.9, 143.2, 141.4, 139.1, 133.6, 129.8, 129.4, 129.1, 128.6, 127.5, 125.7, 125.5, 124.6, 124.6, 123.0, 123.0, 77.4, 77.0, 76.7. ^19^F NMR (376 MHz, CDCl_3_) δ -62.58. HRMS (ESI) m/z calcd for C_26_H_17_BF_6_N_2_ONa^+^ (M + Na)^+^ 521.1230, found 521.1236. IR (cm^−1^): 3,074, 3,036, 1,691, 1,327, 1,109, 762.

*1,1-bis(4-(methylthio)phenyl)-2-phenyl-1,2-dihydro-3H-1λ* ^*4*^ *,8λ* ^*4*^ *-[1,3,2]diazaborolo[1,5-a]pyridin-3-one* ***(3q)*** The product was isolated by flash chromatography as a yellow-green solid (44.3 mg, 65%): mp: 242.1–243.6°C; ^1^H NMR (400 MHz, CDCl_3_) δ 8.37–8.29 (m, 2H), 8.21 (td, *J* = 7.6, 0.8 Hz, 1H), 7.64–7.57 (m, 1H), 7.53–7.47 (m, 2H), 7.26–7.22 (m, 4H), 7.18–7.12 (m, 2H), 7.11–7.08 (m, 4H), 7.04–6.98 (m, 1H), 2.40 (s, 6H). ^13^C{^1^H} NMR (101 MHz, CDCl_3_) δ 161.2, 148.7, 142.5, 141.4, 139.8, 137.2, 134.1, 128.4, 127.1, 125.9, 125.0, 124.5, 122.5, 77.4, 77.1, 76.8, 15.5. HRMS (ESI) m/z calcd for C_26_H_23_BN_2_OS_2_Na^+^ (M + Na)^+^ 477.1237, found 477.1238. IR (cm^−1^): 3,063, 2,916, 1,674, 1,495, 1,375, 1,088, 762.

*1,1-bis(2-fluorophenyl)-2-phenyl-1,2-dihydro-3H-1λ* ^*4*^ *,8λ* ^*4*^ *-[1,3,2]diazaborolo[1,5-a]pyridin-3-one* ***(3r)*** The product was isolated by flash chromatography as a yellow-green solid (29.3 mg, 49%): mp: 222.9–224.0°C; ^1^H NMR (400 MHz, CDCl_3_) δ 8.85 (d, *J* = 6.0 Hz, 1H), 8.36 (d, *J* = 7.6 Hz, 1H), 8.25 (td, *J* = 8.0, 1.2 Hz, 1H), 7.10–766 (m, 1H), 7.65–7.60 (m, 2H), 7.48–7.42 (m, 2H), 7.25–7.20 (m, 2H), 7.20–7.15 (m, 2H), 7.06–7.01 (m, 3H), 6.92–6.86 (m, 2H). ^13^C{^1^H} NMR (101 MHz, CDCl_3_) δ 167.3, 164.9, 161.6, 149.0, 142.5, 142.0, 140.0, 136.9, 136.8, 129.8, 129.7, 128.3, 127.2, 124.6, 123.9, 123.8, 123.8, 122.4, 115.2, 115.0, 77.4, 77.1, 76.7. ^19^F NMR (376 MHz, CDCl_3_) δ -103.33. HRMS (ESI) m/z calcd for C_24_H_17_BF_2_N_2_ONa^+^ (M + Na)^+^ 421.1294, found 421.1298. IR (cm^−1^): 3,068, 1,668, 1,495, 1,354, 762, 746.

*1,1-bis(3-(tert-butyl)phenyl)-2-phenyl-1,2-dihydro-3H-1λ* ^*4*^ *,8λ* ^*4*^ *-[1,3,2]diazaborolo[1,5-a]pyridin-3-one* **(3s)** The product was isolated by flash chromatography as a yellow-green solid (55.5 mg, 78%): mp: 78.1–79.8°C; ^1^H NMR (400 MHz, CDCl_3_) δ 8.45–8.37 (m, 2H), 8.21 (td, *J* = 7.6, 0.8 Hz, 1H), 7.66–7.55 (m, 3H), 7.51–7.45 (m, 2H), 7.32–7.27 (m, 2H), 7.23–7.14 (m, 4H), 7.08–7.01 (m, 3H), 1.25 (s, 18H).^13^C{^1^H} NMR (101 MHz, CDCl_3_) *δ* 161.3, 149.9, 148.9, 142.3, 141.7, 140.0, 131.6, 130.2, 128.2, 127.4, 127.0, 125.1, 125.0, 123.9, 122.4, 77.5, 77.1, 76.8, 34.7, 31.5. HRMS (ESI) m/z calcd for C_32_H_35_BN_2_ONa^+^ (M + Na)^+^ 497.2735, found 497.2740. IR (cm^−1^): 2,966, 1,680, 1,489, 1,354, 757, 713.

*2,2′-(3-oxo-2-phenyl-2,3-dihydro-1H-1λ* ^*4*^ *,8λ* ^*4*^ *-[1,3,2]diazaborolo[1,5-a]pyridine-1,1-diyl)dibenzonitrile* ***(3t)*** The product was isolated by flash chromatography as a yellow-green solid (33.4 mg, 54%): mp: 252.9–253.7°C; ^1^H NMR (400 MHz, CDCl_3_) δ 8.44–8.40 (m, 1H), 8.34–8.29 (m, 2H), 7.75–7.69 (m, 1H), 7.47–7.42 (m, 2H), 7.39–7.34 (m, 4H), 7.22–7.16 (m, 6H), 7.10–7.04 (m, 1H). ^13^C{^1^H} NMR (101 MHz, CDCl_3_) δ 161.0, 148.8, 142.9, 141.4, 139.4, 135.2, 131.0, 128.5, 127.3, 125.3, 124.6, 122.8, 121.9, 77.4, 77.1, 76.7. HRMS (ESI) m/z calcd for C_26_H_18_BN_4_O+ (M + H)^+^ 413.1568, found 413.1563. IR (cm^−1^): 3,074, 3,025, 1,674, 1,489, 1,348, 762.

*1,1-bis(dibenzo[b,d]furan-4-yl)-2-phenyl-1,2-dihydro-3H-1λ* ^*4*^ *,8λ* ^*4*^ *-[1,3,2]diazaborolo[1,5-a]pyridin-3-one* ***(3u)*** The product was isolated by flash chromatography as a yellow-green solid (20.3 mg, 25%): mp: 240.3–241.9°C; ^1^H NMR (400 MHz, CDCl_3_) δ 9.12 (d, *J* = 5.6 Hz, 1H), 8.55 (d, *J* = 7.6 Hz, 1H), 8.31 (t, *J* = 7.6 Hz, 1H), 7.98–7.90 (m, 4H), 7.71–7.64 (m, 4H), 7.60–7.54 (m, 1H), 7.39–7.34 (m, 2H), 7.33–7.29 (m, 3H), 7.29–7.27 (m, 1H), 7.24 (d, *J* = 7.6 Hz, 2H), 7.15 (t, *J* = 7.9 Hz, 2H), 7.00 (t, *J* = 7.2 Hz, 1H).^13^C{^1^H} NMR (101 MHz, CDCl_3_) δ 162.0, 159.6, 155.5, 149.8, 142.6, 142.3, 140.3, 134.7, 128.2, 126.8, 126.5, 124.6, 124.5, 124.3, 123.2, 122.6, 122.4, 122.1, 120.4, 120.2, 111.3, 77.4, 77.1, 76.7. HRMS (ESI) m/z calcd for C_36_H_23_BN_2_O_3_Na^+^ (M + Na)^+^ 565.1694, found 565.1697. IR (cm^−1^): 3,053, 1,685, 1,446, 1,348, 1,175, 762.

*2-(naphthalen-1-yl)-1,1-diphenyl-1,2-dihydro-3H-1λ* ^*4*^ *,8λ* ^*4*^ *-[1,3,2]diazaborolo[1,5-a]pyridin-3-one* ***(3v)*** The product was isolated by flash chromatography as a yellow-green solid (49.5 mg, 80%): mp: 251.2–252.5°C; ^1^H NMR (400 MHz, CDCl_3_) δ 8.48 (d, *J* = 8.0 Hz, 1H), 8.38 (d, *J* = 5.6 Hz, 1H), 8.30 (t, *J* = 7.2 Hz, 1H), 7.75–7.67 (m, 3H), 7.58–7.32 (m, 3H), 7.32–7.27 (m, 3H), 7.23–7.15 (m, 3H), 7.14–6.78 (m, 6H). ^13^C{^1^H} NMR (101 MHz, CDCl_3_) δ 161.4, 148.9, 142.6, 142.5, 136.0, 134.2, 129.7, 128.0, 127.3, 127.1, 125.4, 125.3, 125.2, 124.2, 124.1, 122.8, 77.4, 77.1, 76.7. HRMS (ESI) m/z calcd for C_28_H_21_BN_2_ONa^+^ (M + Na)^+^ 435.1639, found 435.1636. IR (cm^−1^): 3,047, 1,674, 1,398, 1,354, 774, 702.

*1,1-bis(4-(benzyloxy)phenyl)-2-phenyl-1,2-dihydro-3H-1λ* ^*4*^ *,8λ* ^*4*^ *-[1,3,2]diazaborolo[1,5-a]pyridin-3-one* ***(3w)*** The product was isolated by flash chromatography as a yellow-green solid (60.3 mg, 70%): mp: 83.7–85.6°C; ^1^H NMR (400 MHz, CDCl_3_) δ 8.37 (d, *J* = 7.2 Hz, 2H), 8.22 (t, *J* = 8.0 Hz, 1H), 7.66–7.54 (m, 3H), 7.44–7.35 (m, 8H), 7.35–7.28 (m, 6H), 7.19 (t, *J* = 7.6 Hz, 2H), 7.05 (t, *J* = 7.6 Hz, 1H), 6.89 (d, *J* = 8.4 Hz, 4H), 5.02 (s, 4H). ^13^C{^1^H} NMR (101 MHz, CDCl_3_) δ 161.2, 158.2, 148.8, 142.2, 141.5, 140.1, 137.3, 134.9, 128.6, 128.3, 127.9, 127.6, 127.0, 124.8, 124.6, 122.3, 114.2, 77.4, 77.2, 76.8, 69.8. HRMS (ESI) m/z calcd for C_38_H_31_BN_2_O_3_Na^+^ (M + Na)^+^ 597.2320, found 597.2322. IR (cm^−1^): 3,020, 1,674, 1,592, 1,170, 698.

## Data Availability

The original contributions presented in the study are included in the article/Supplementary Material, further inquiries can be directed to the corresponding authors. Additional ^1^H and ^13^C spectra and spectral data for all compounds are available free of charge at http://pubs.acs.org.
