# Supplementary material for: The Synthesis of Picolinamide-Supported Tetracoordinated Organoboron Complexes with Aggregation-Induced Emission Property
Source: Front Chem. 2022 Mar 22;10:856832. doi: 10.3389/fchem.2022.856832 (PMC8980608; doi:10.3389/fchem.2022.856832)
Supplement: Supplementary file 1 [file DataSheet1.docx]

Supplementary Material

**Content**

[1 NMR spectra S3](#_Toc87581563)

[2 Fluorescence decay profiles S58](#_Toc87581564)

[3 Seven samples: solid and liquid quantum yields S63](#_Toc87581565)

[4 Seven samples: solid and liquid fluorescence lifetimes S71](#_Toc87581566)

[5 Cartesian coordinates S79](#_Toc87581567)

1. **NMR spectra**


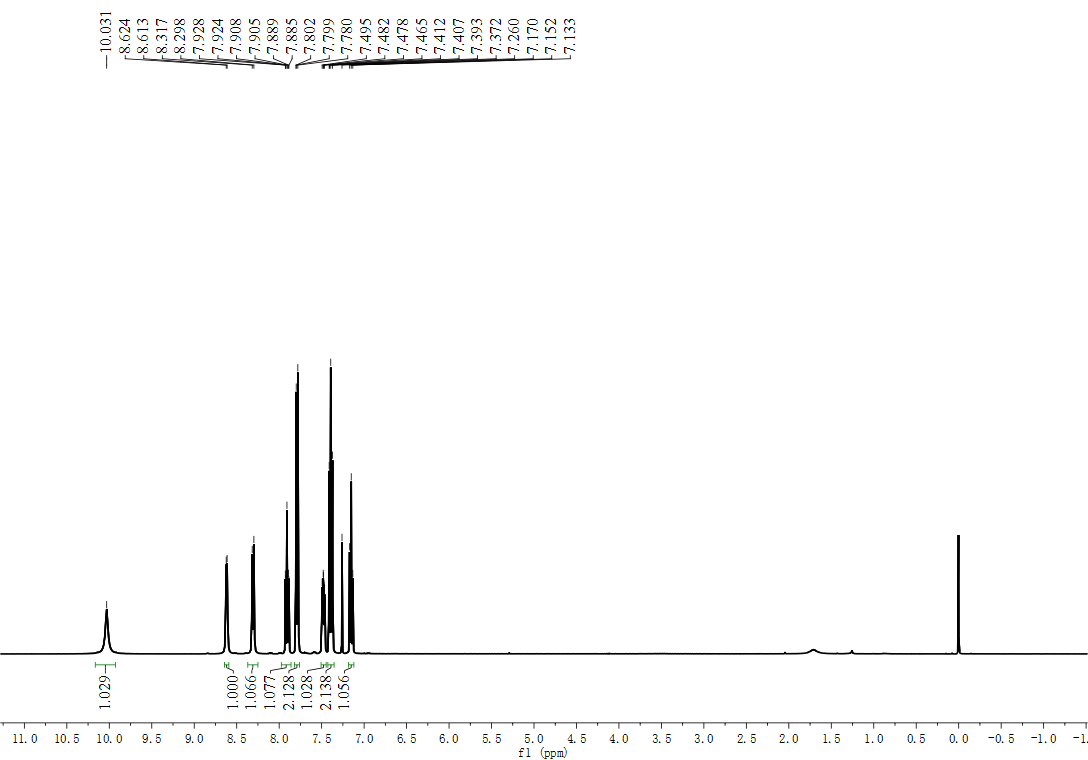


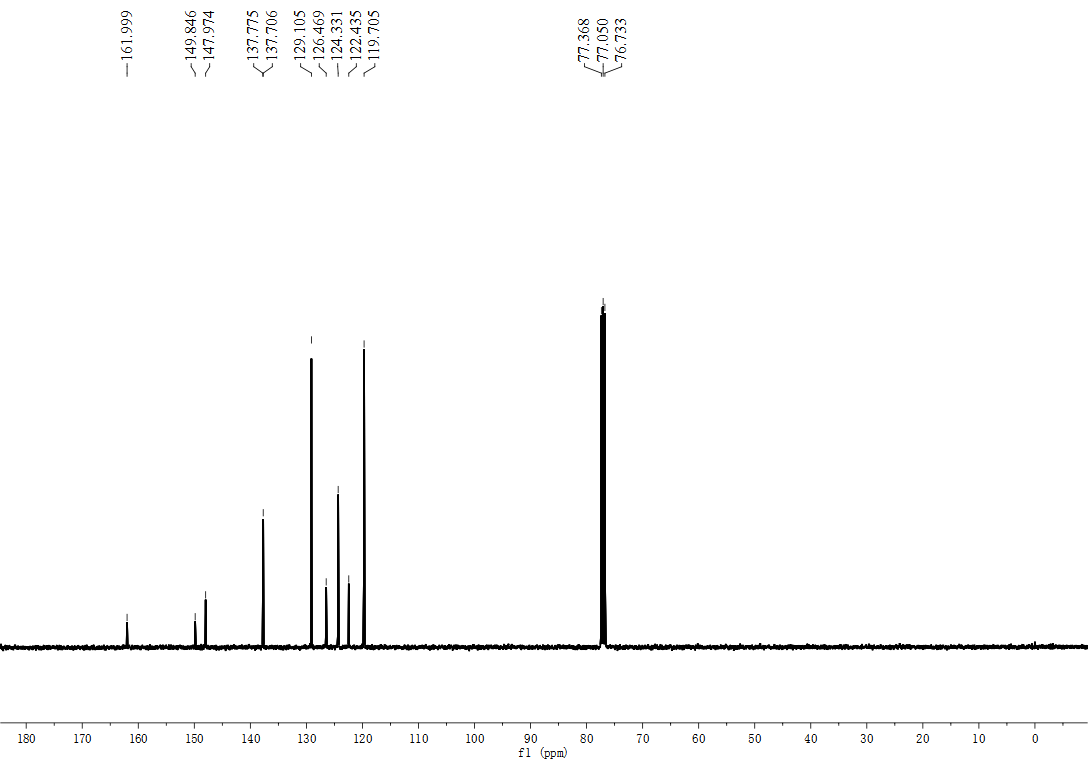


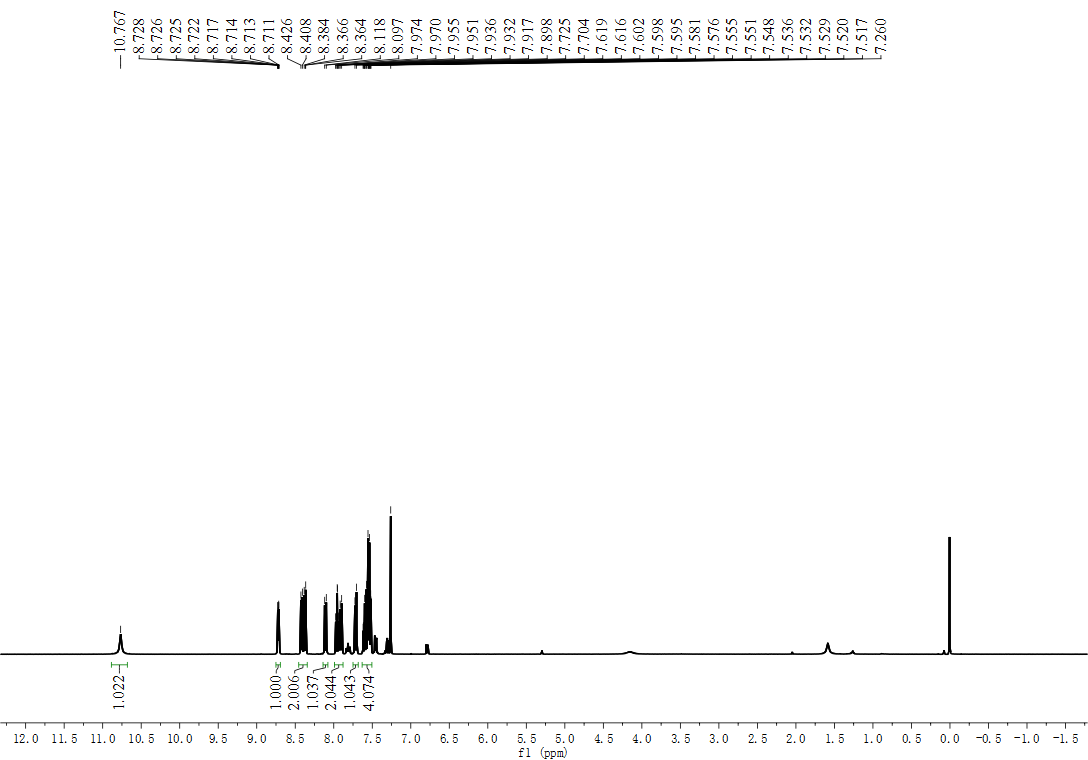


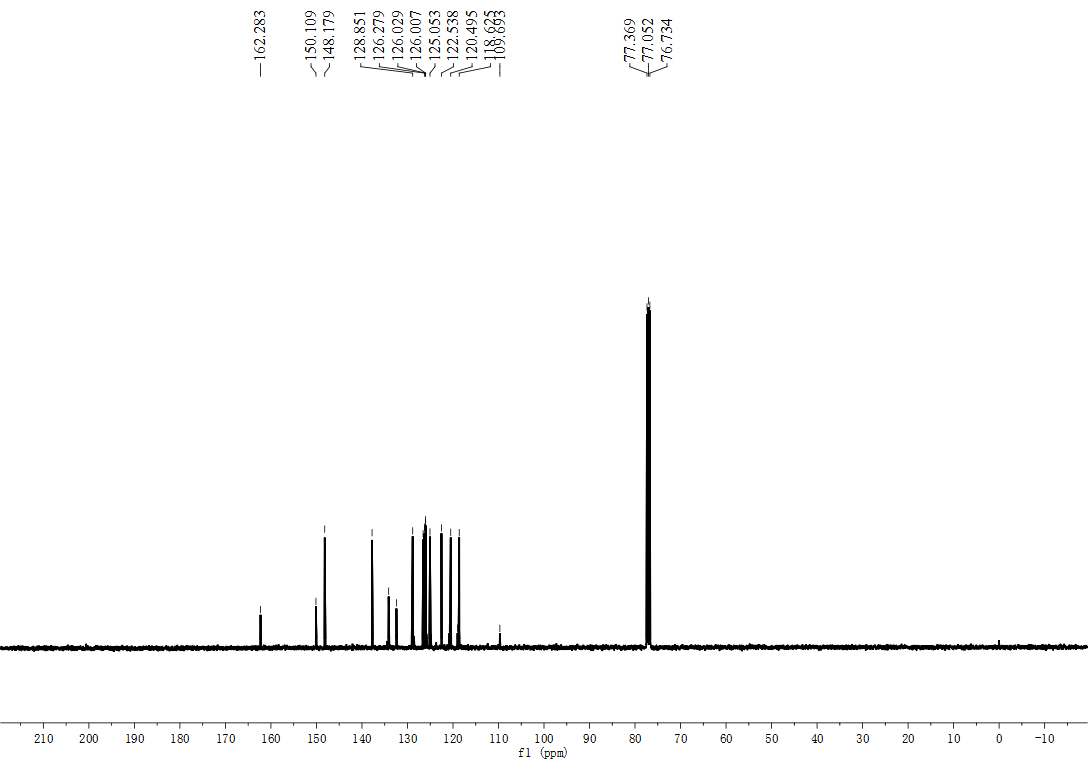


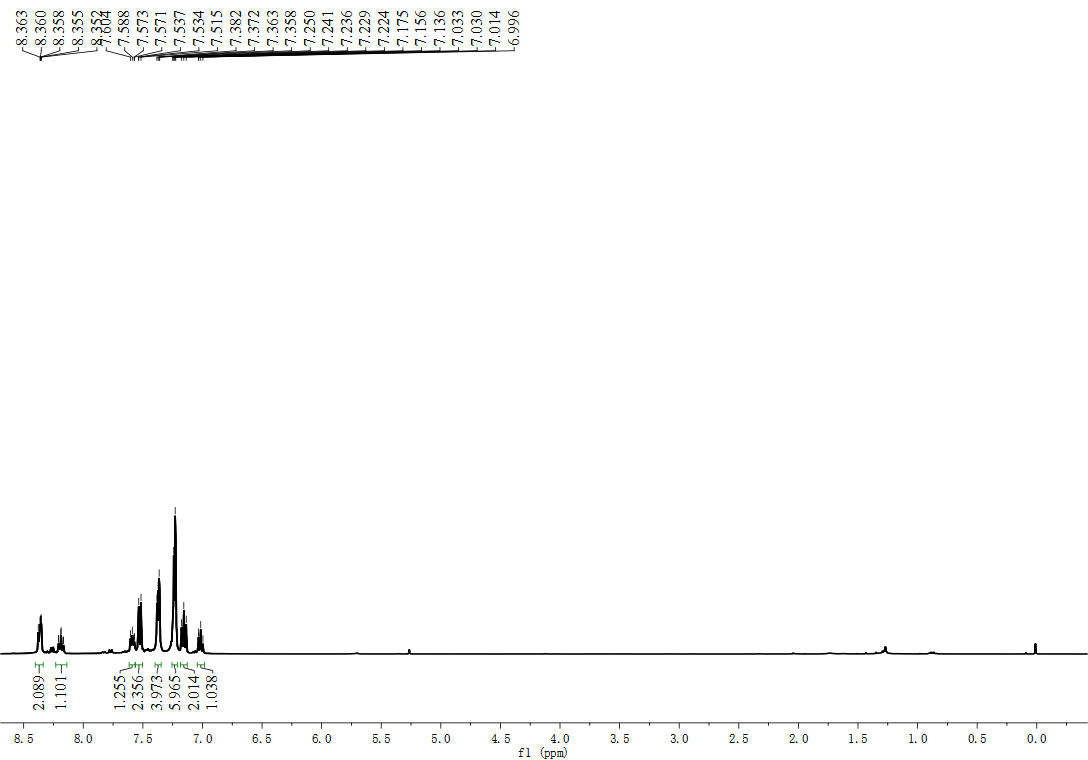


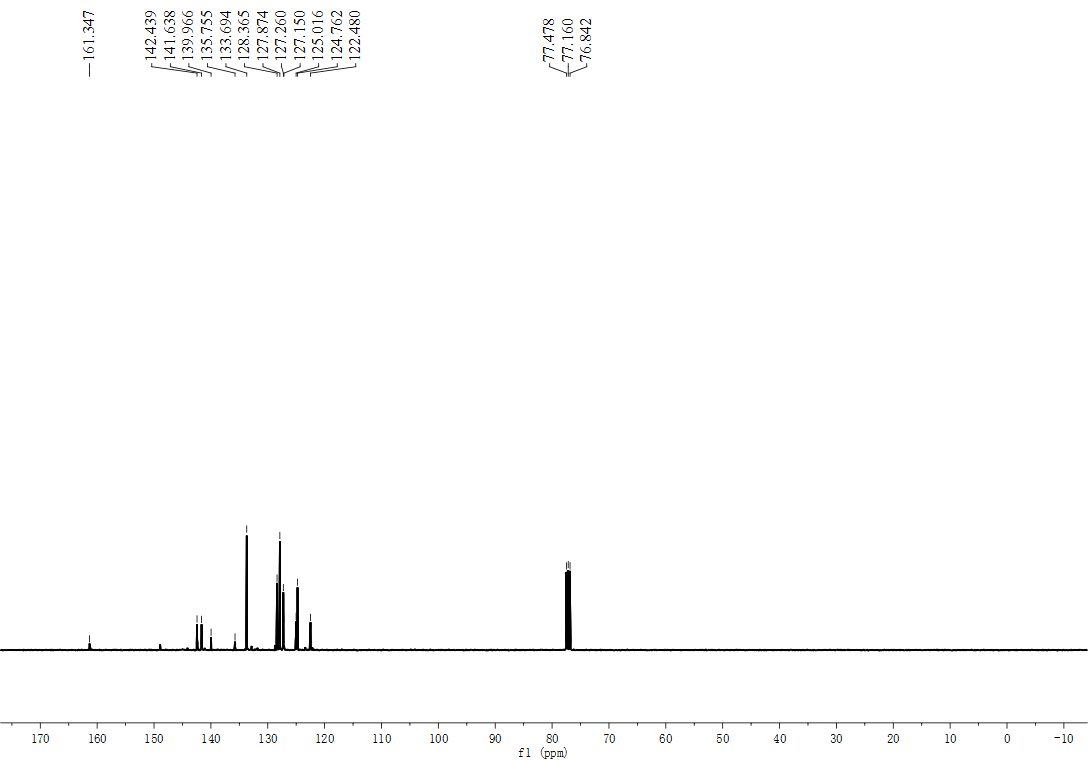


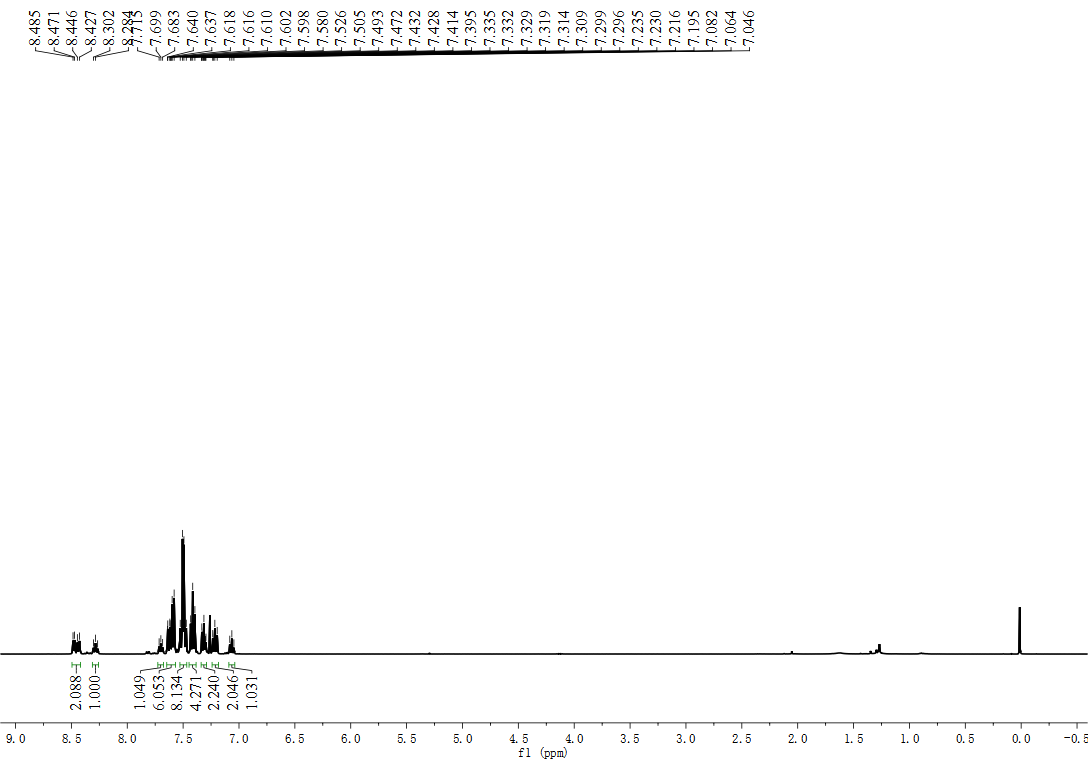


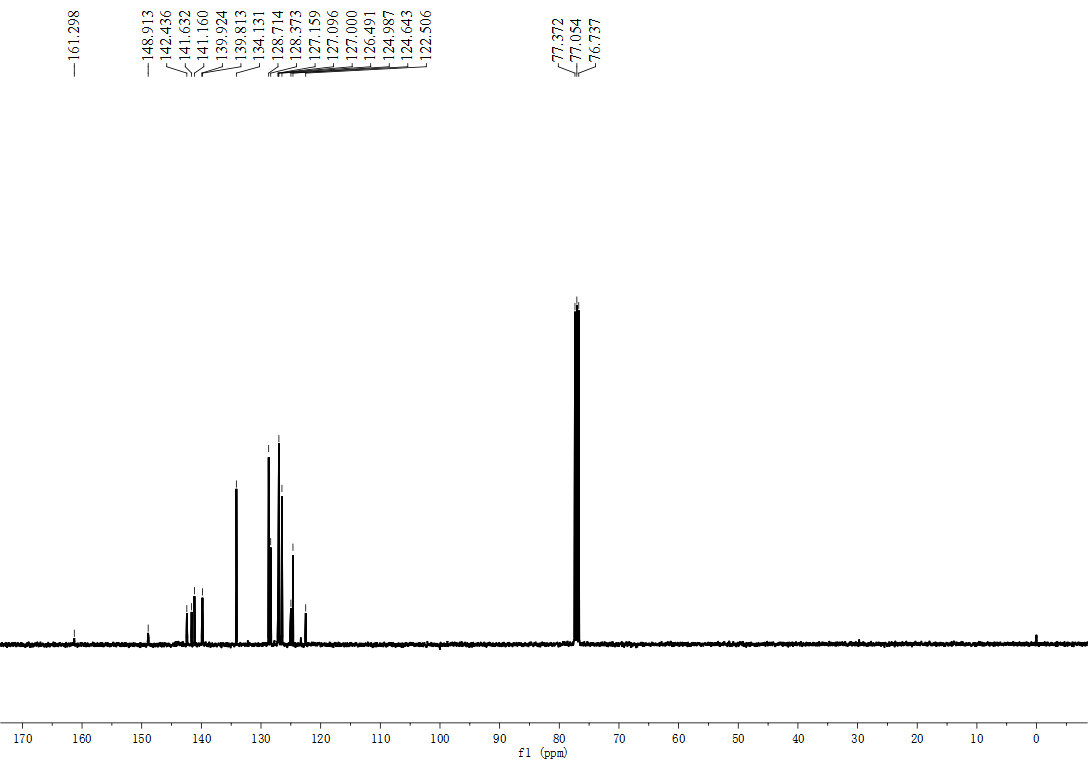


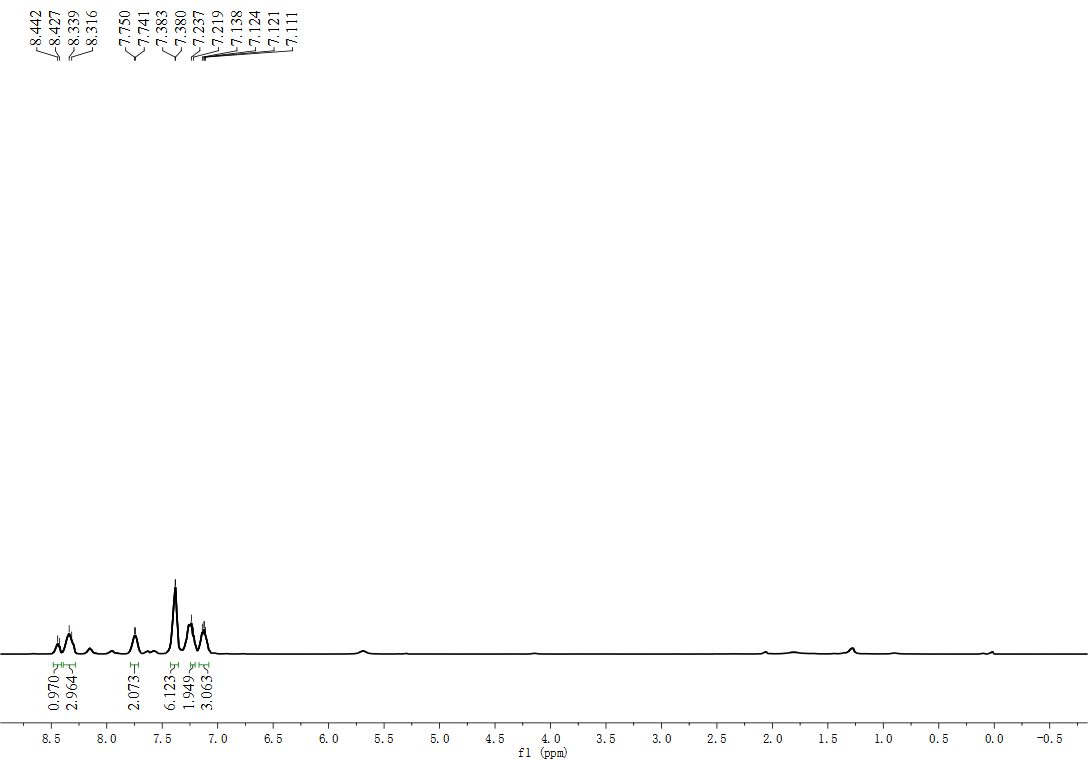


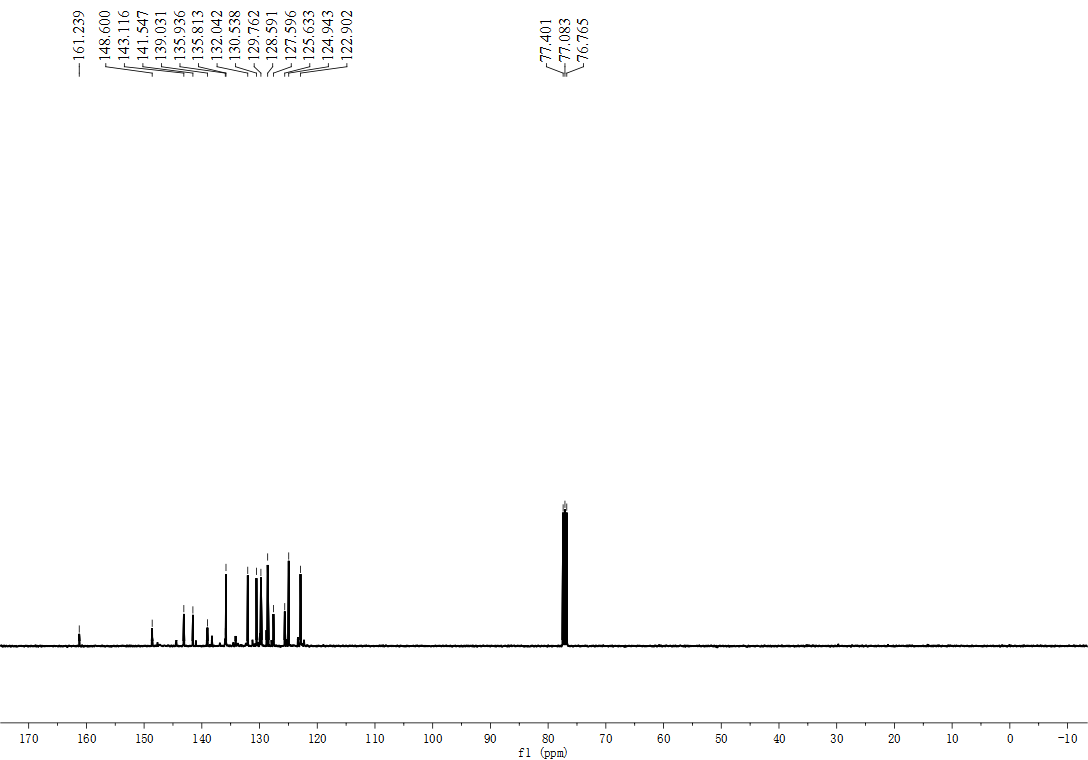


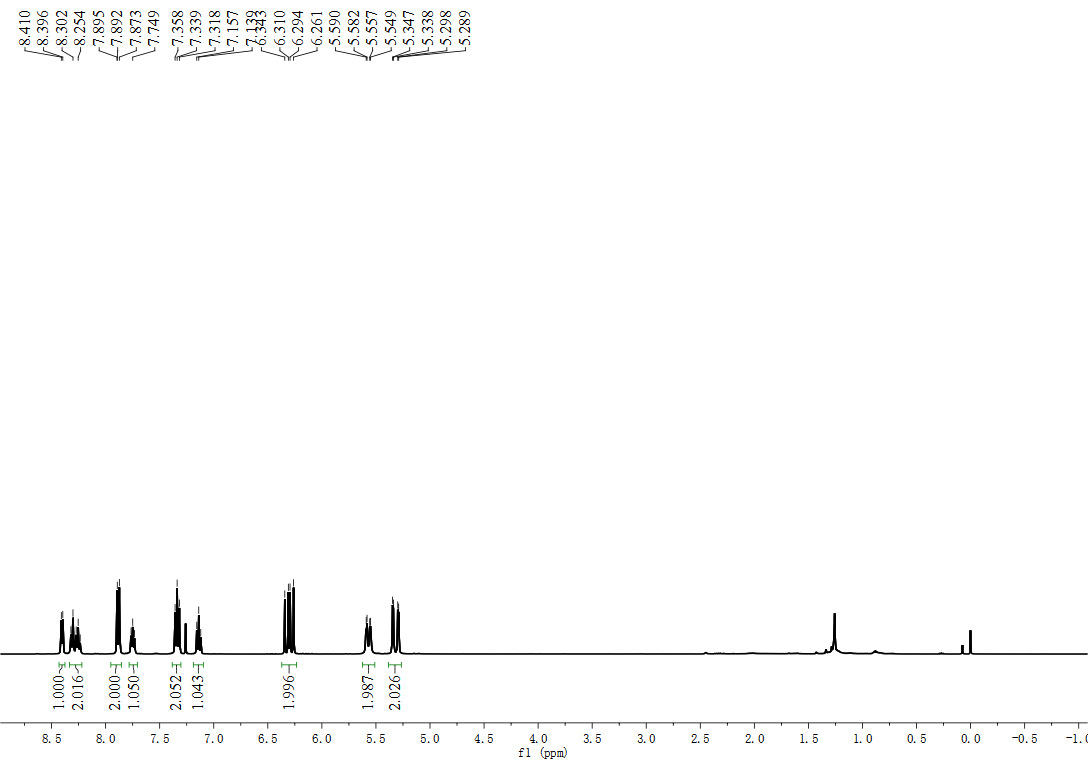


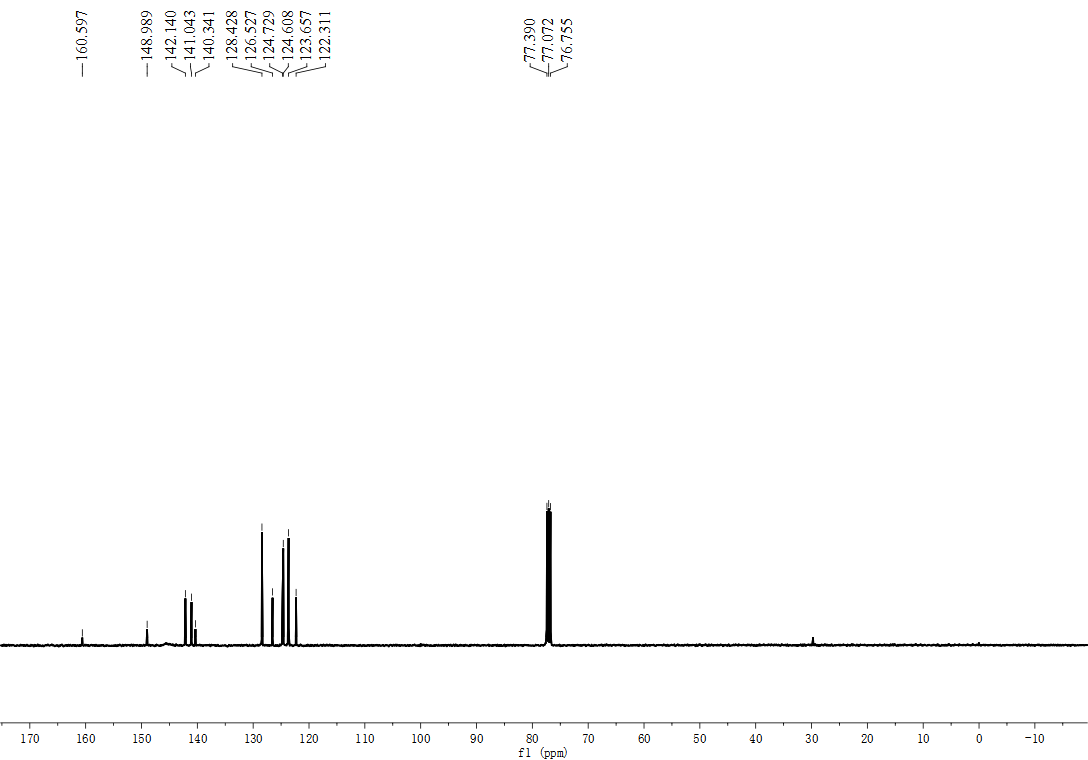


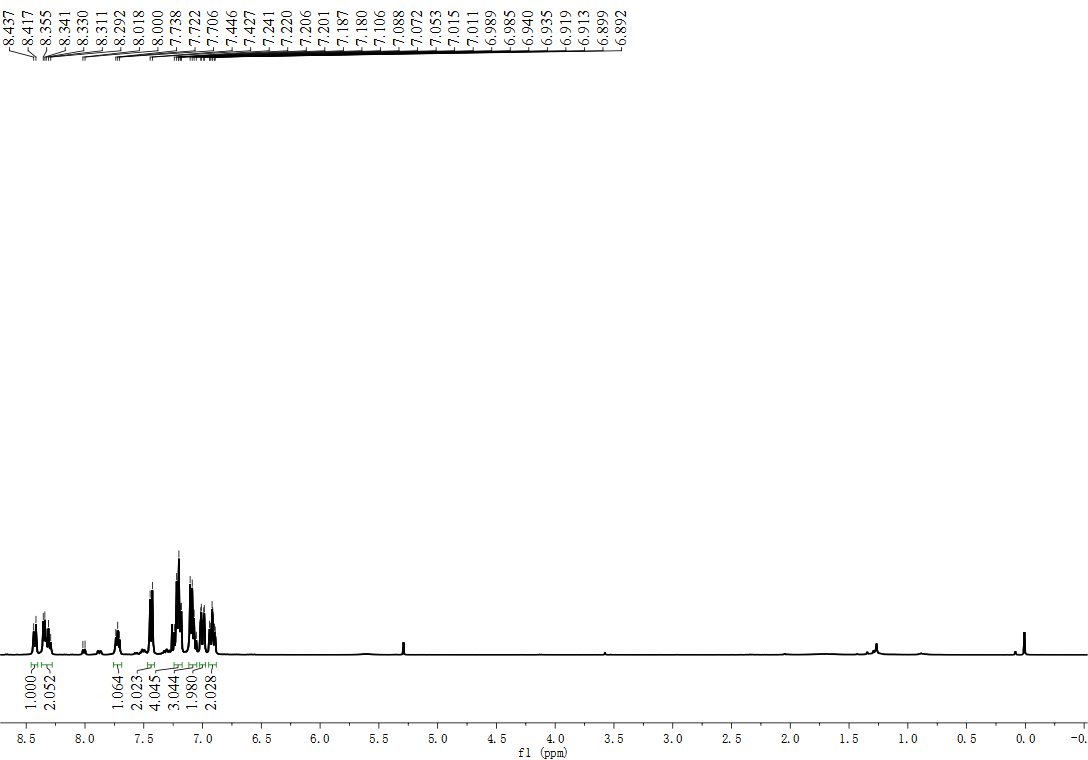


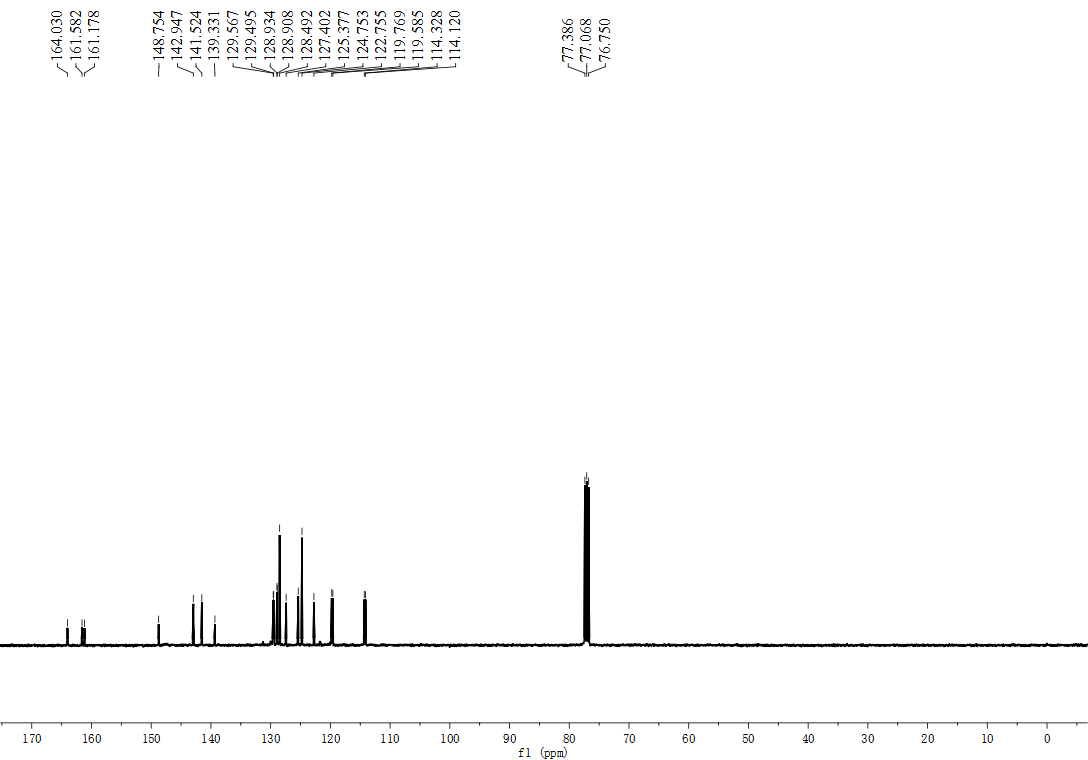


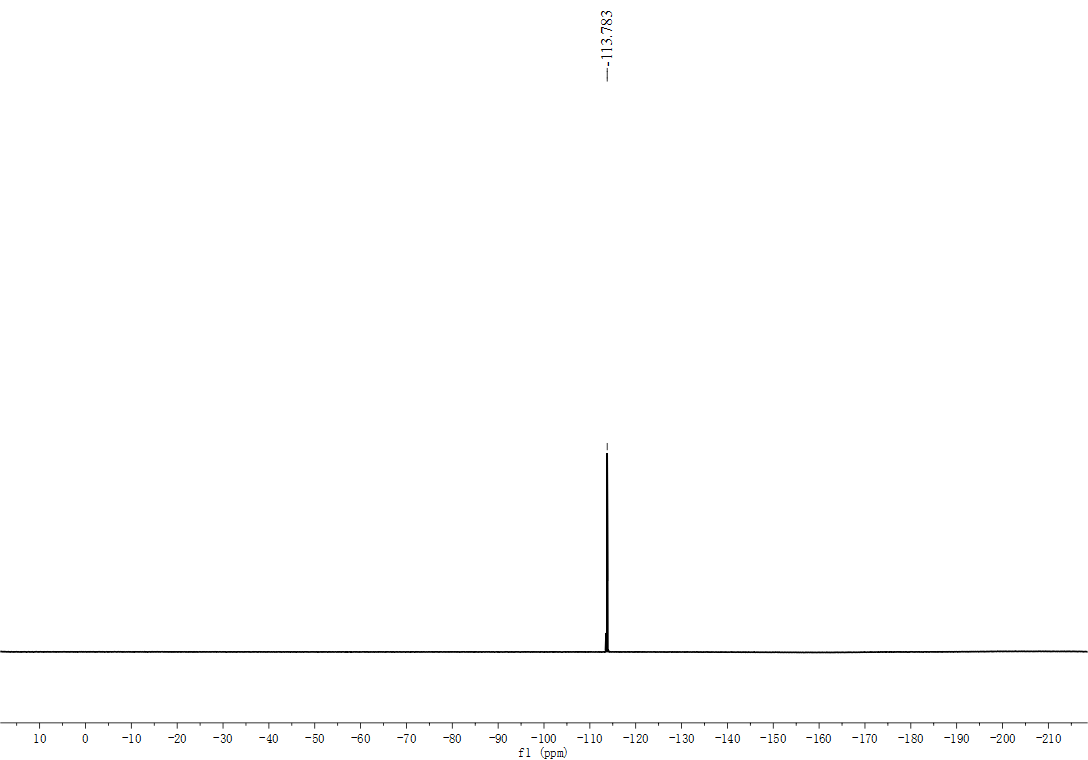


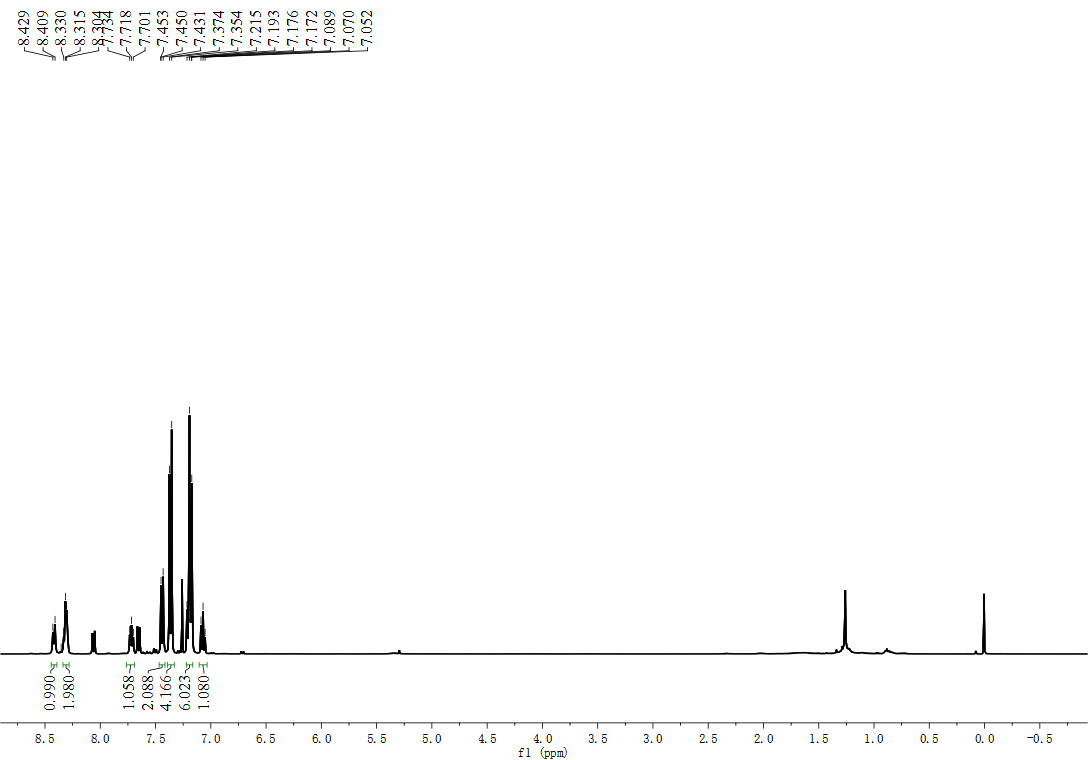


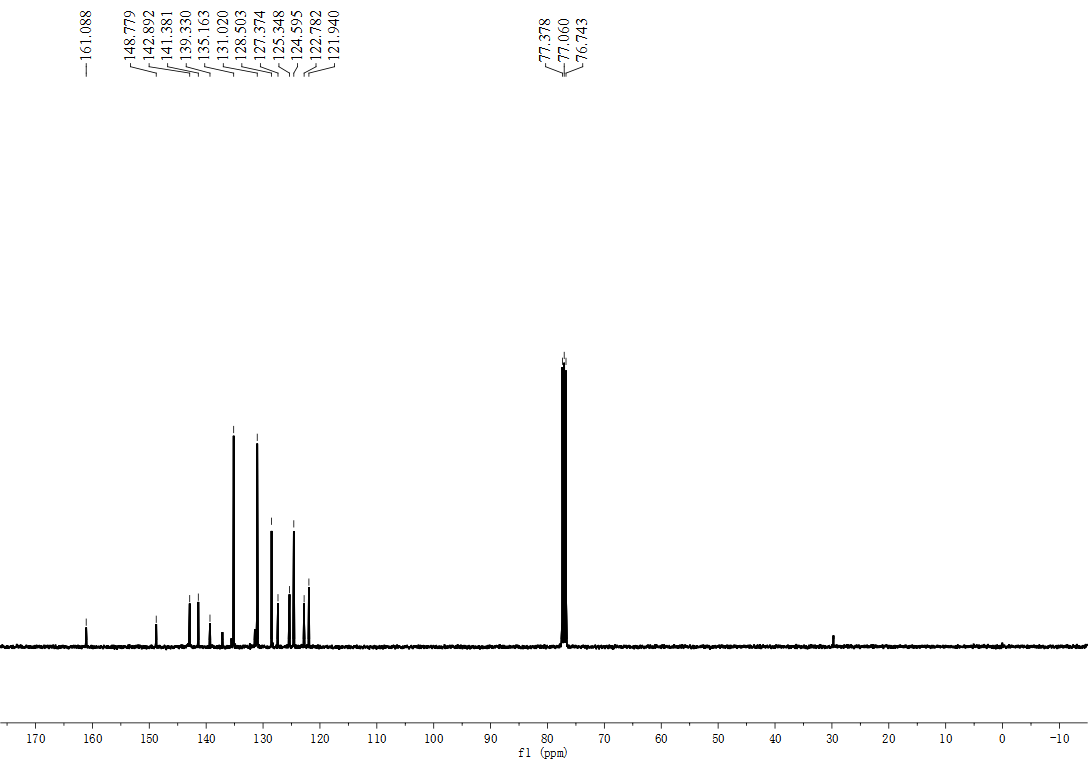


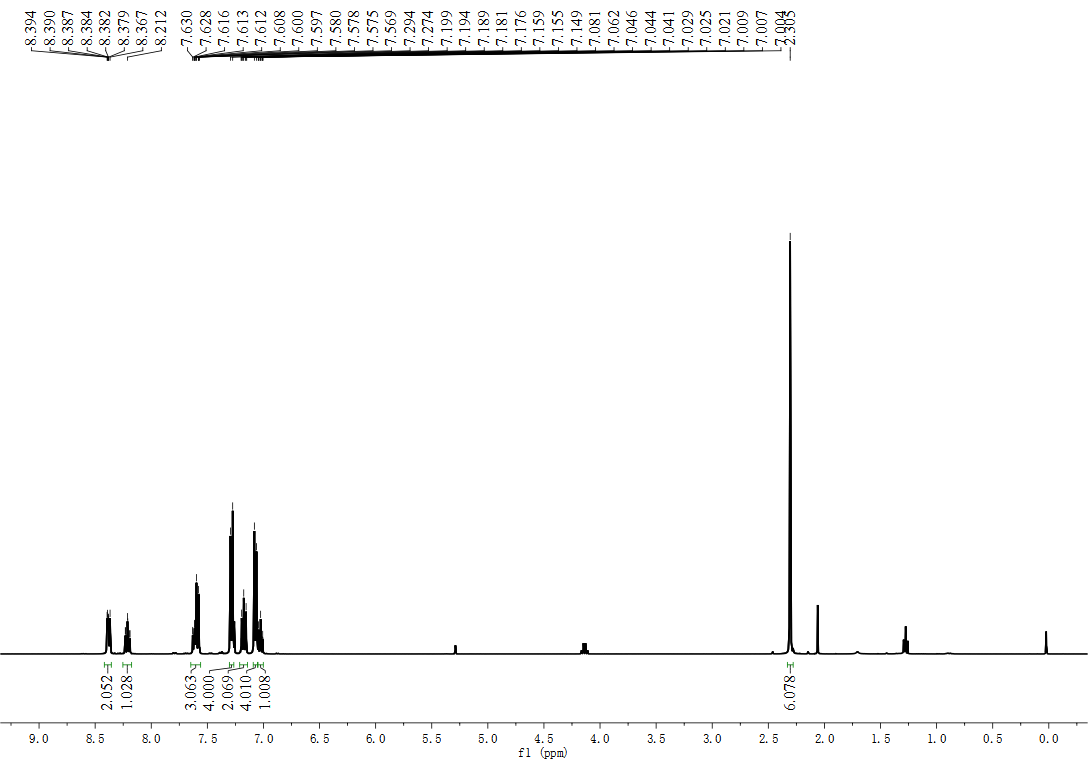


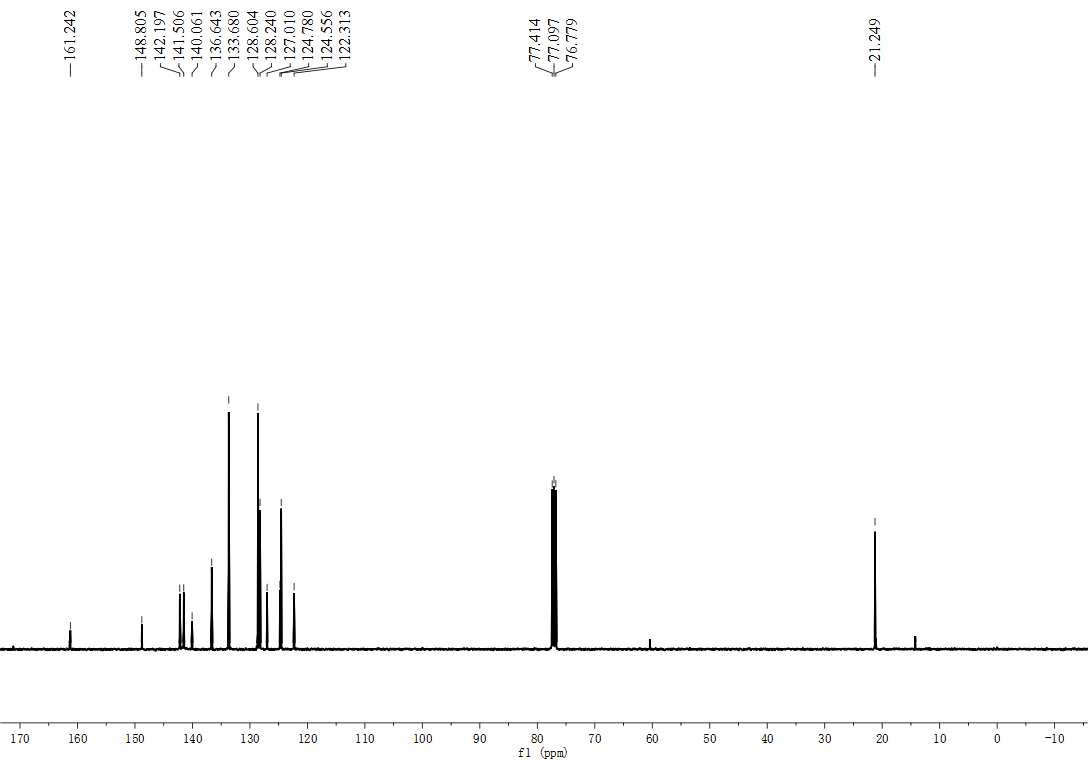


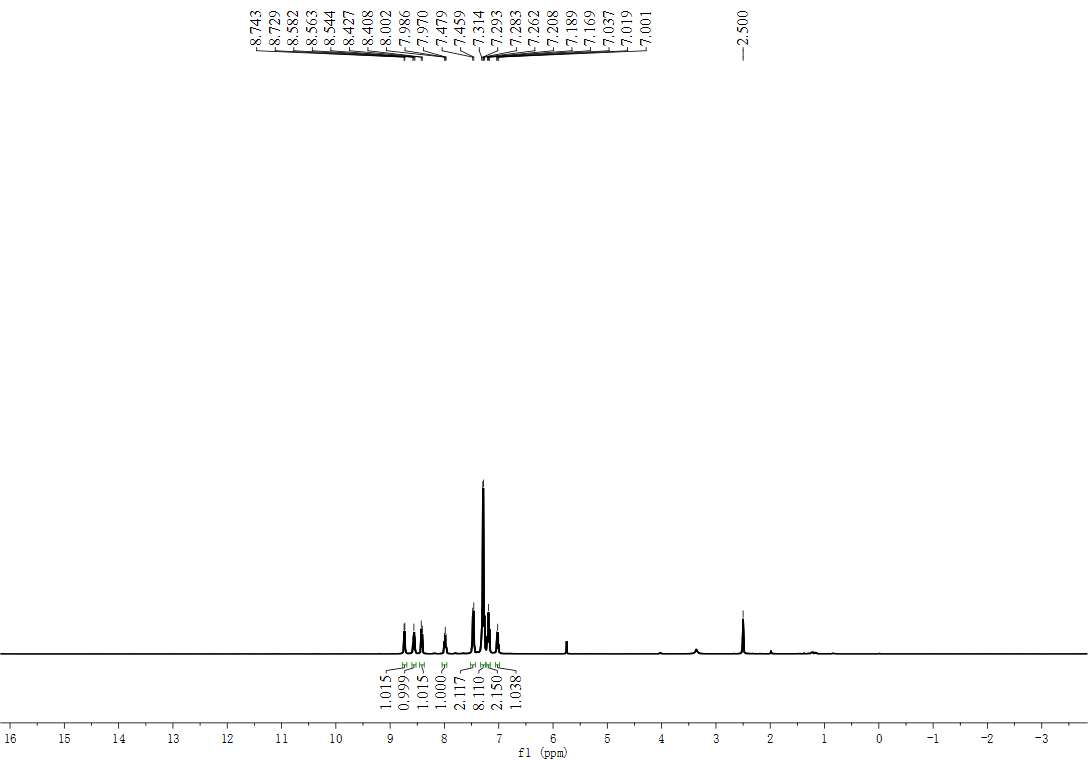


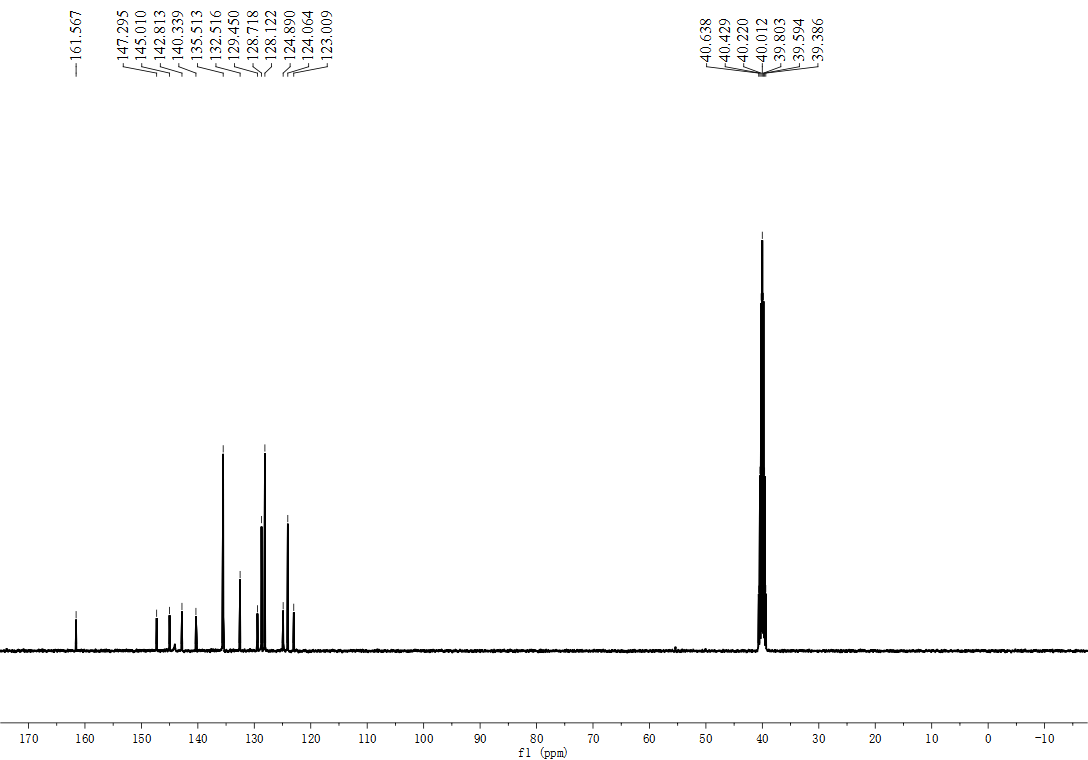


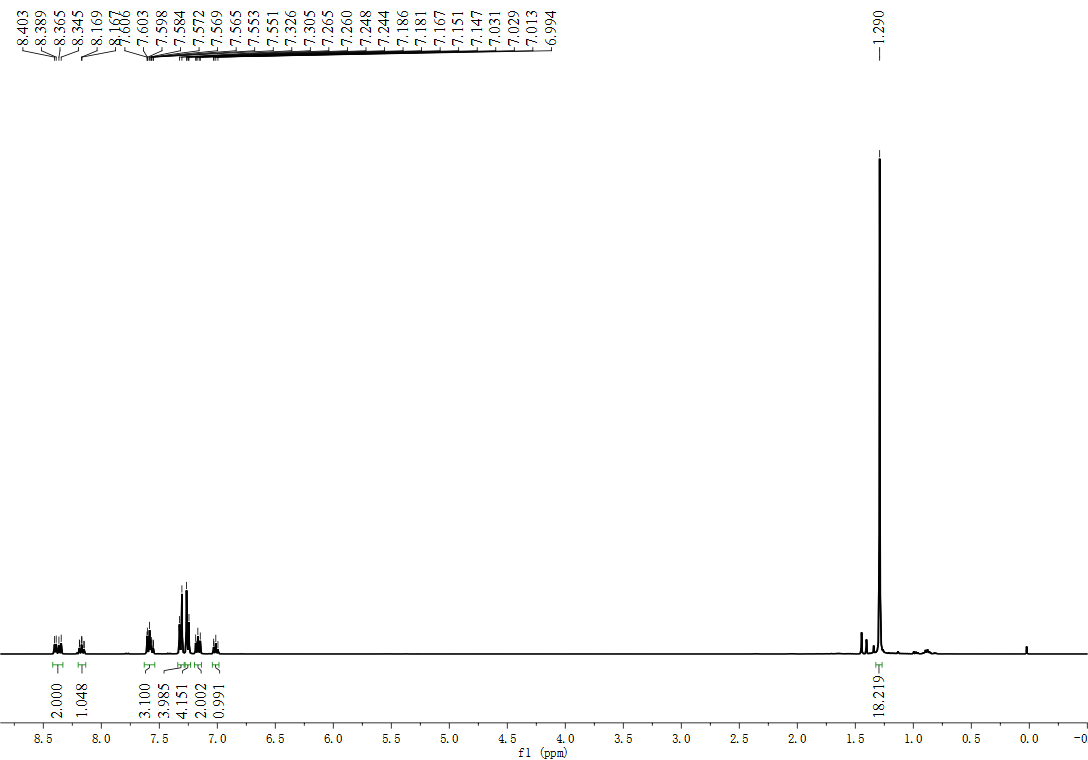


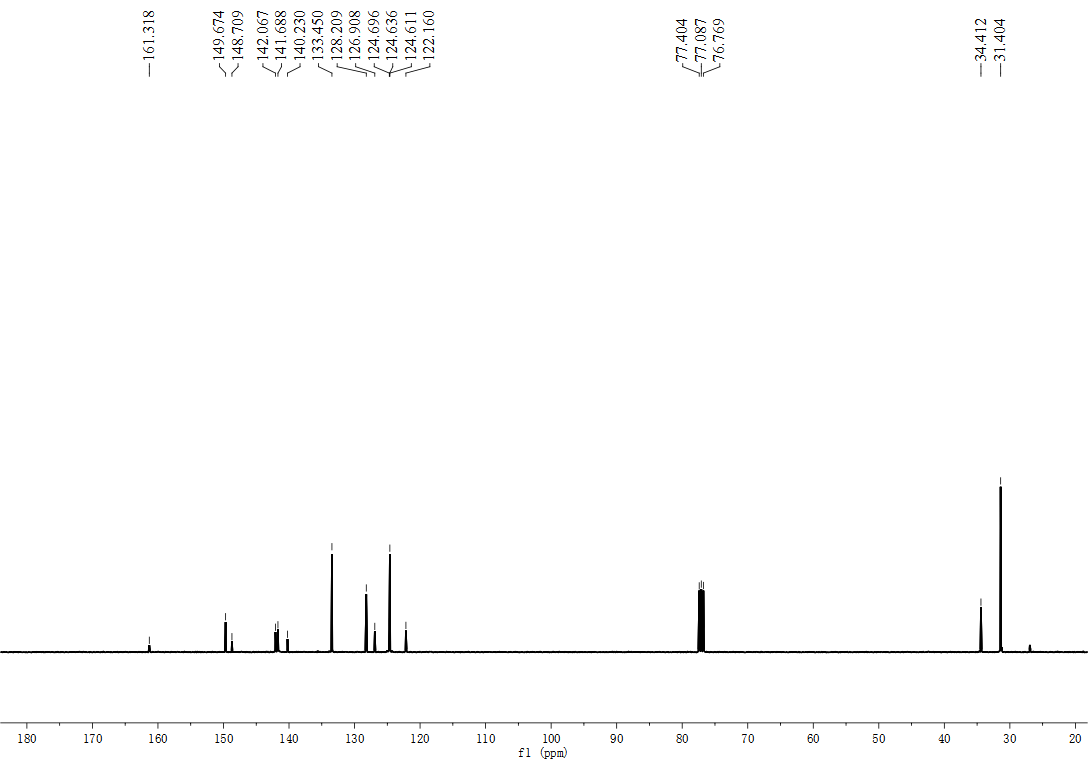


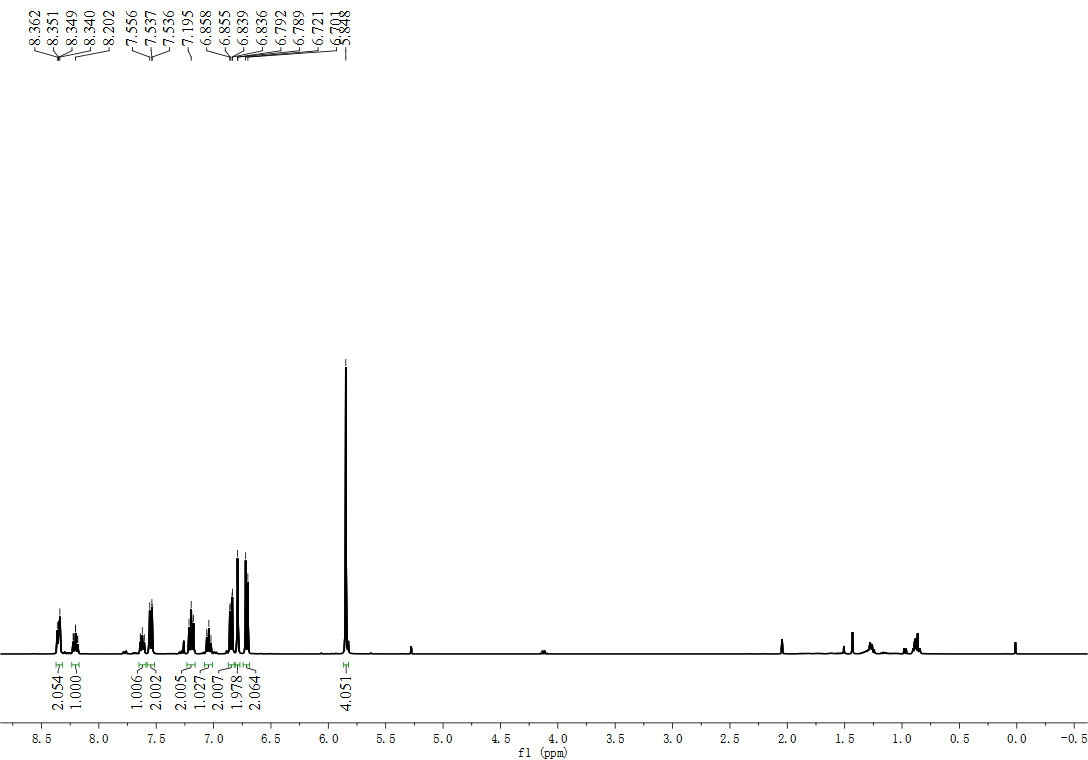


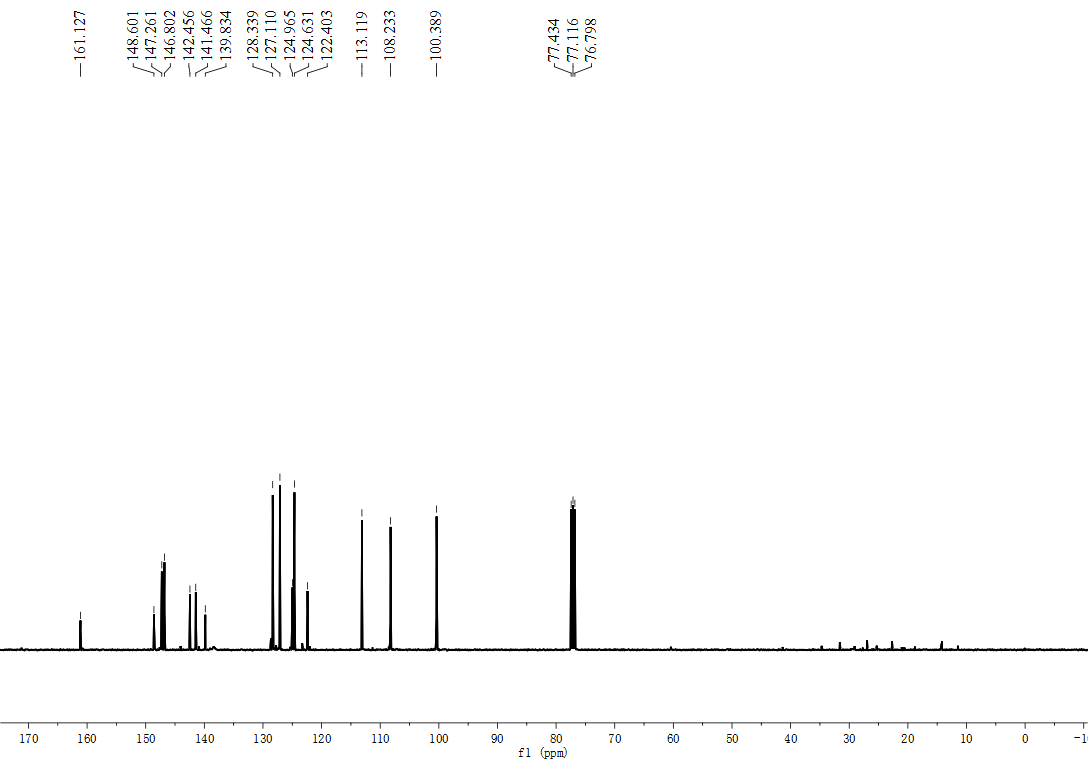


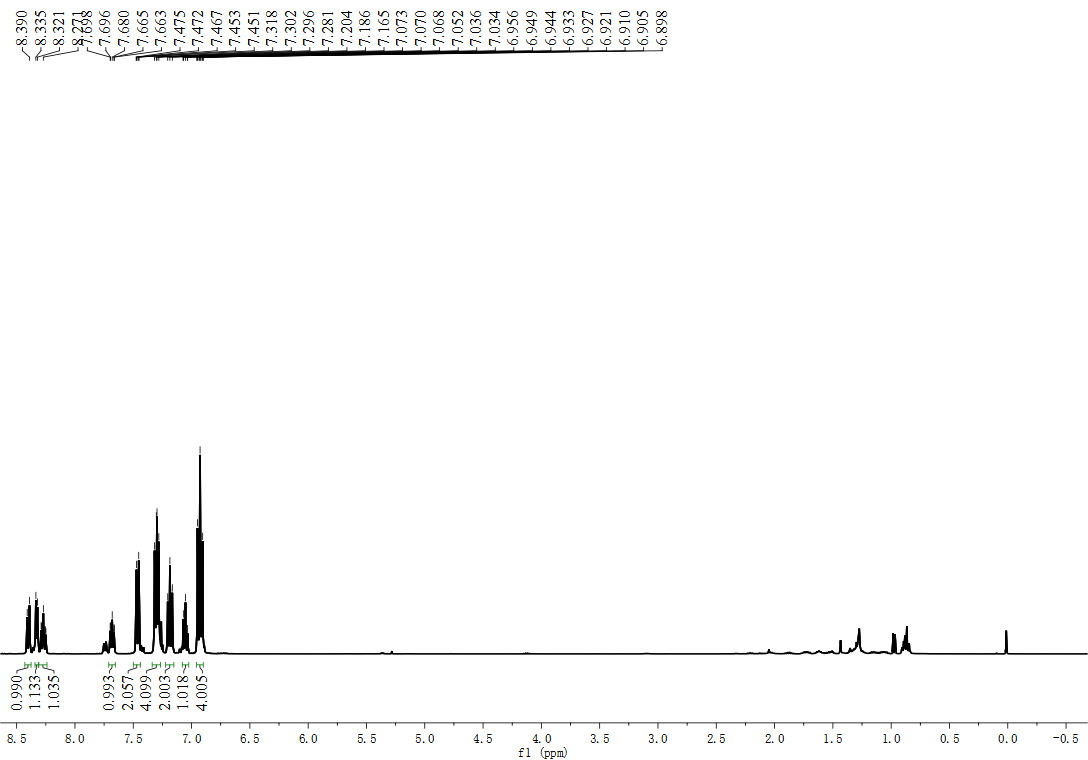


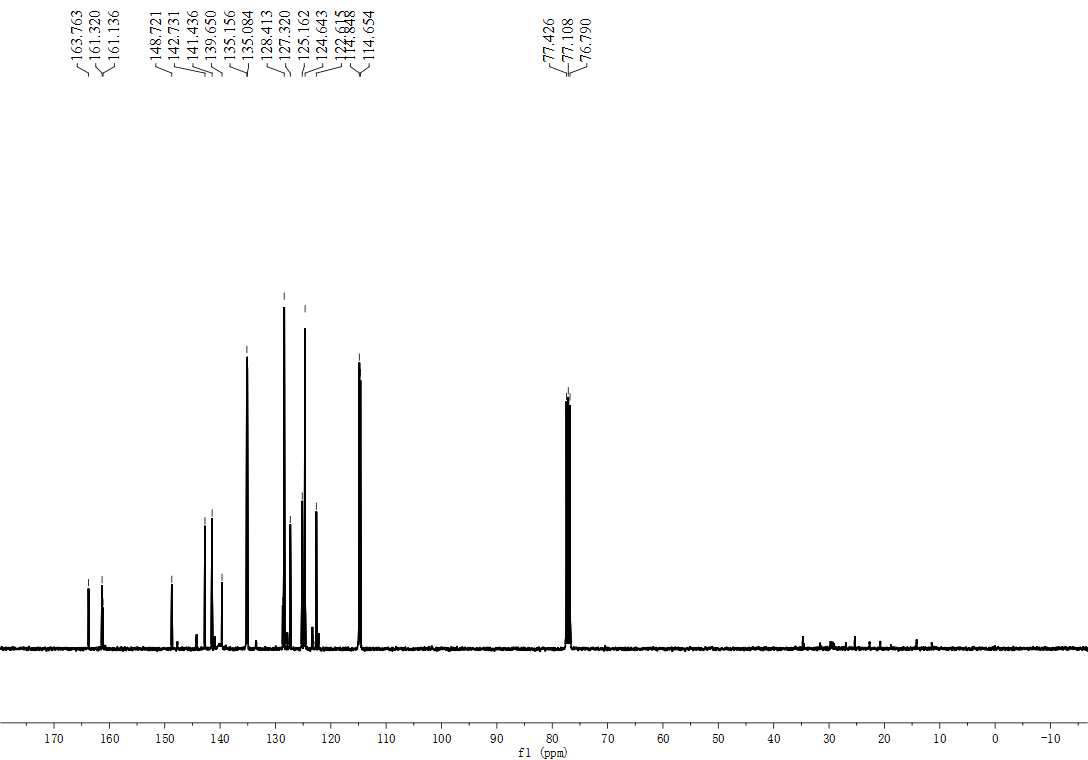


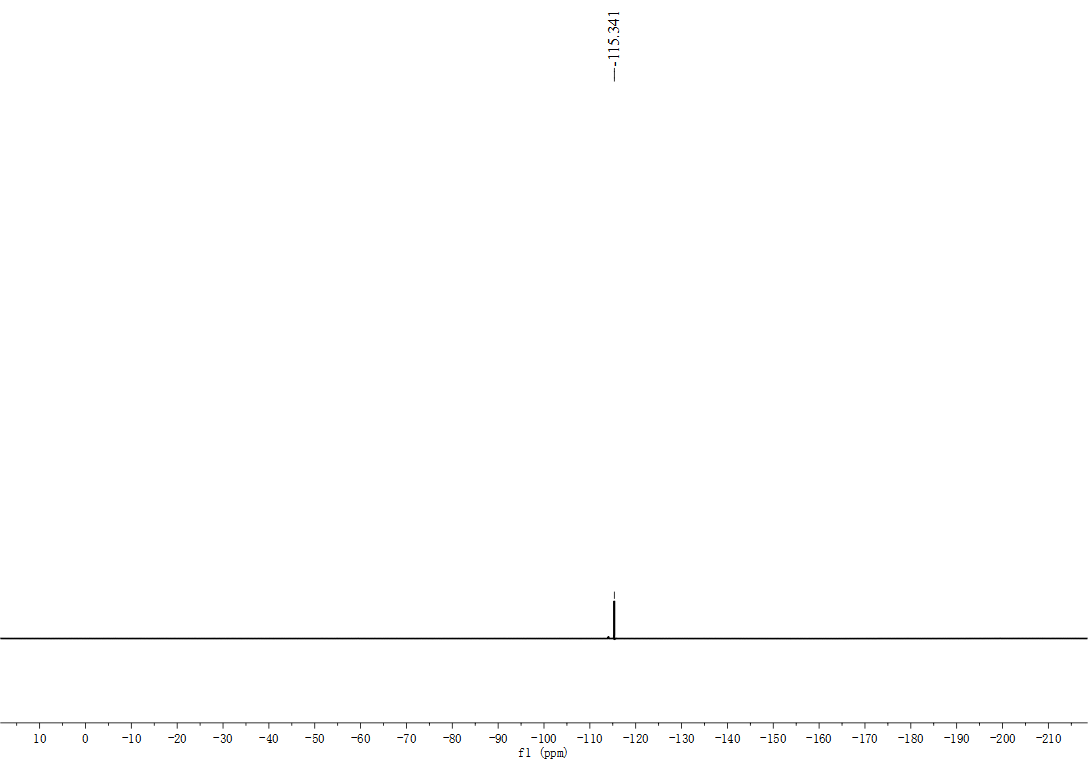


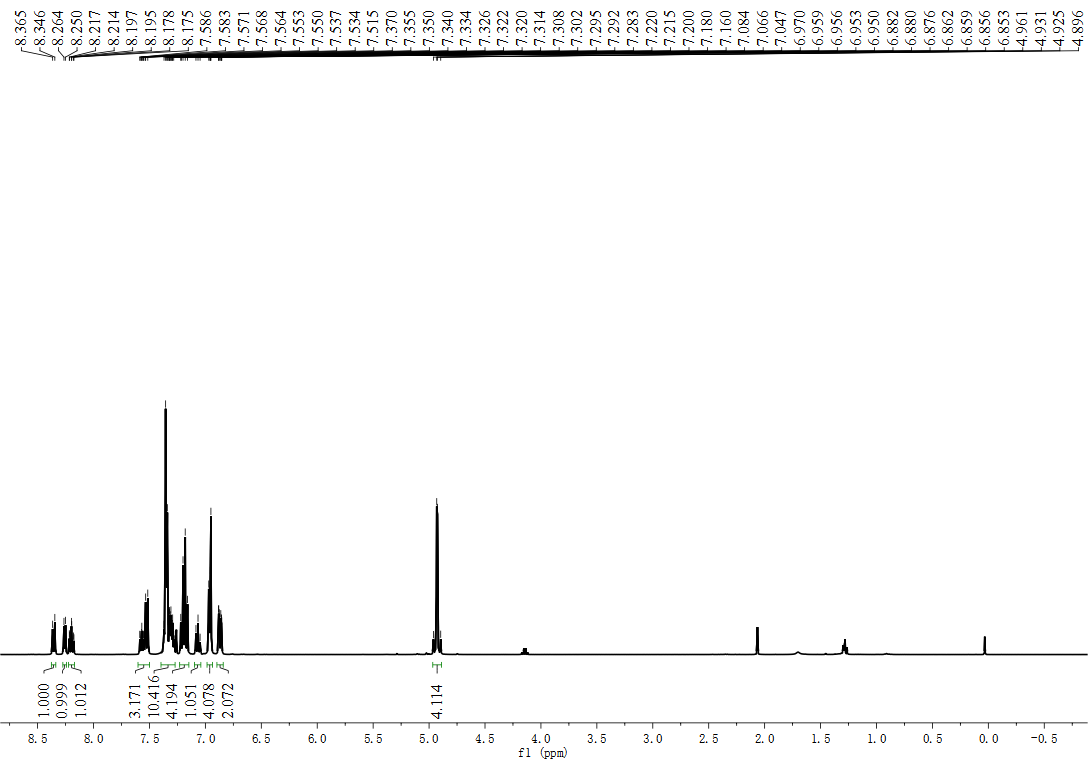


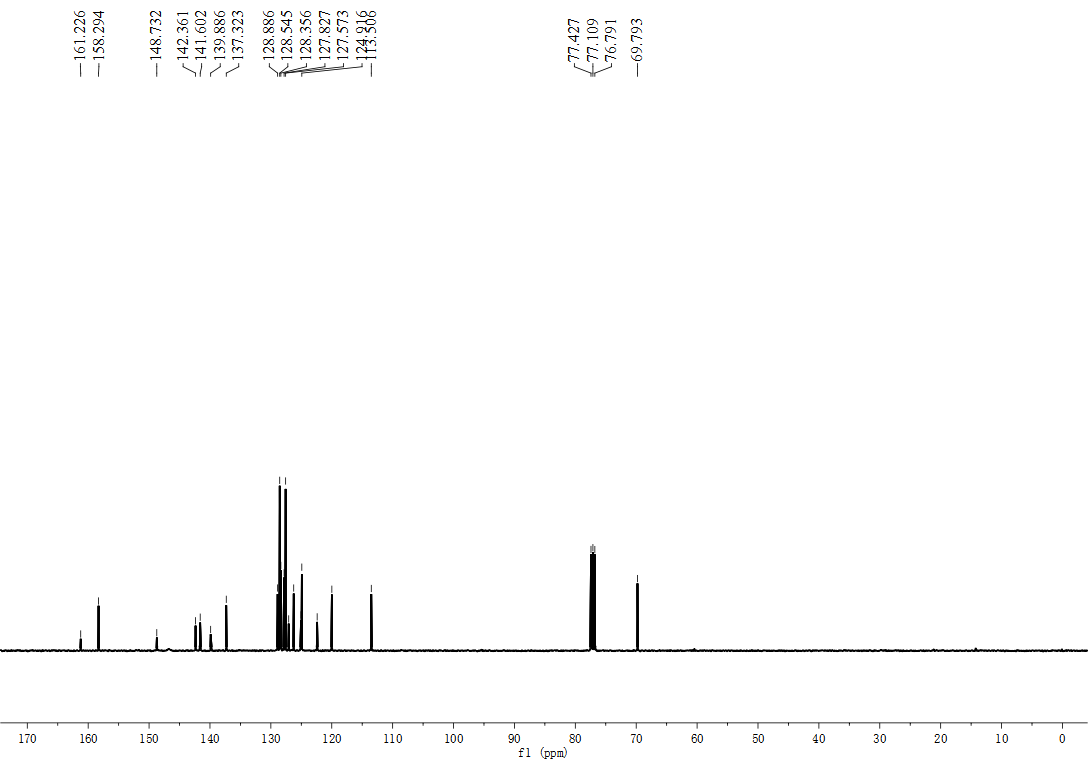


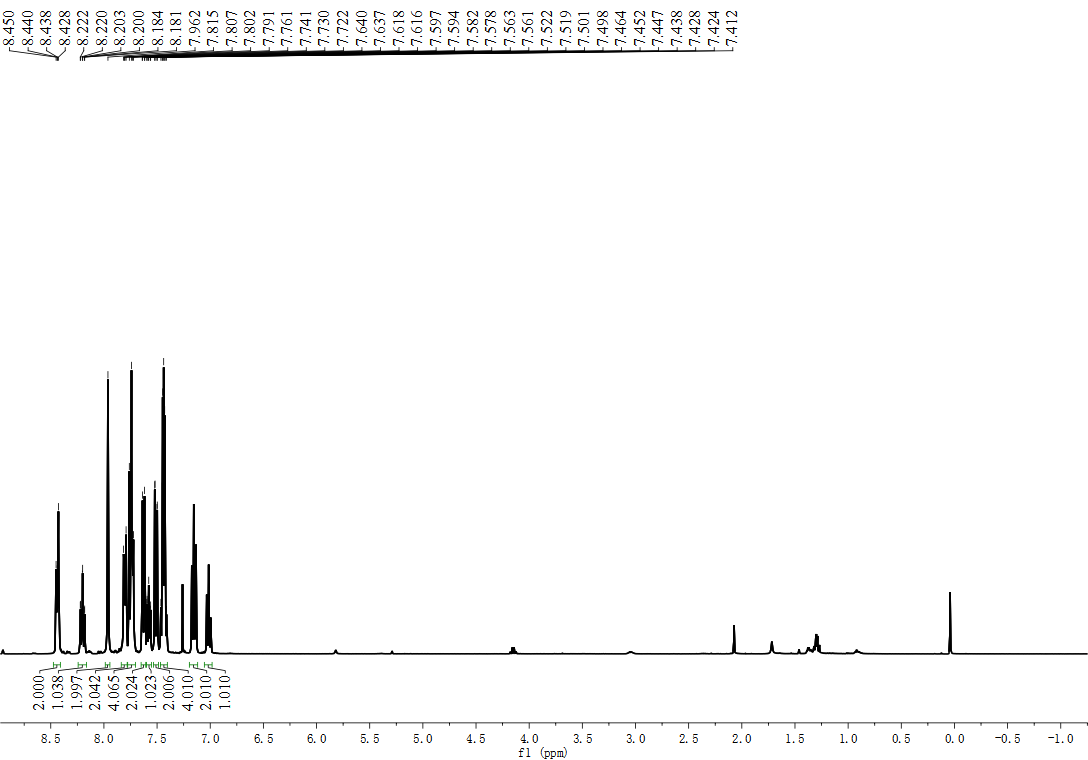


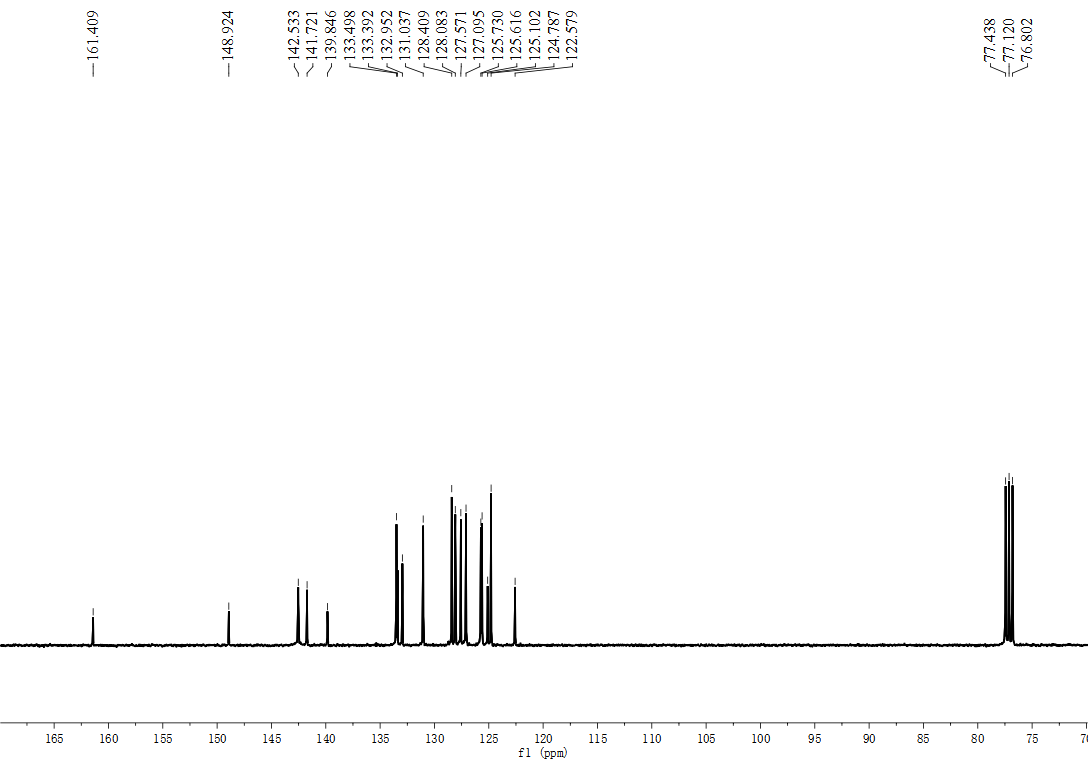


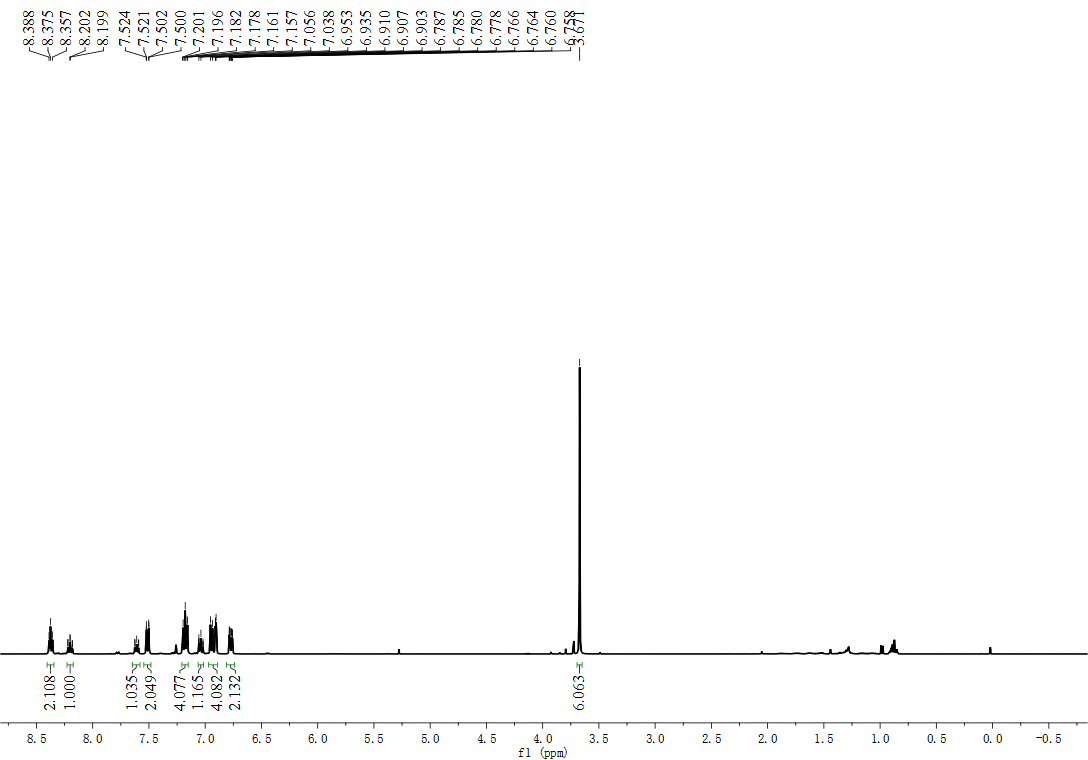


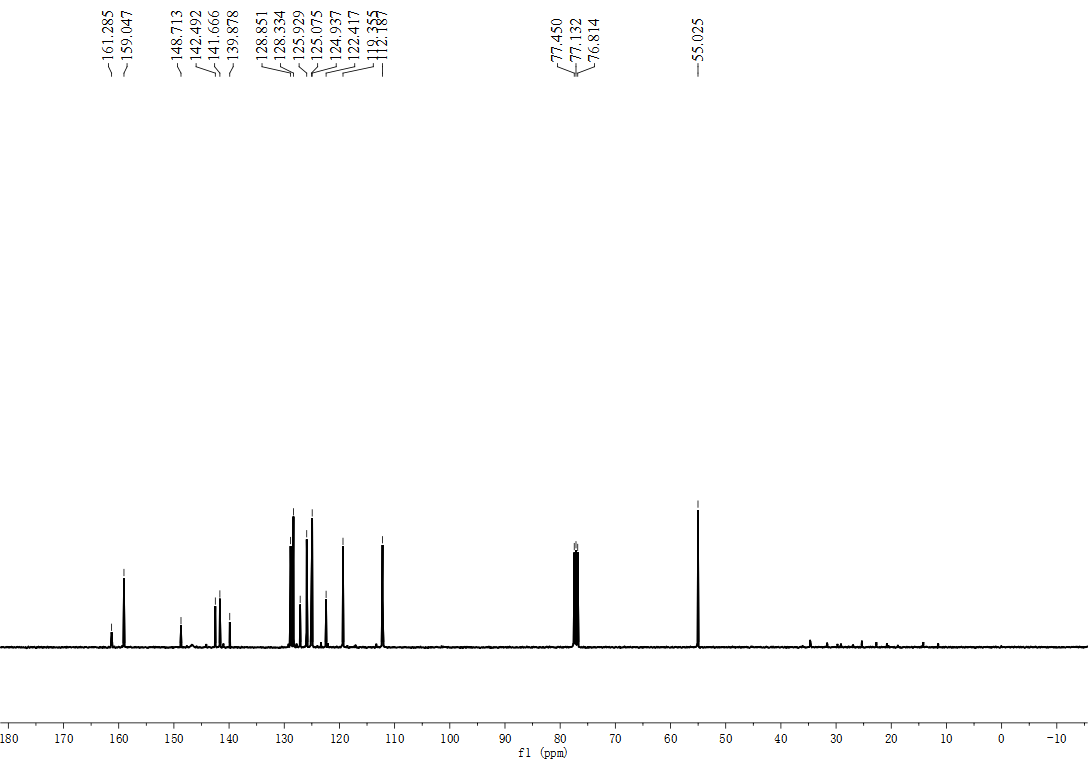


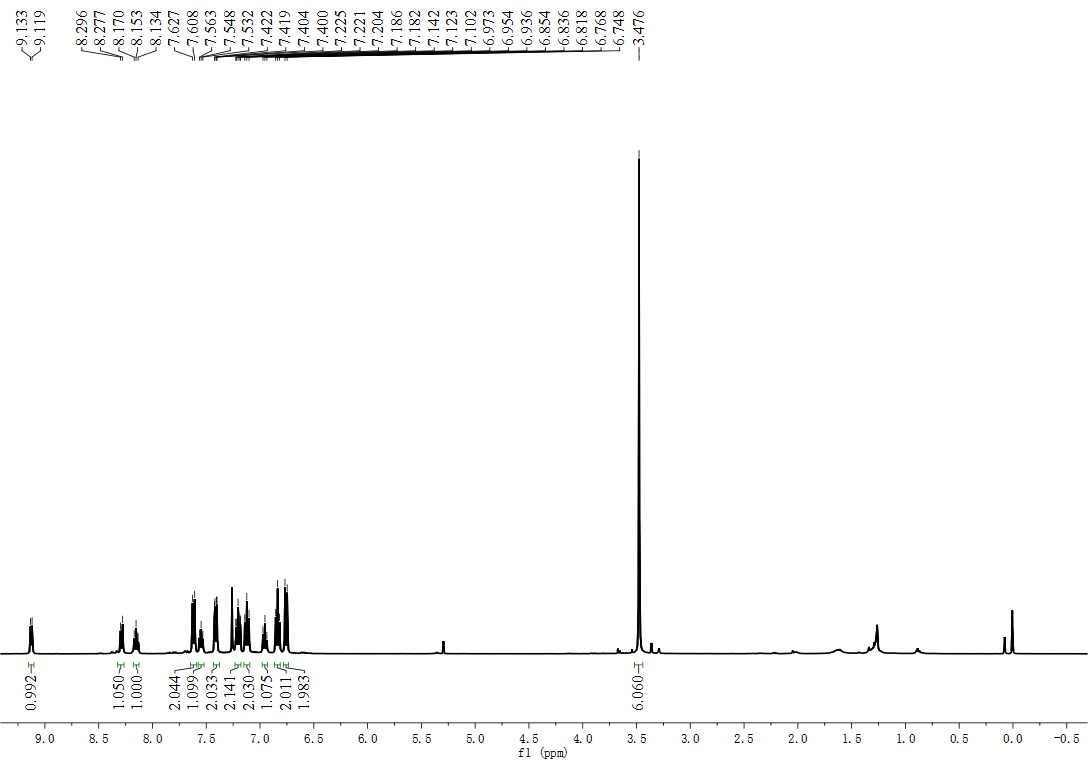


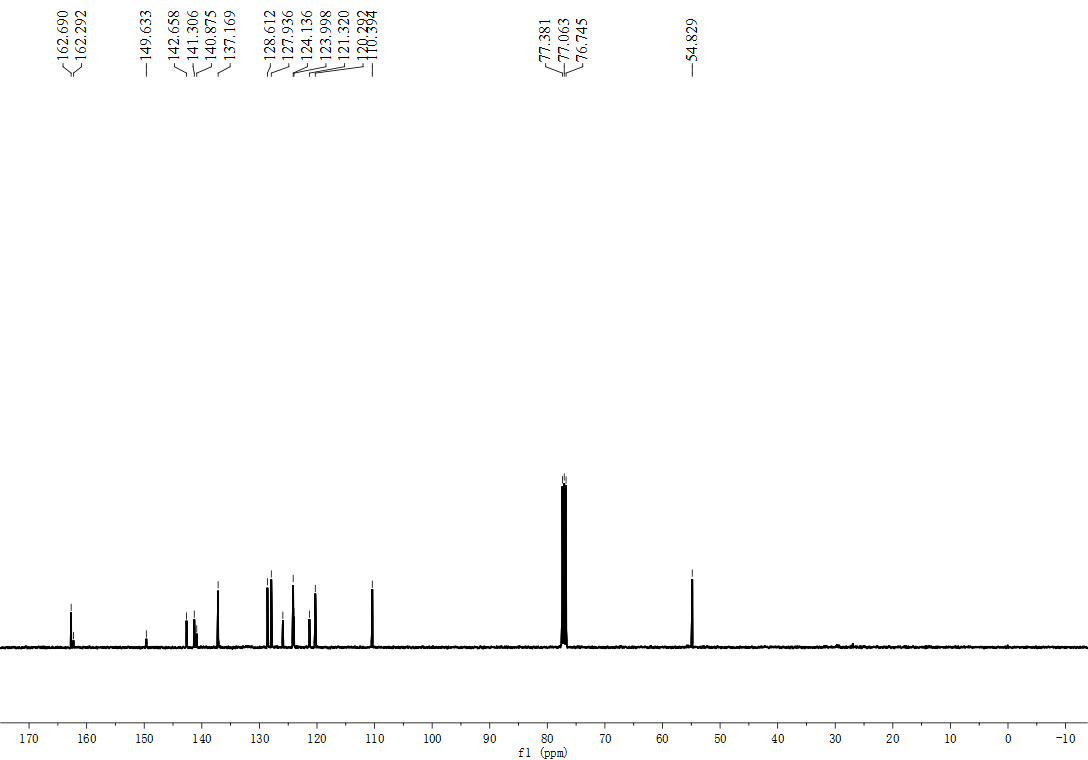


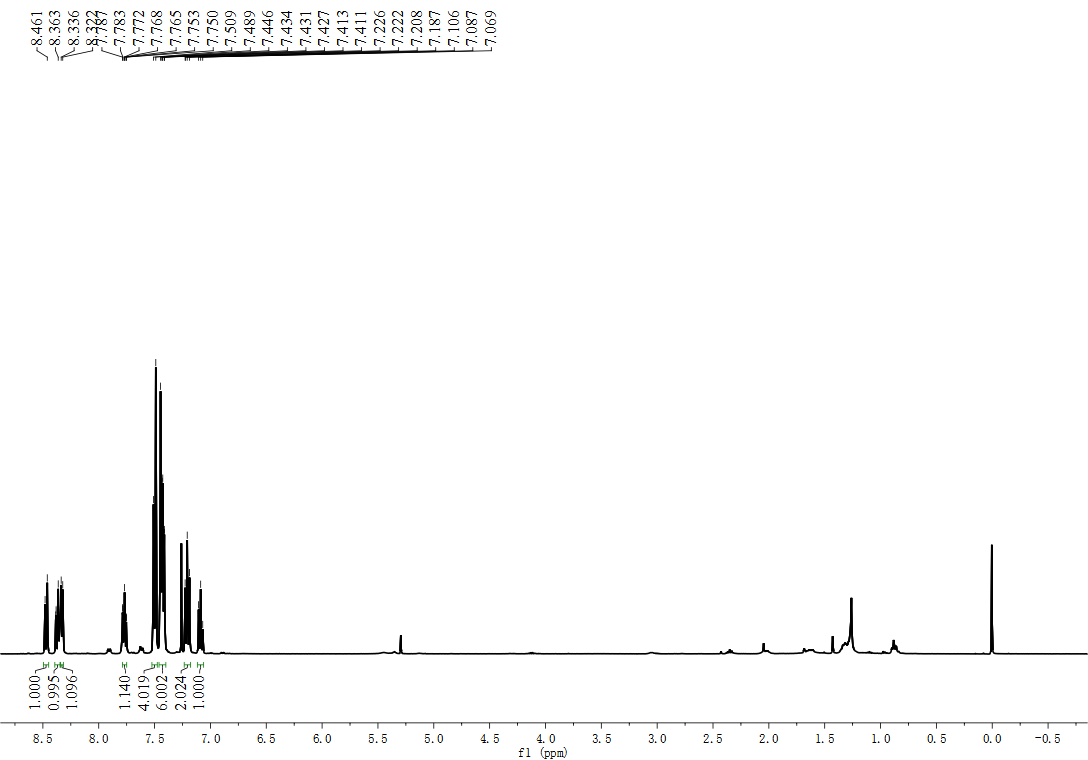


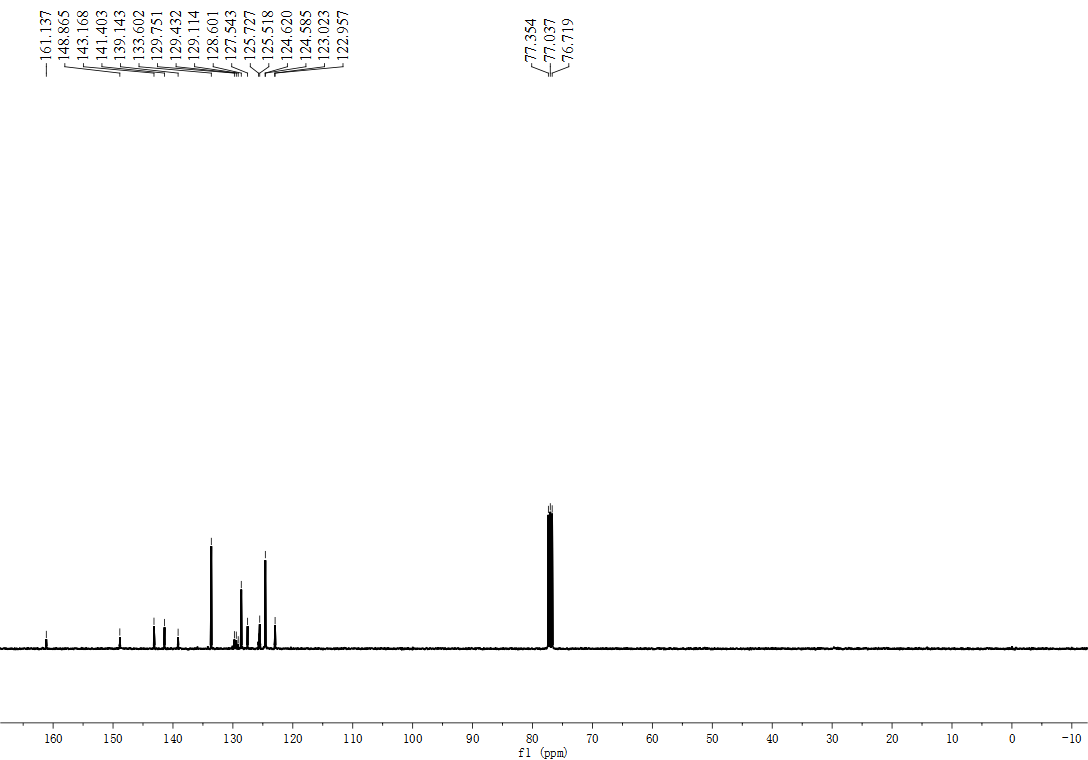


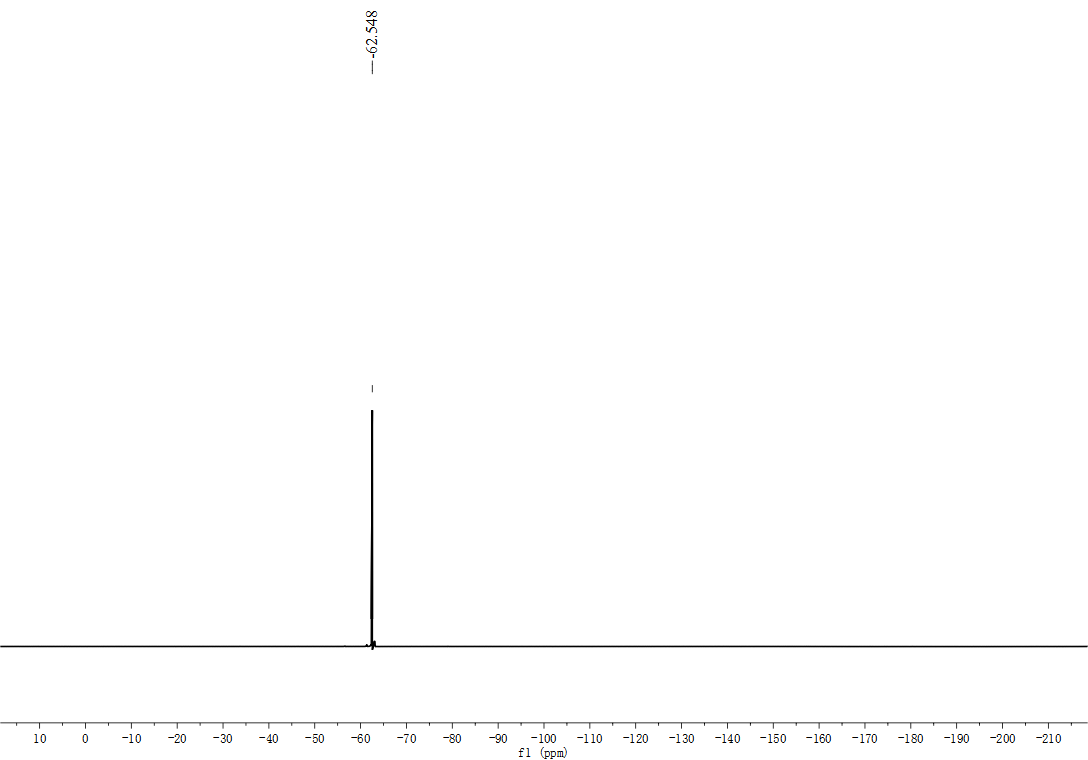


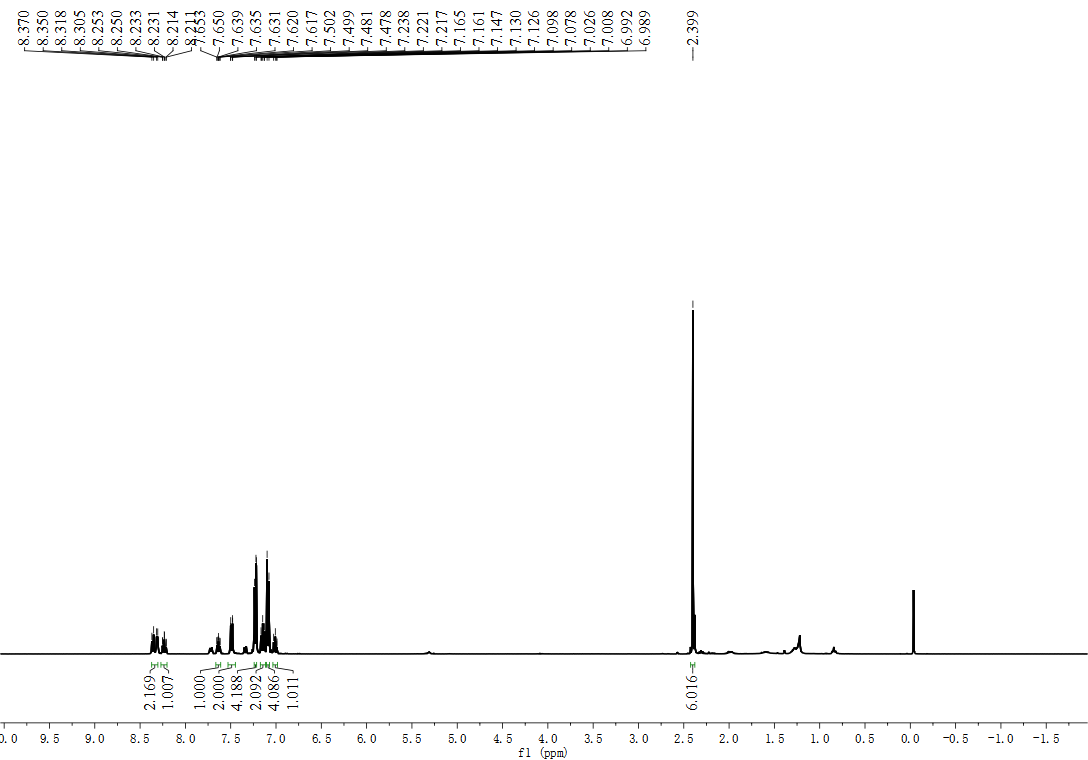


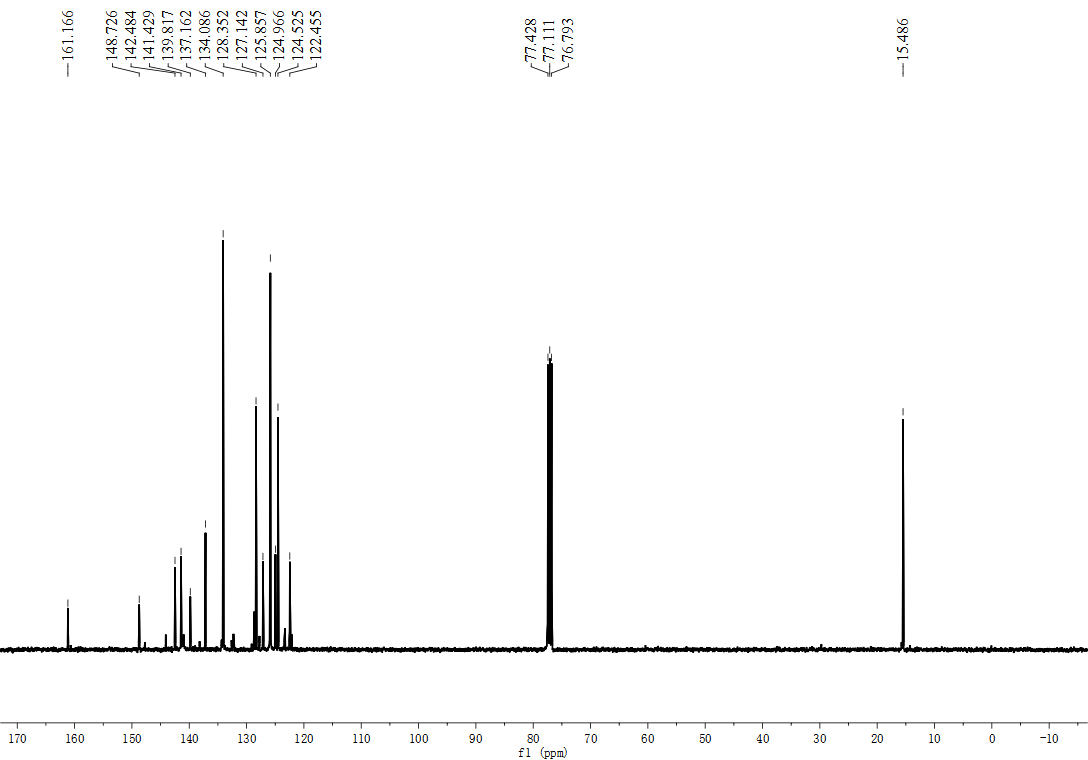


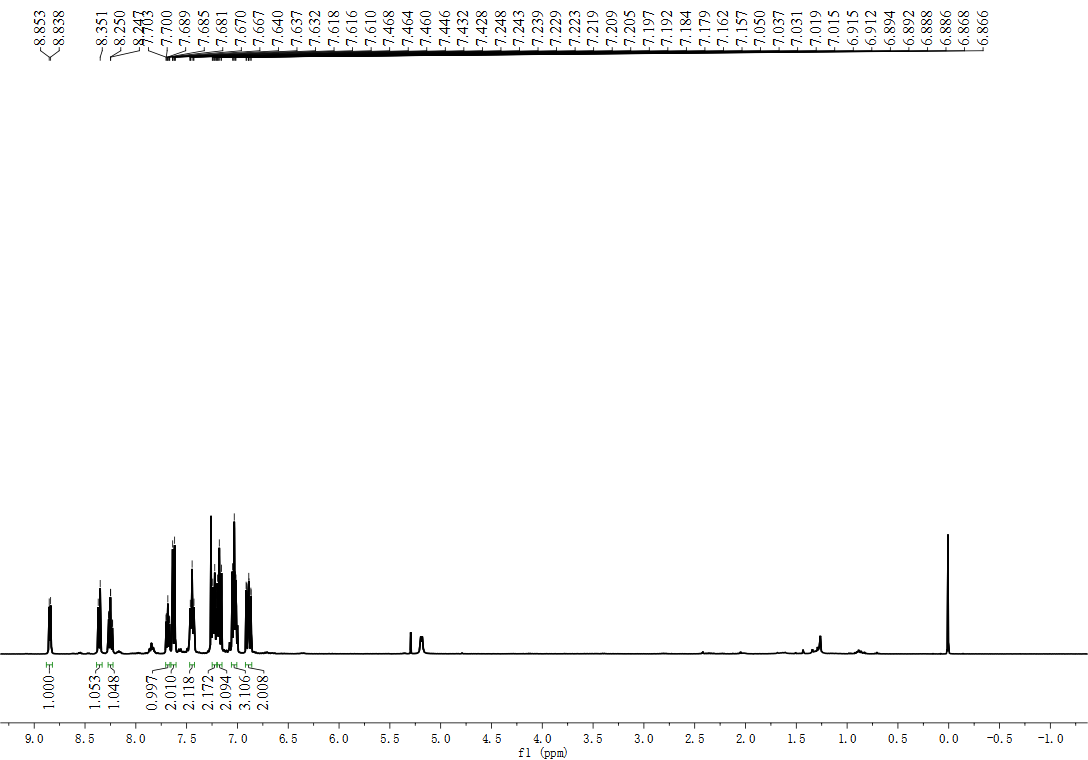


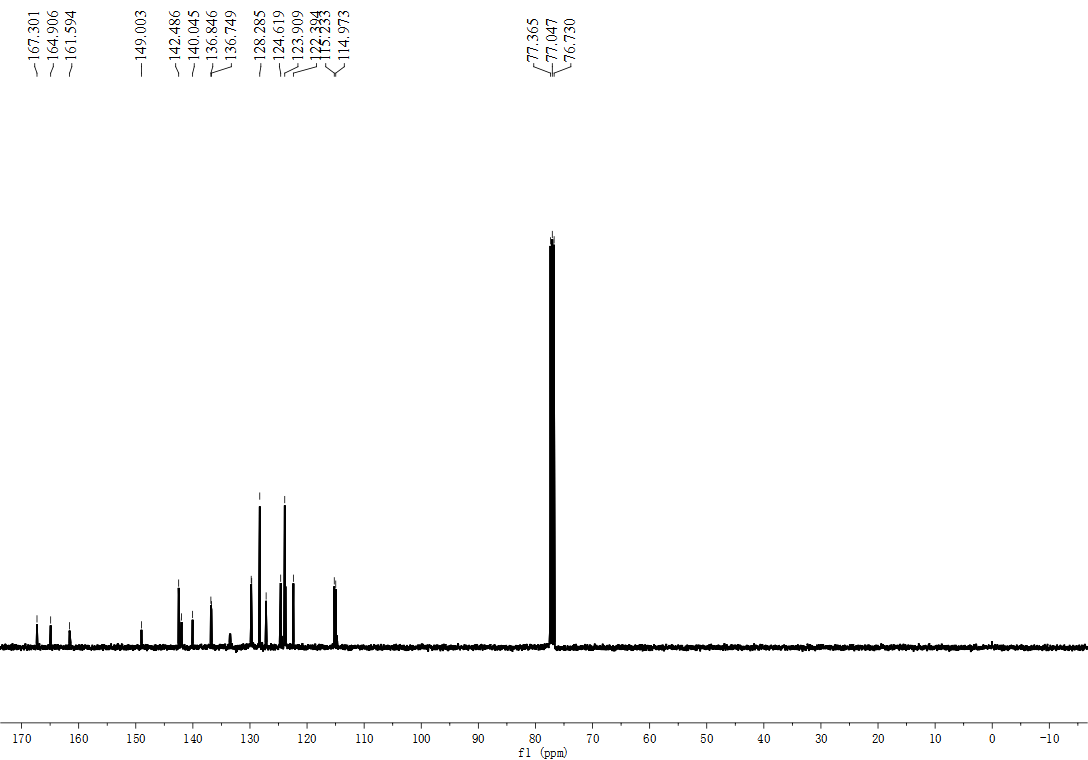


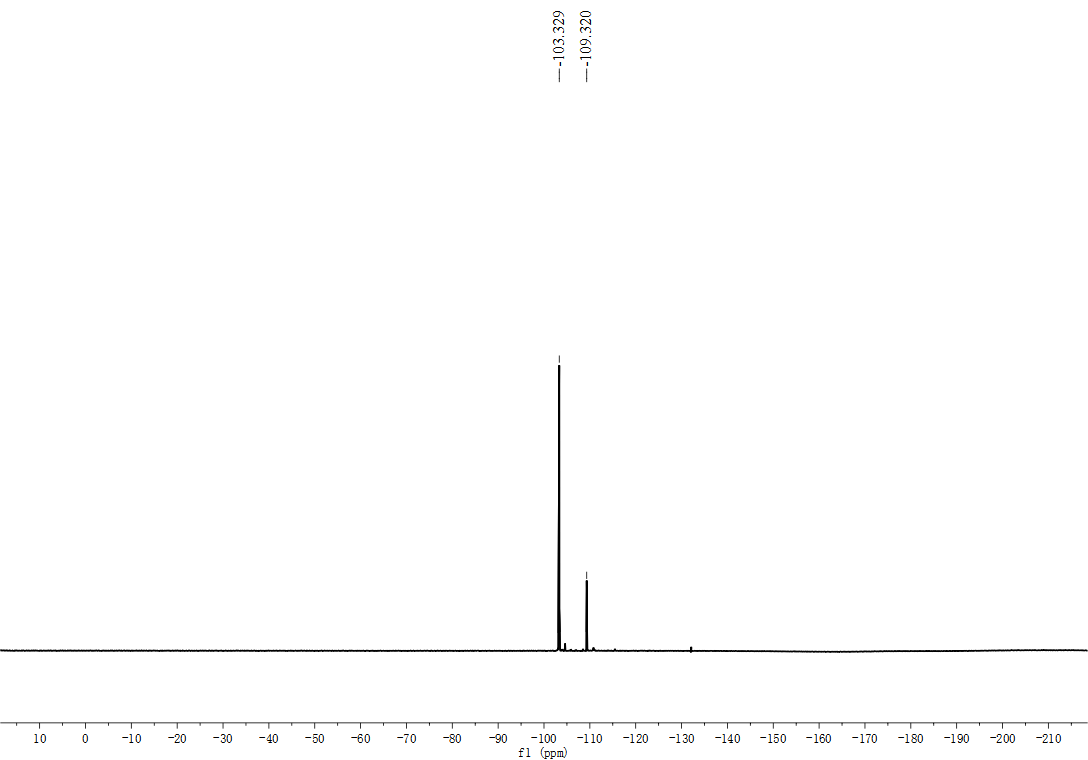


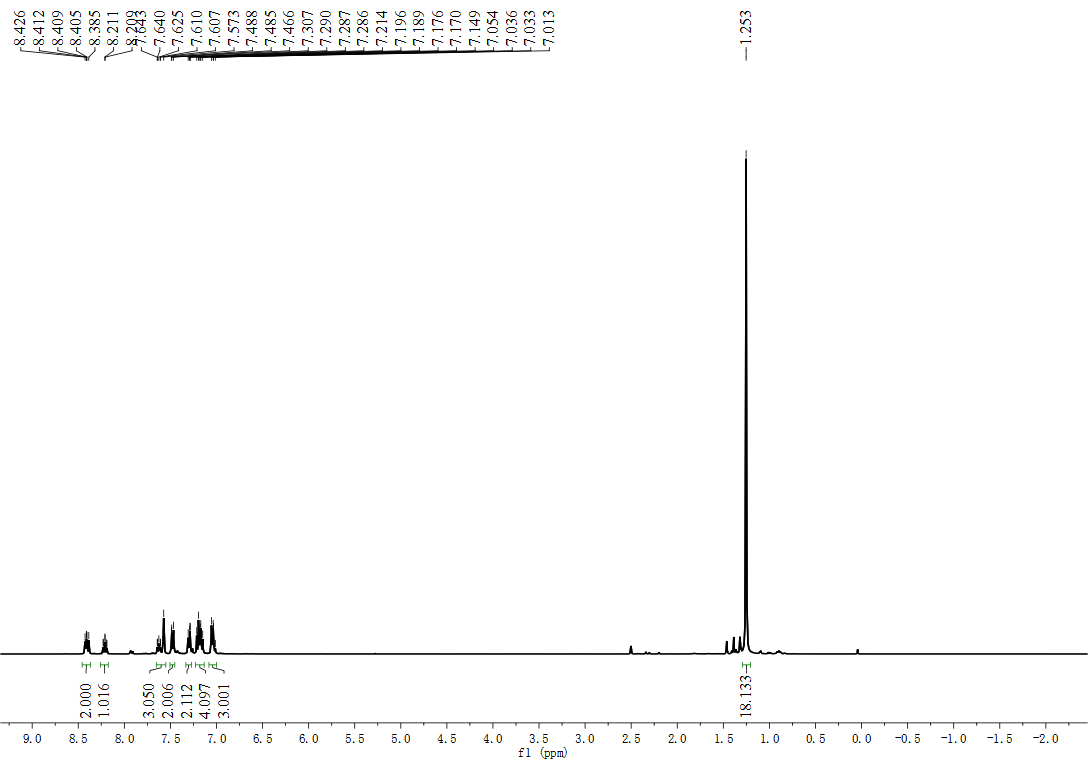


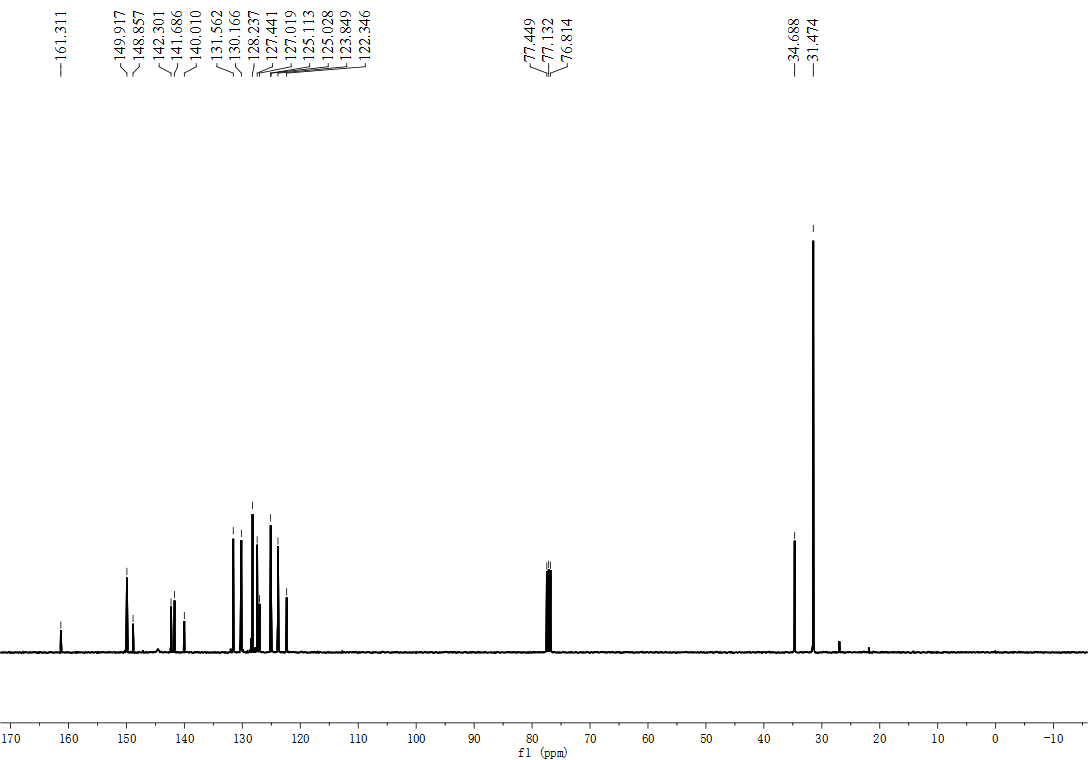


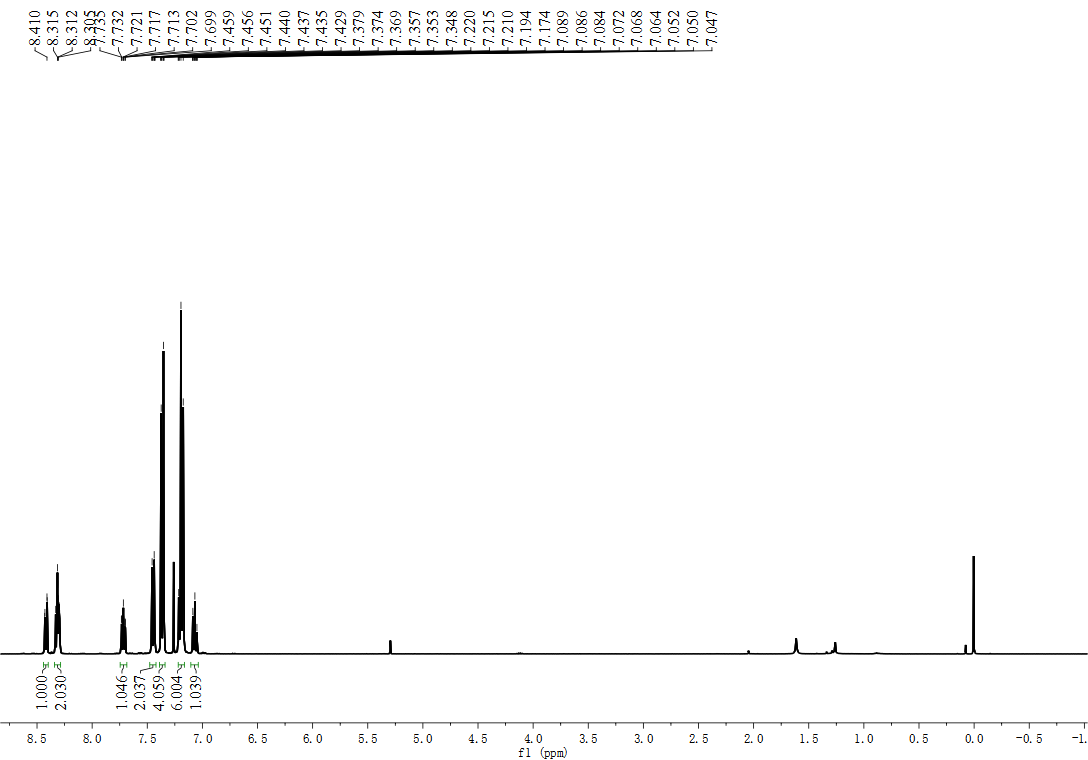


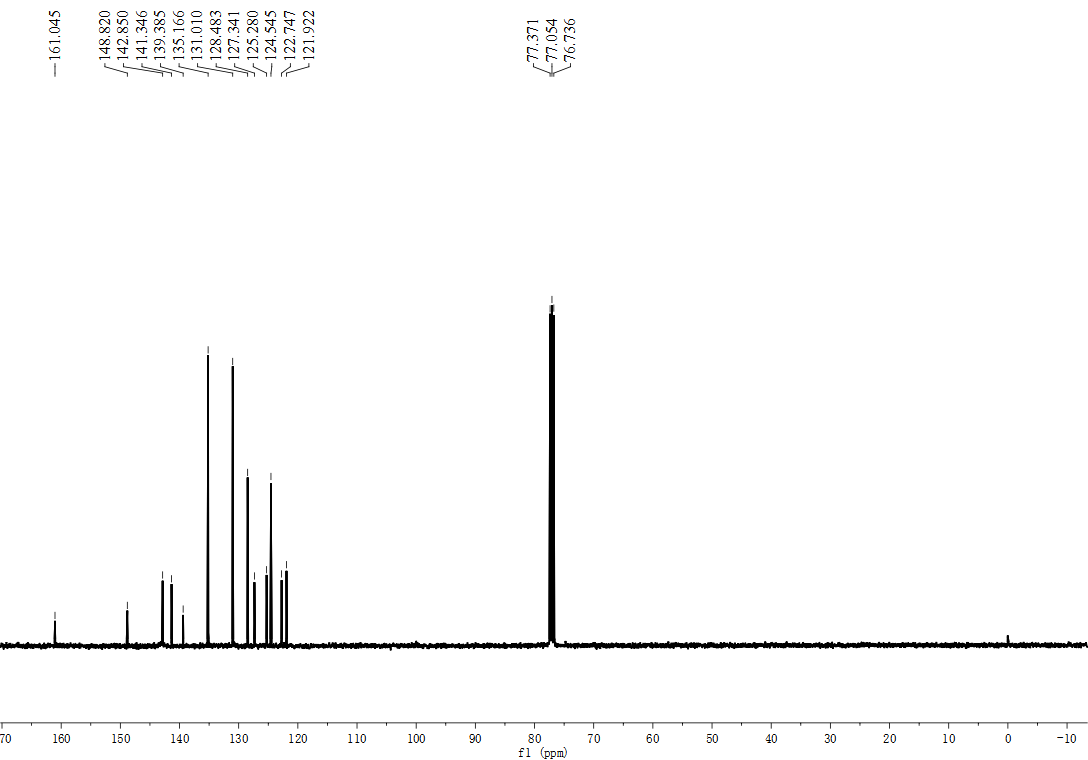


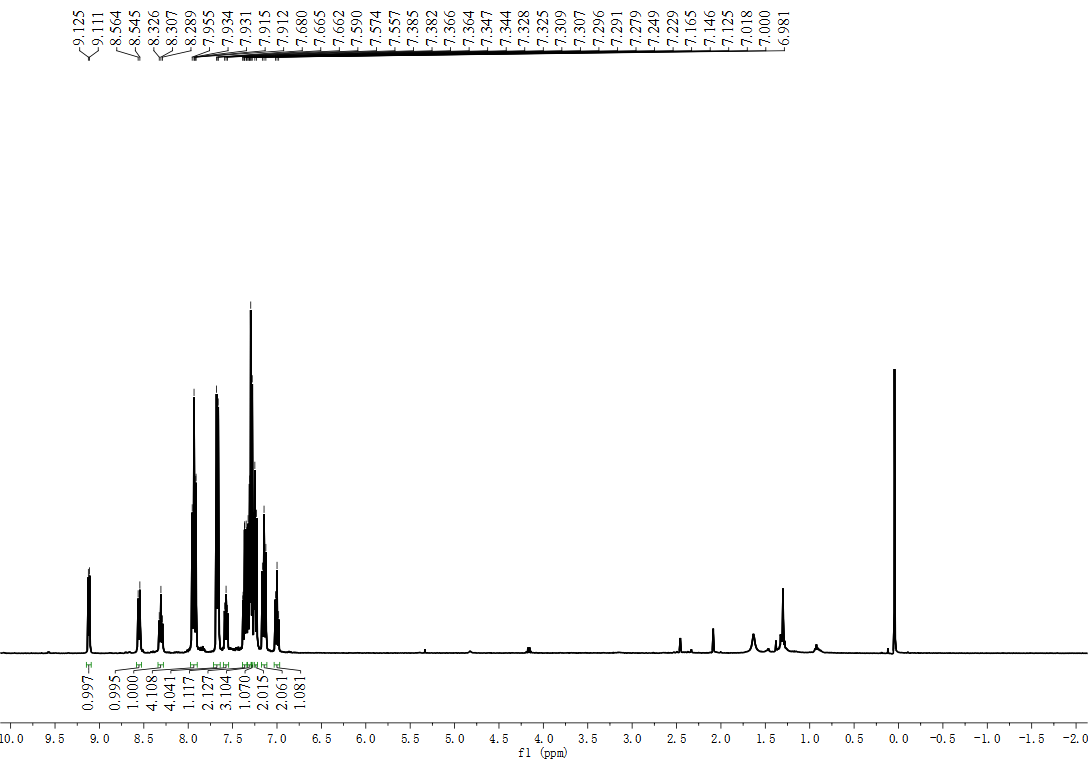


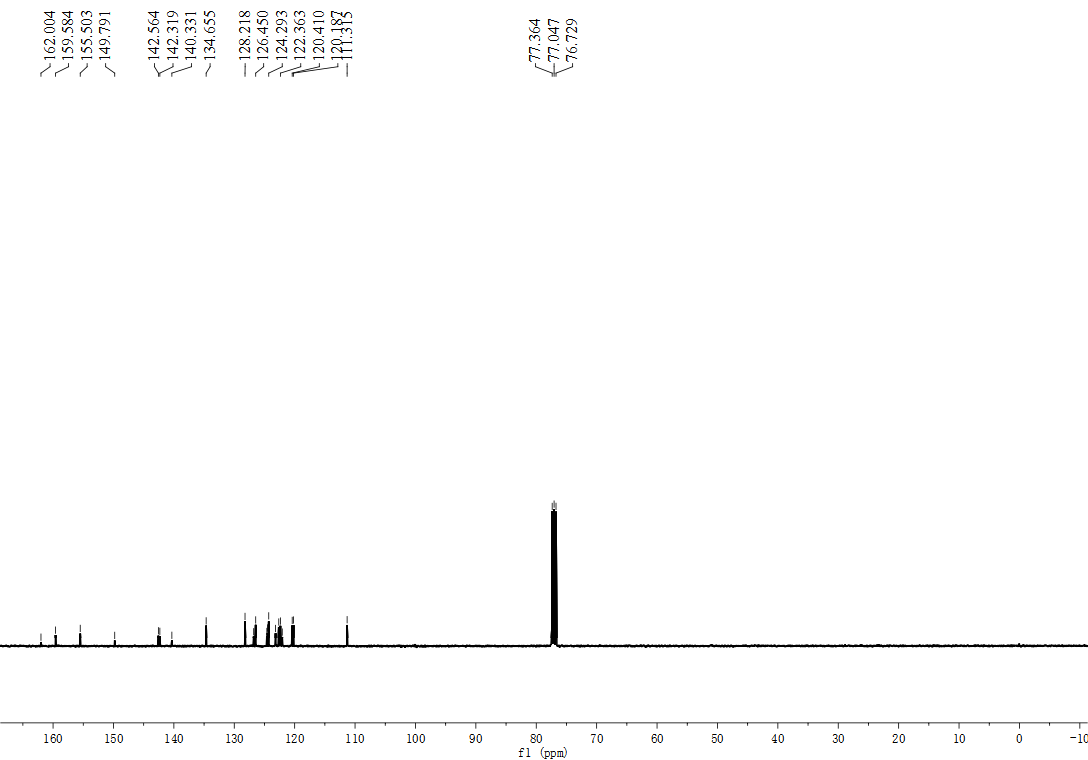


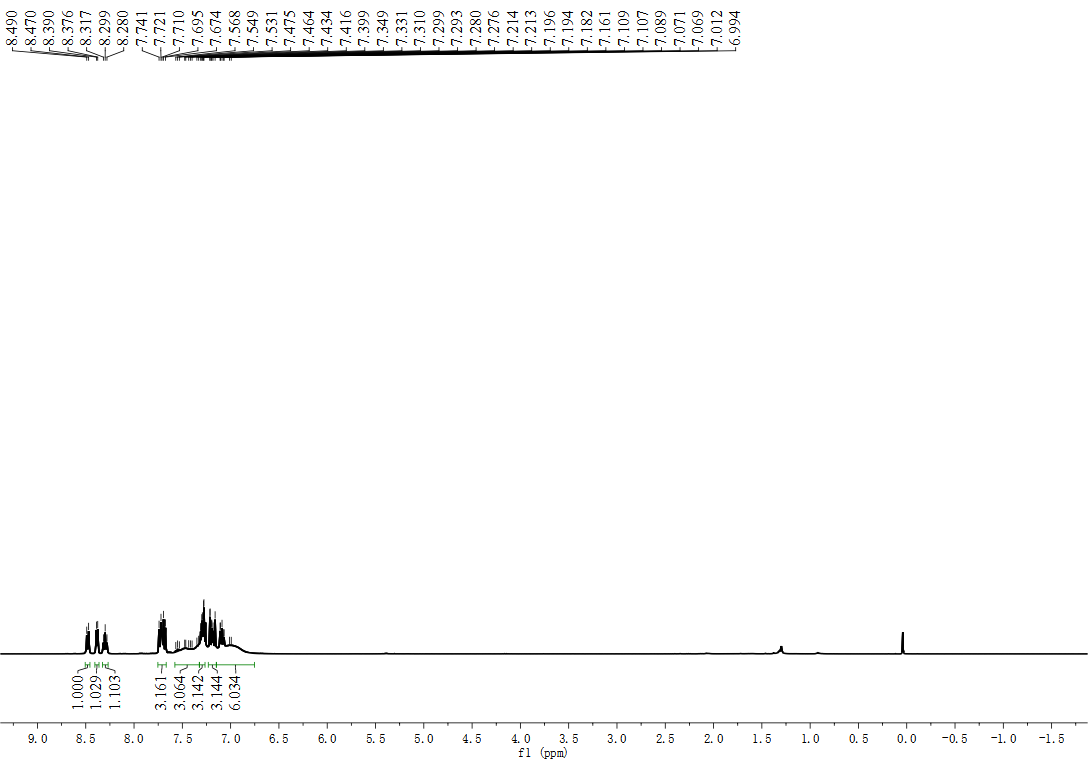


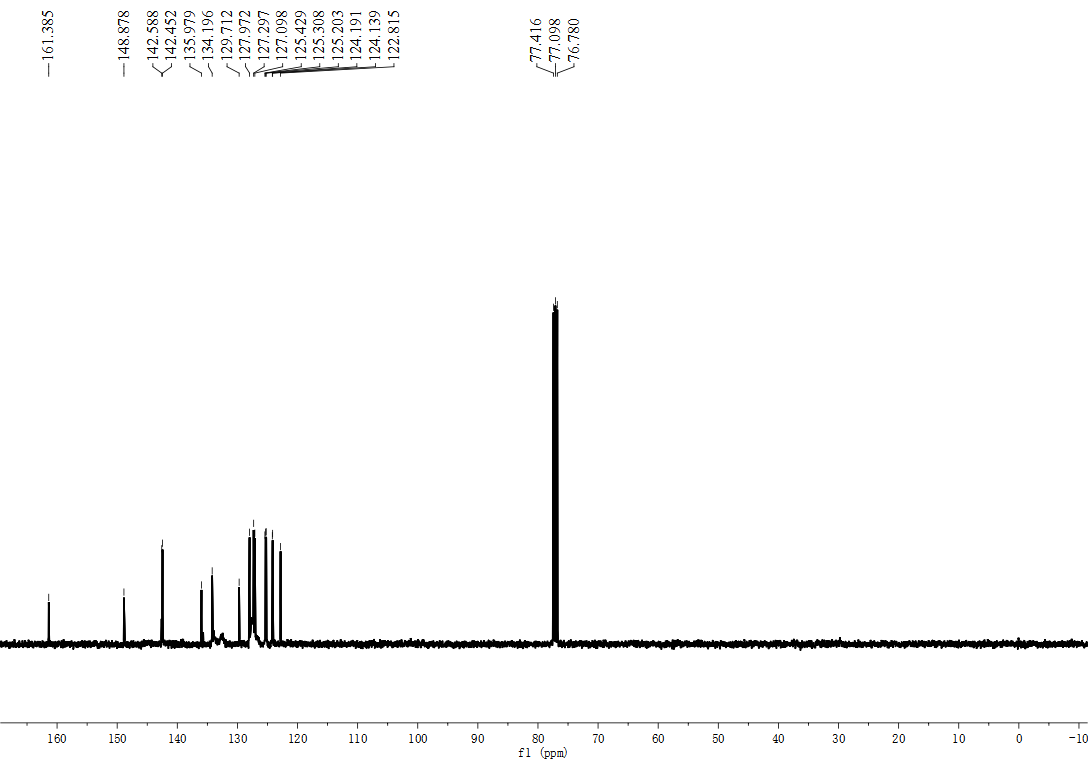


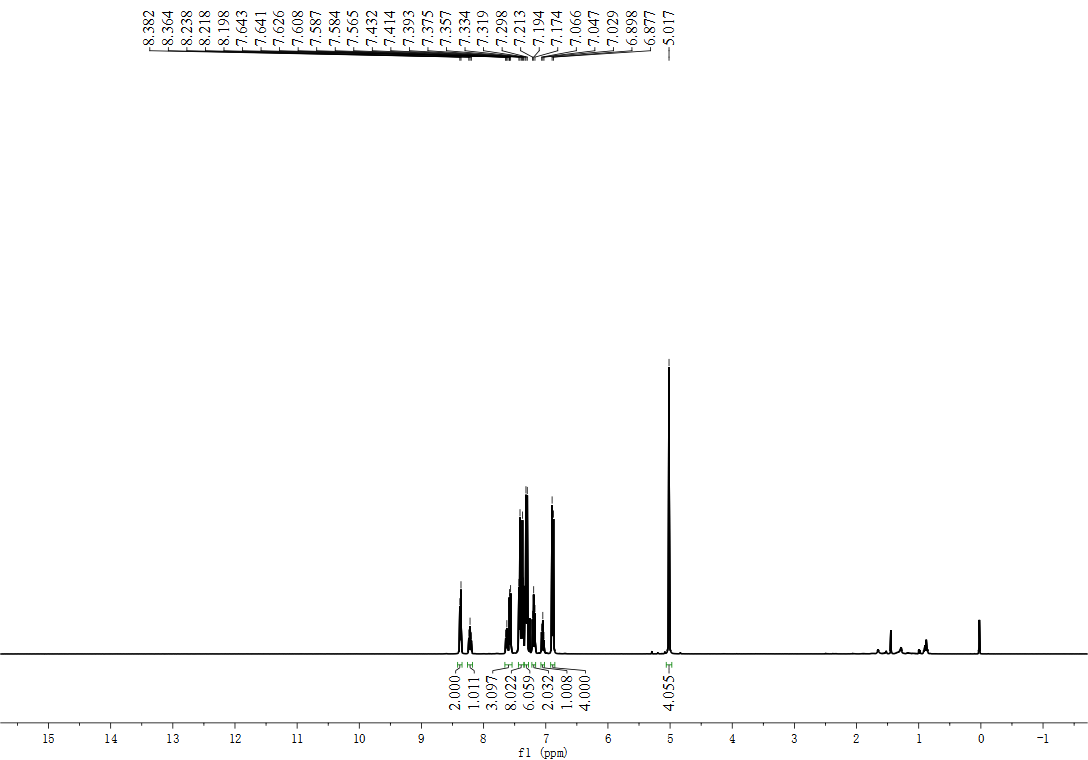


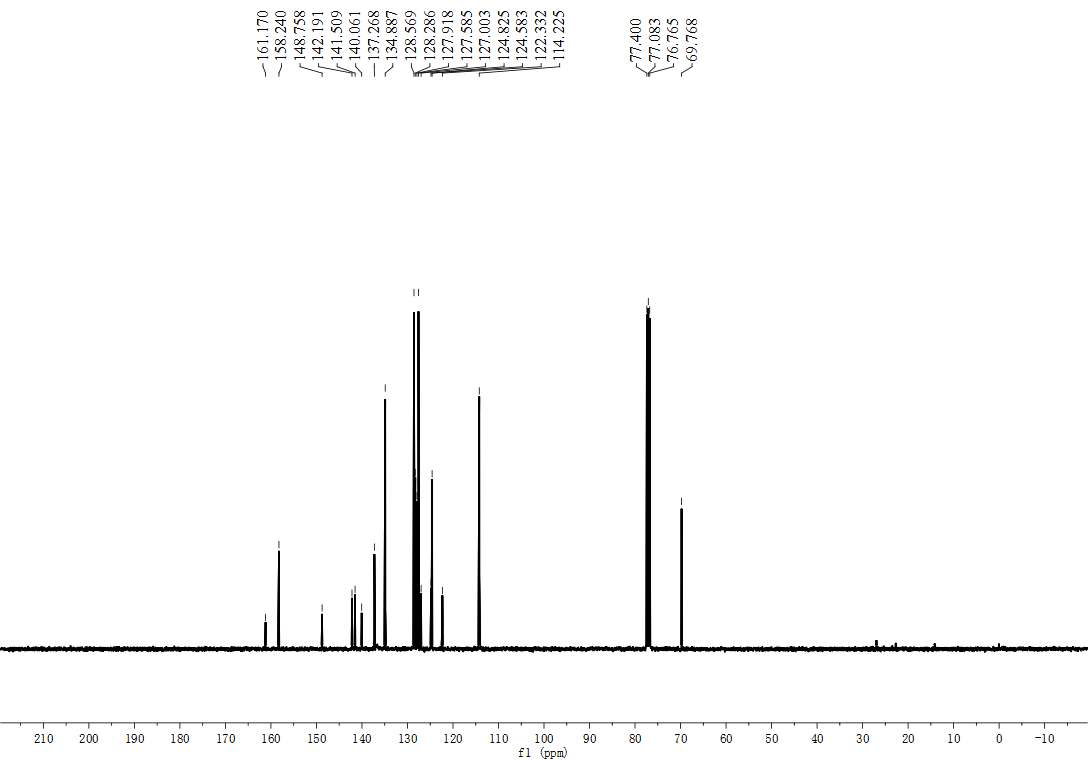


1. **Fluorescence decay profiles**

1. **Seven samples: solid and liquid quantum yields**


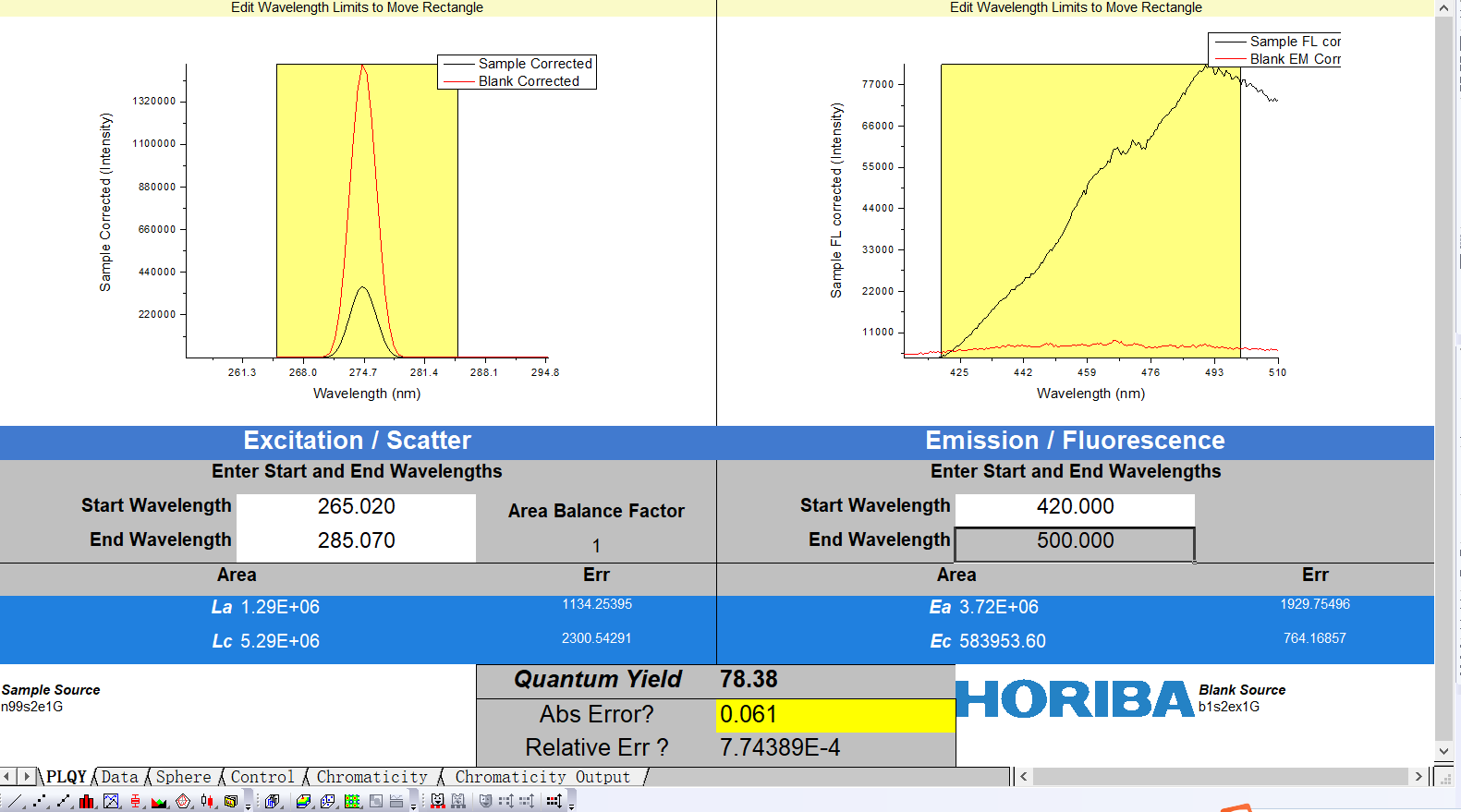


**3a solid quantum yields was 78.38 %.**


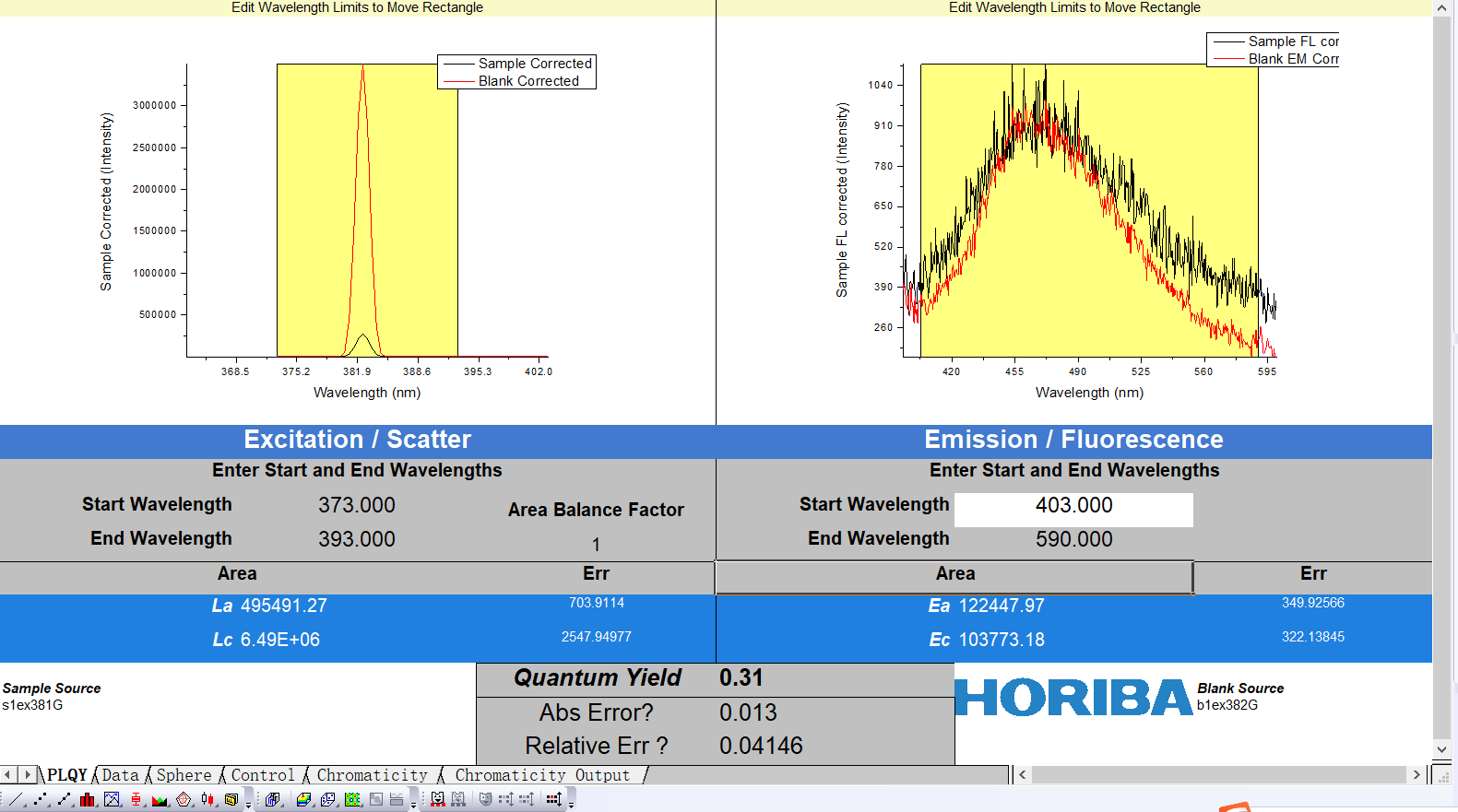


**3a in DMF quantum yields is 0.31 %.**


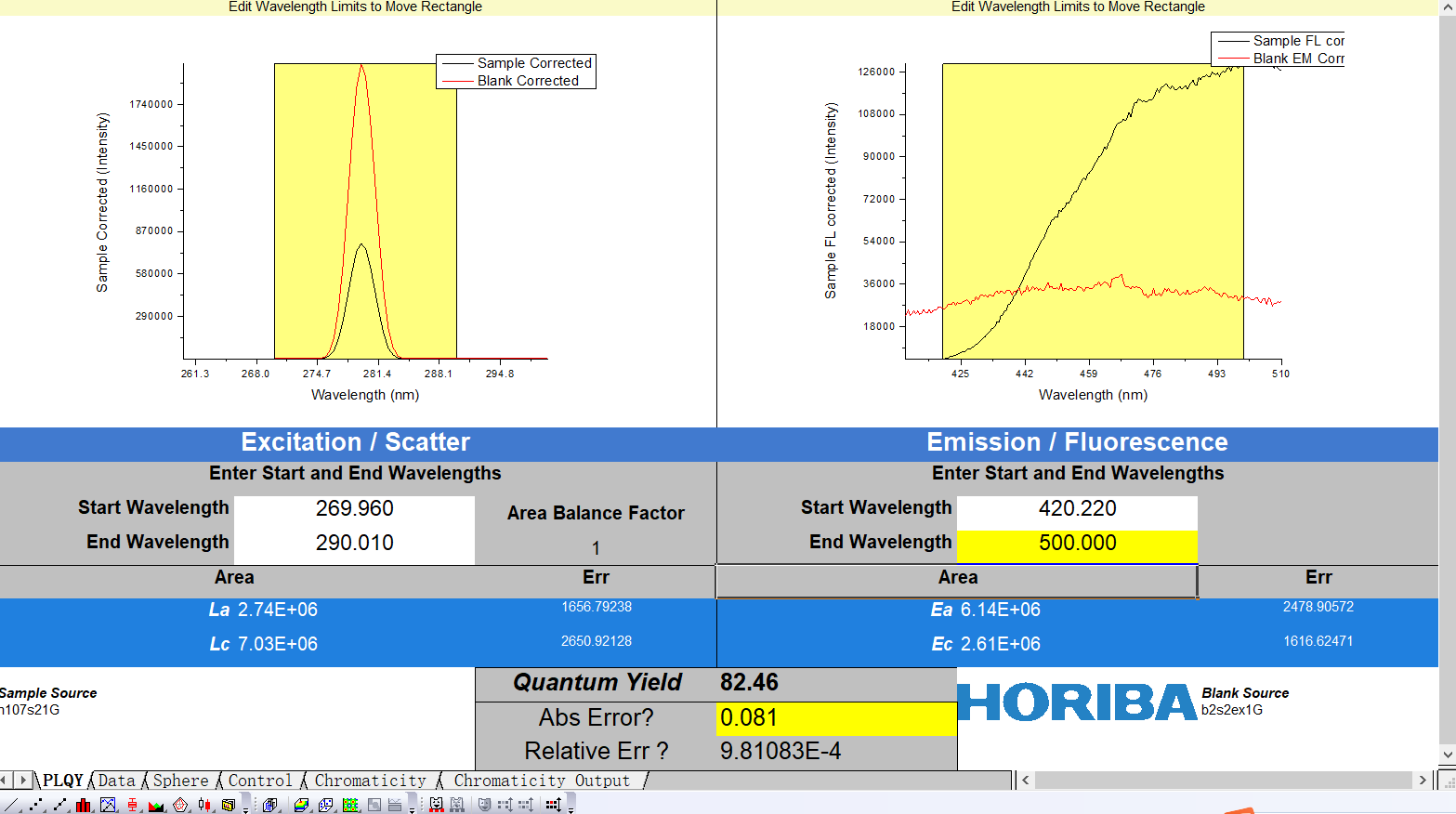


**3d solid quantum yields was 82.46 %.**


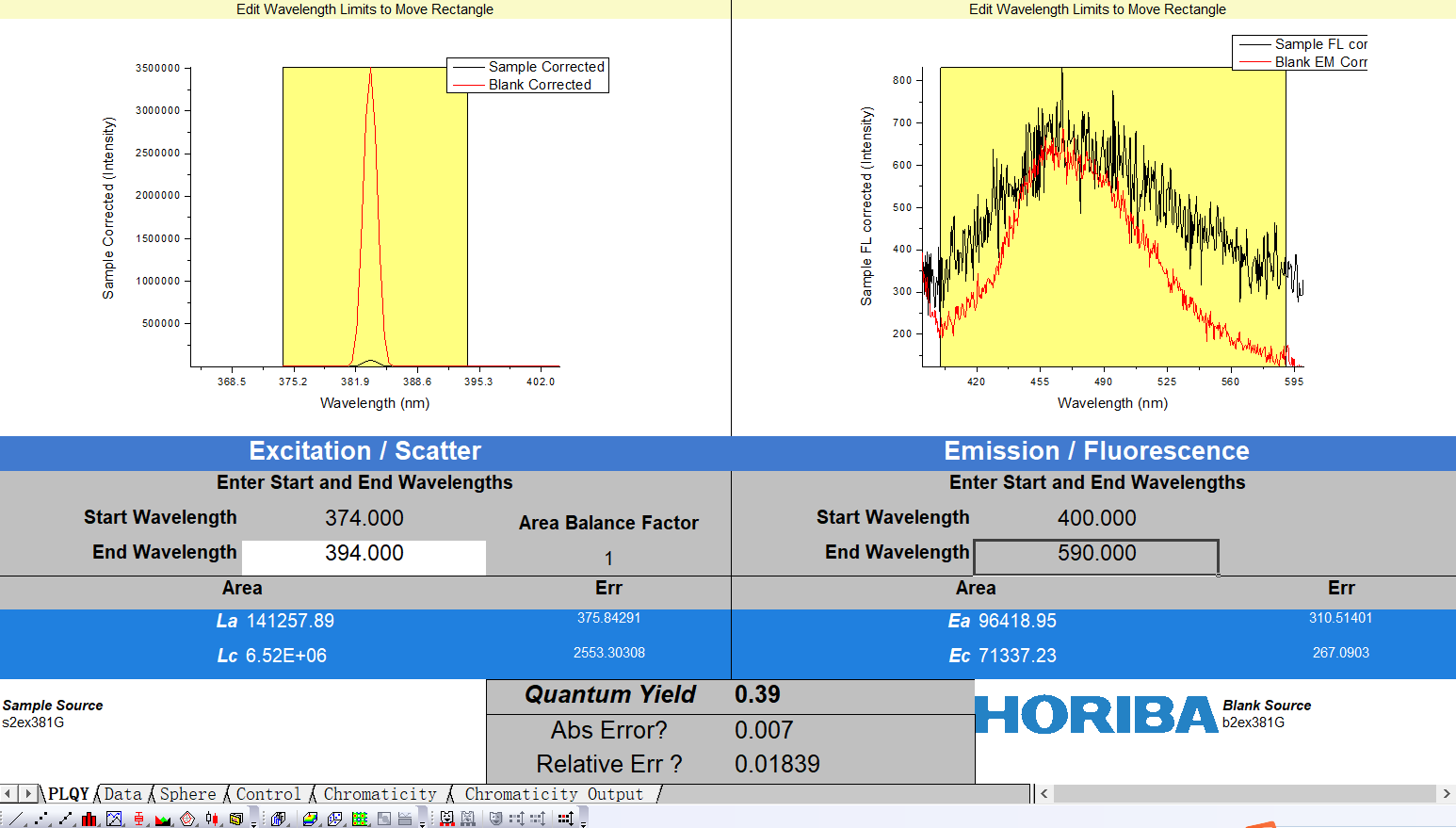


**3d in DMF quantum yields is 0.39 %.**


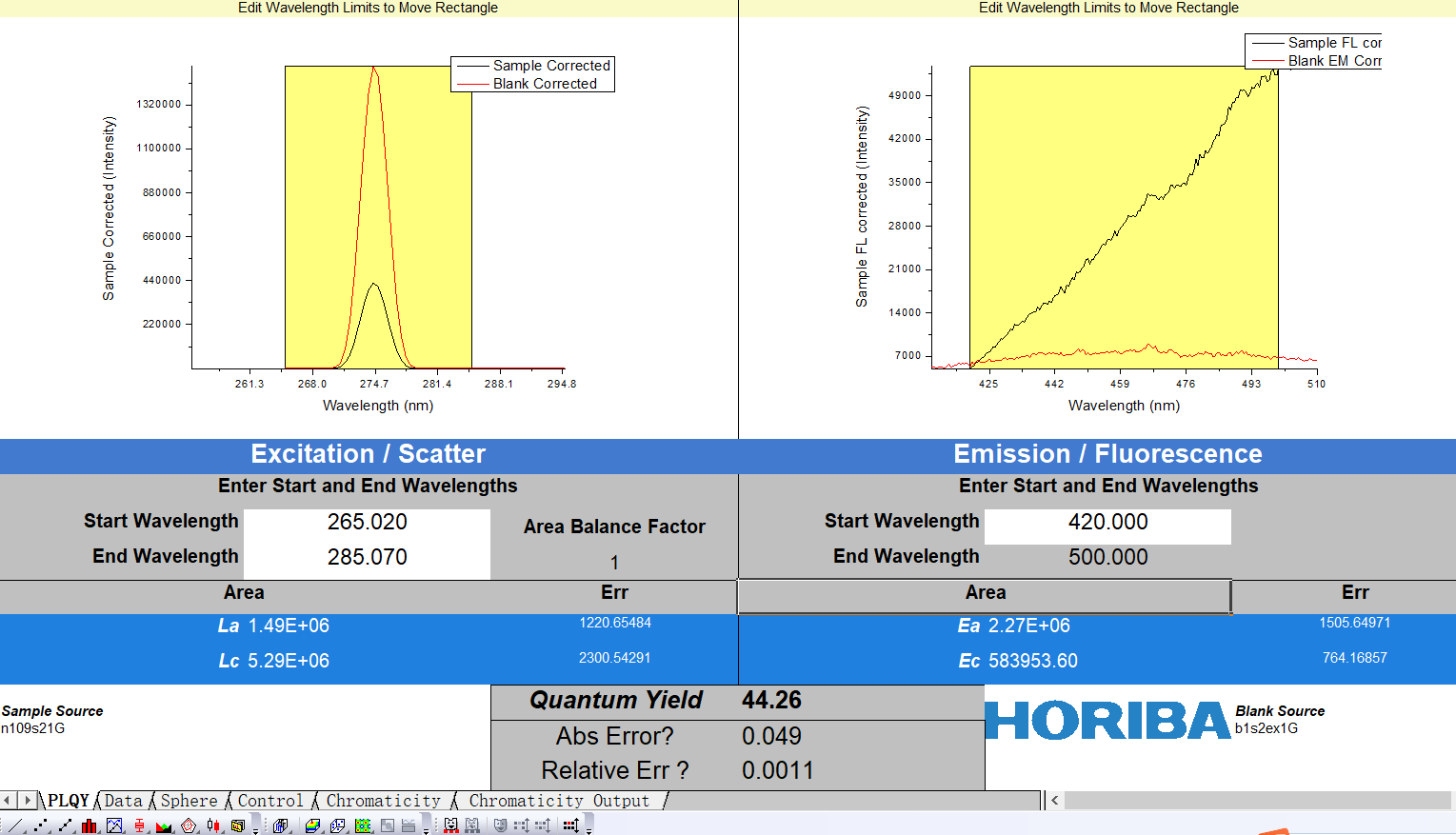


**3f solid quantum yields was 44.26 %.**


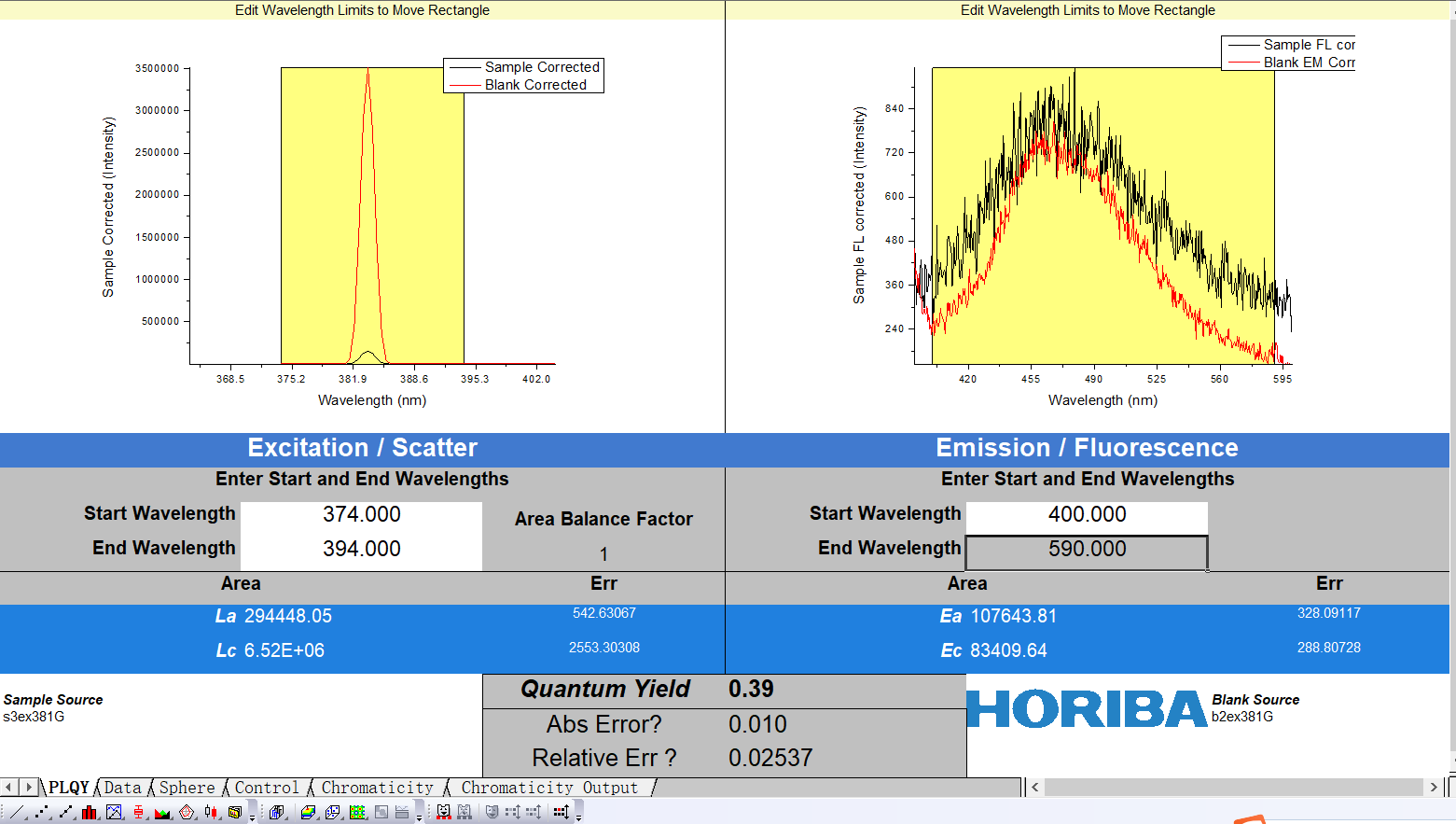


**3f in DMF quantum yields is 0.39 %.**


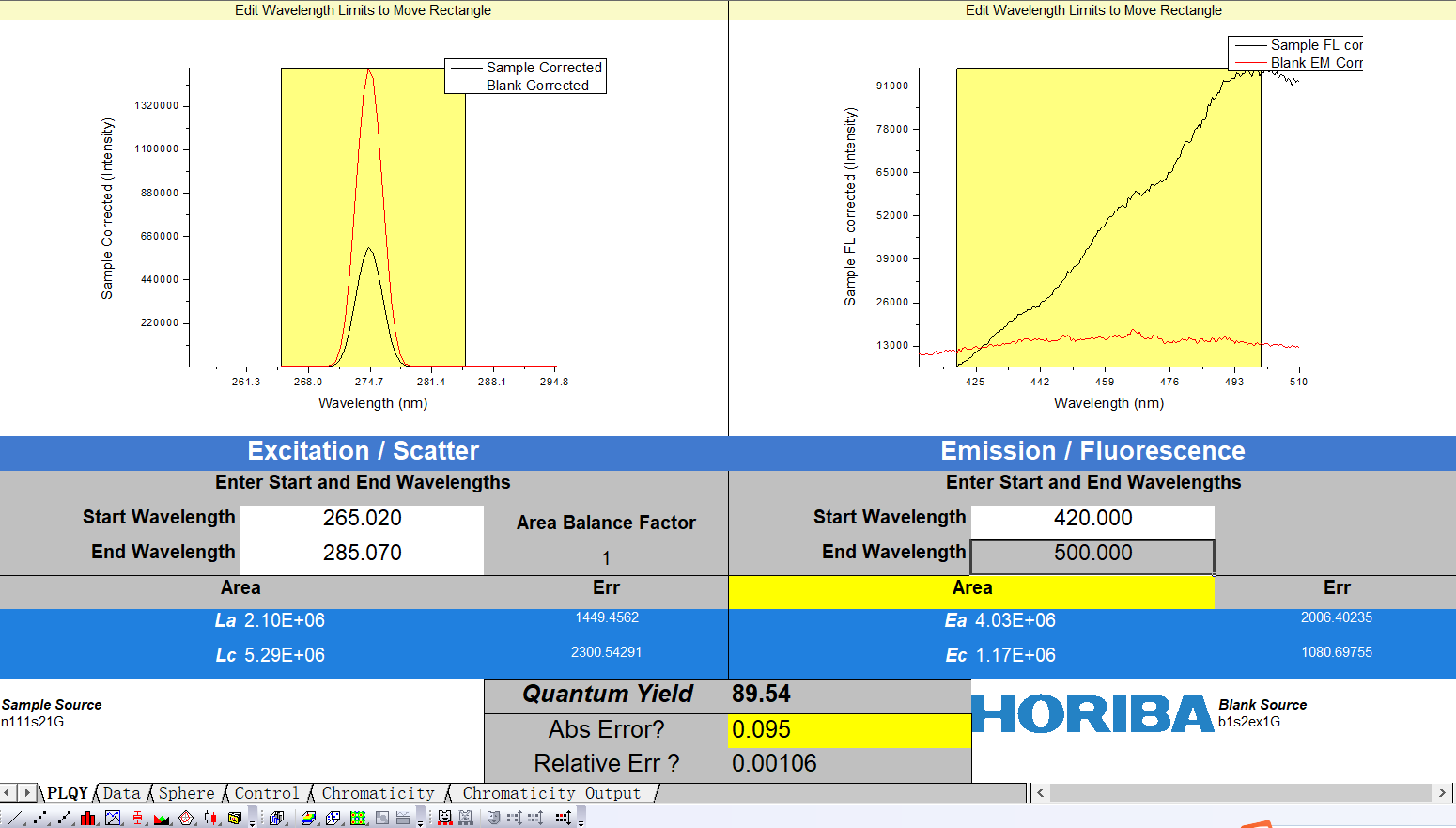


**3g solid quantum yields was 89.54 %.**


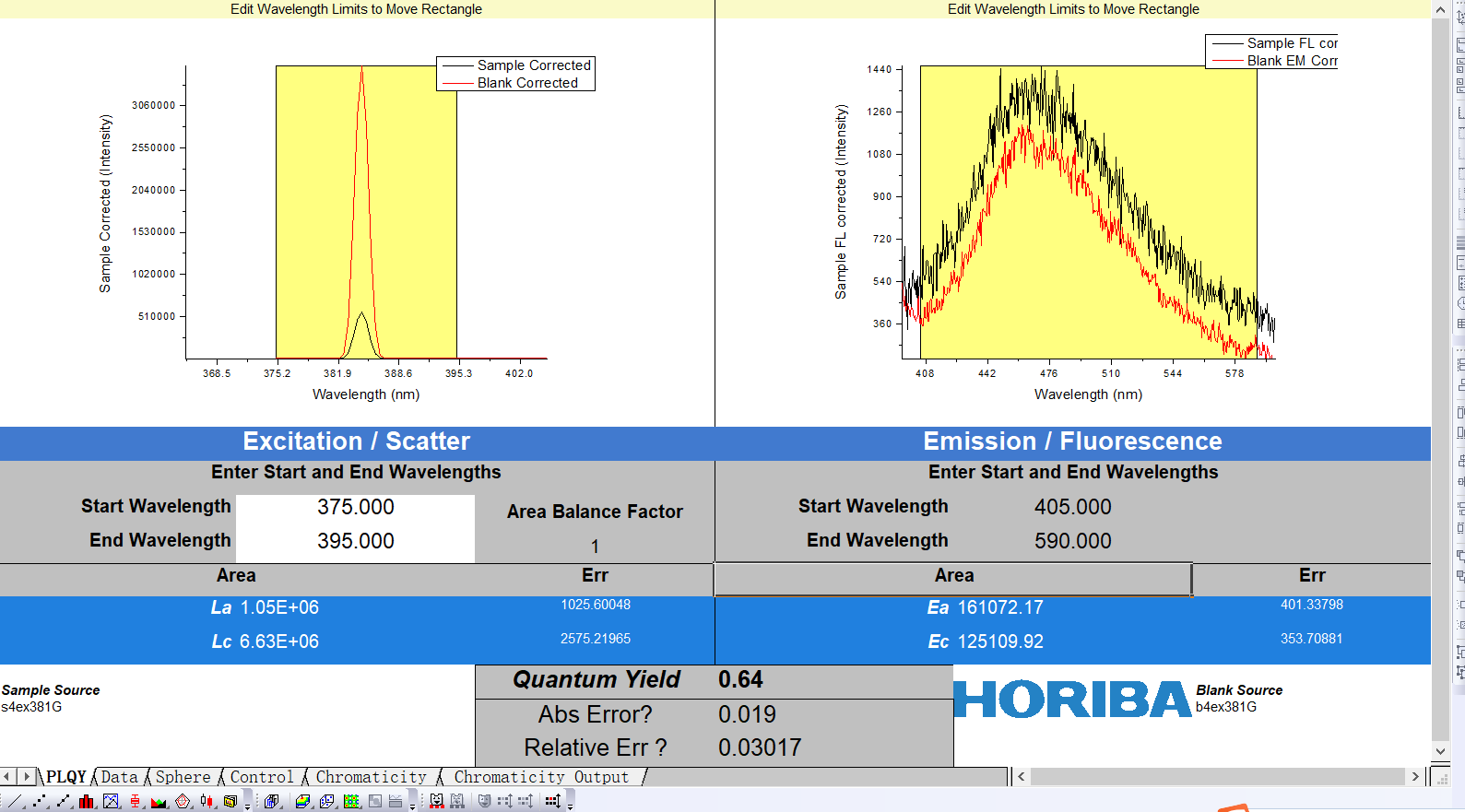


**3g in DMF quantum yields is 0.64 %.**


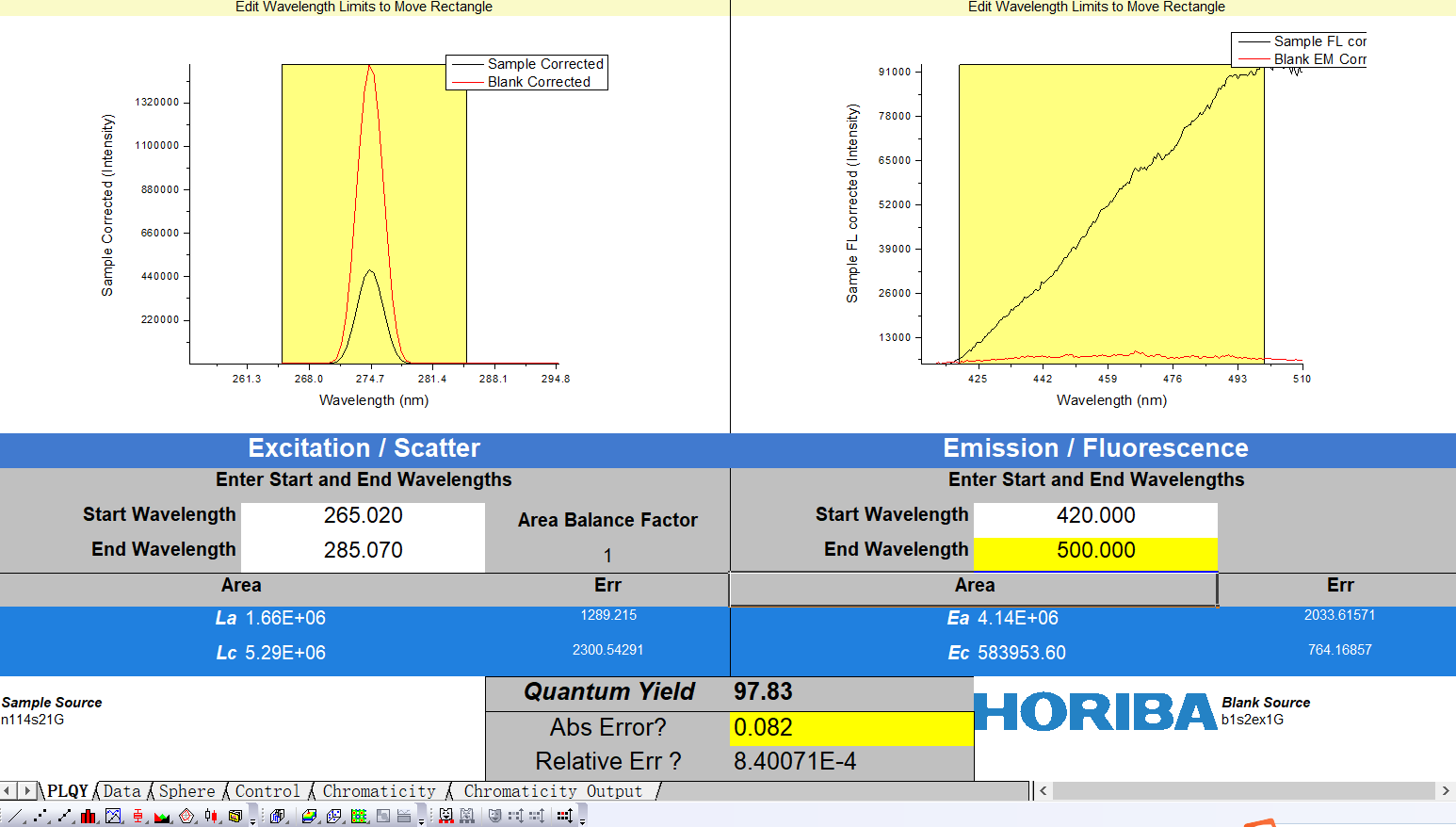


**3h solid quantum yields was 97.83 %.**


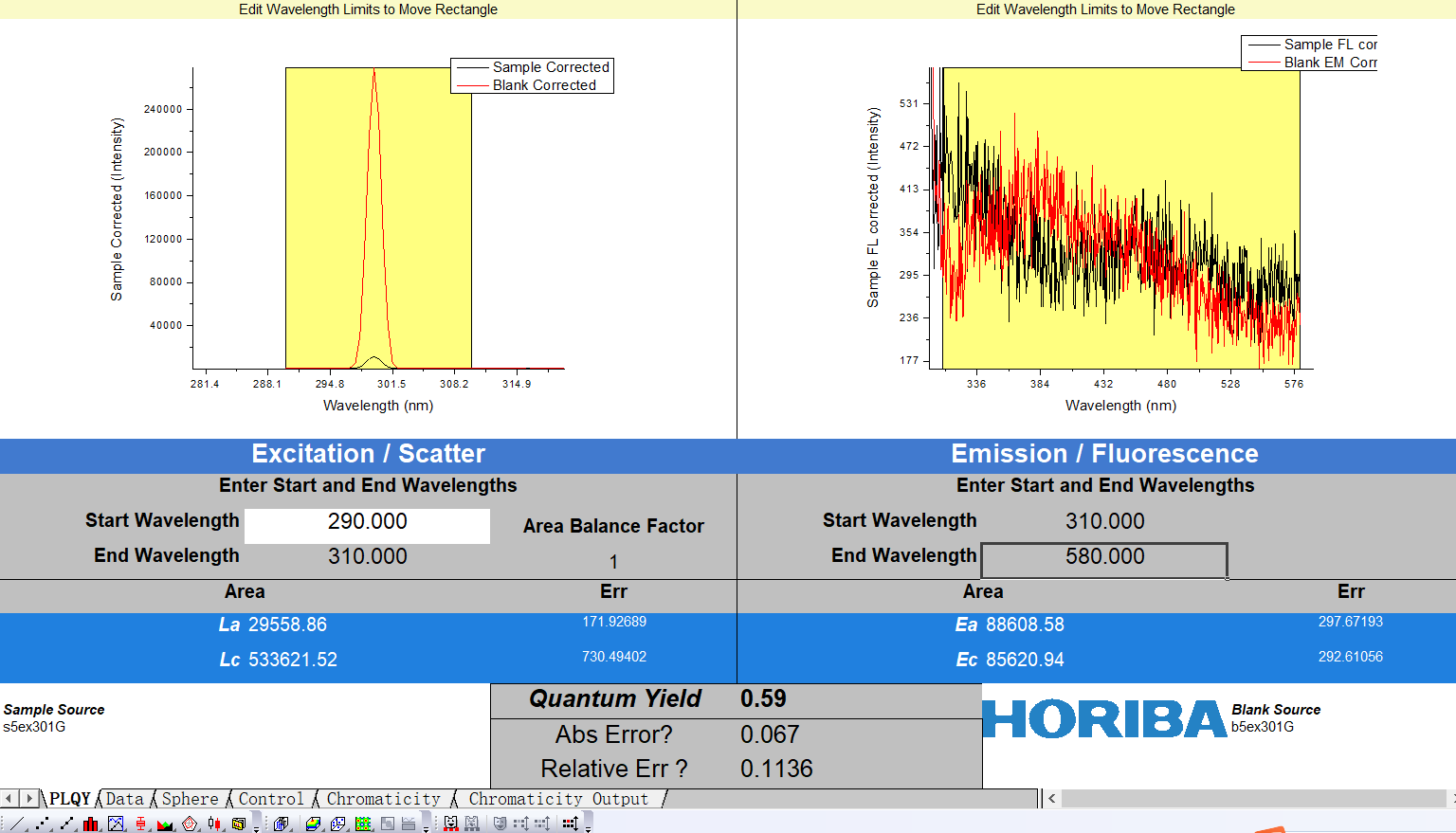


**3h in DMF quantum yields is 0.59 %.**


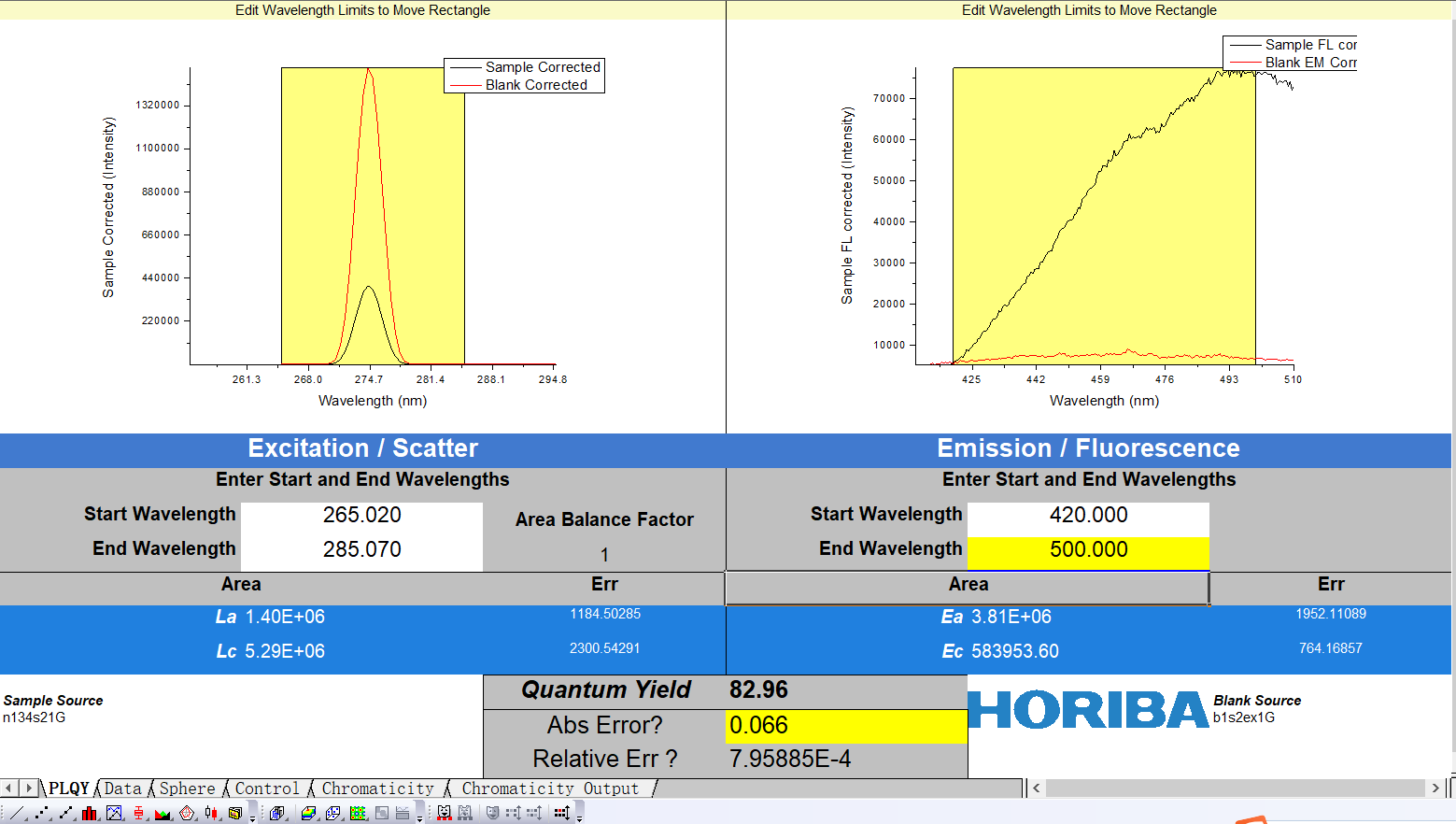


**3m solid quantum yields was 82.96 %.**


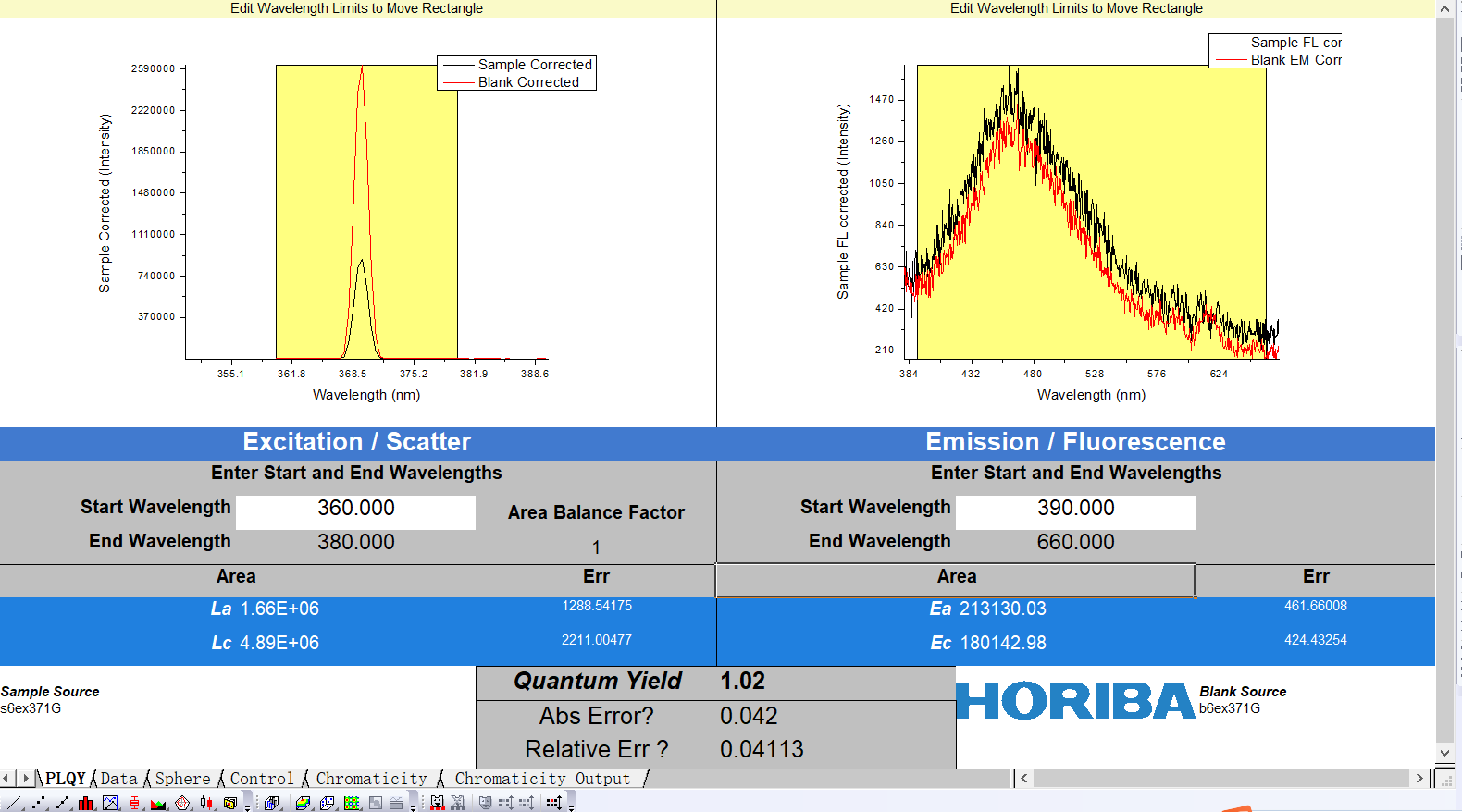


**3m in DMF quantum yields is 1.02 %.**


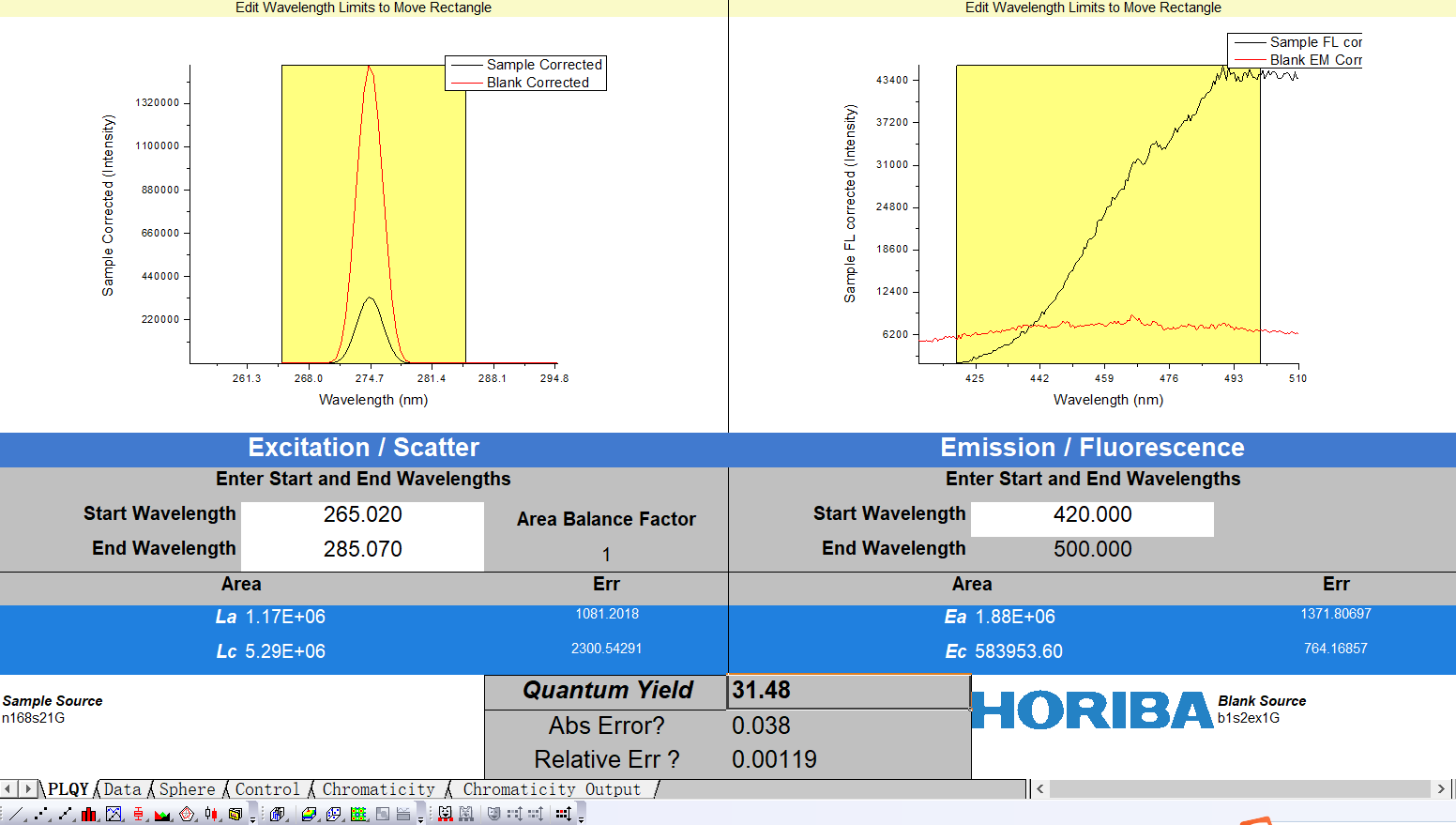


**3w solid quantum yields was 31.48 %.**


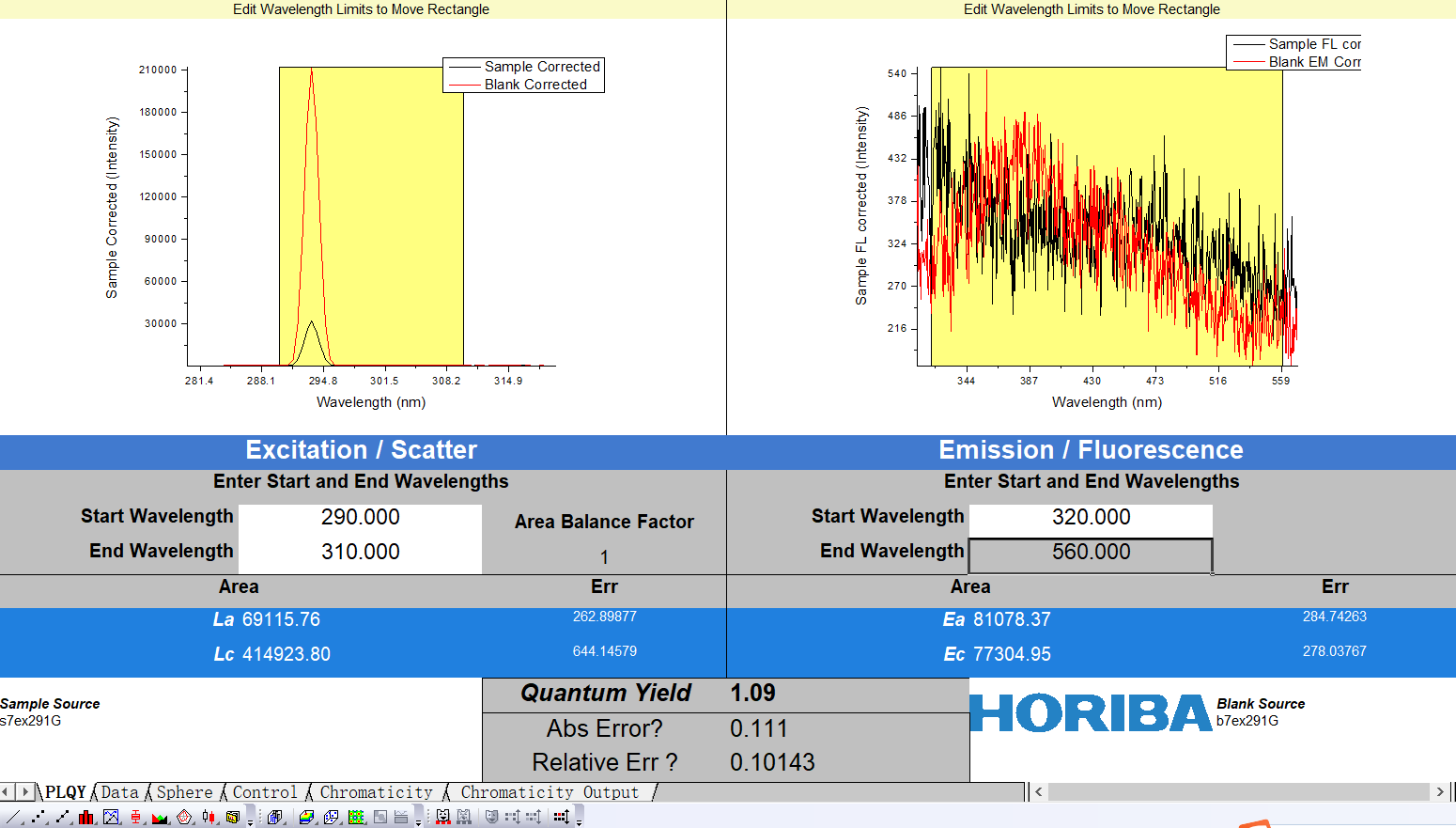


**3w in DMF quantum yields is 1.09 %.**

1. **Seven samples: solid and liquid fluorescence lifetimes**

**The fluorescence lifetime calculation formula is as follows：**

**Averaged fluorescence lifetimes =** $\boldsymbol{\tau}_{\boldsymbol{1}}\left( \boldsymbol{ns} \right)\boldsymbol{\times}\boldsymbol{B}_{\boldsymbol{1}}\boldsymbol{Rel(\%)+}\boldsymbol{\tau}_{\boldsymbol{2}}\left( \boldsymbol{ns} \right)\boldsymbol{\times}\boldsymbol{B}_{\boldsymbol{2}}\boldsymbol{Rel(\%)}$


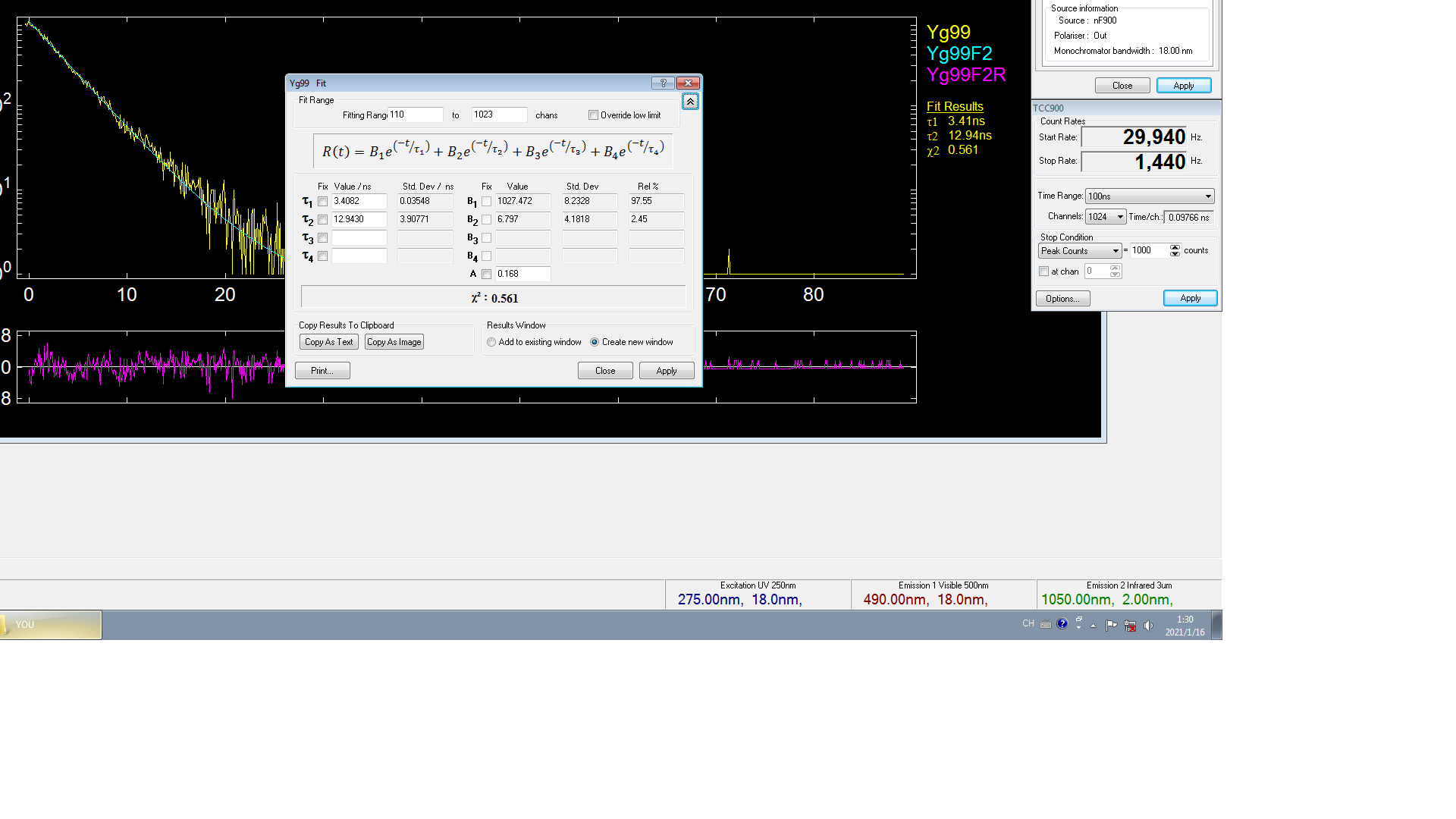
**3a The average fluorescence lifetime of a solid is 3.6418 ns.**

**
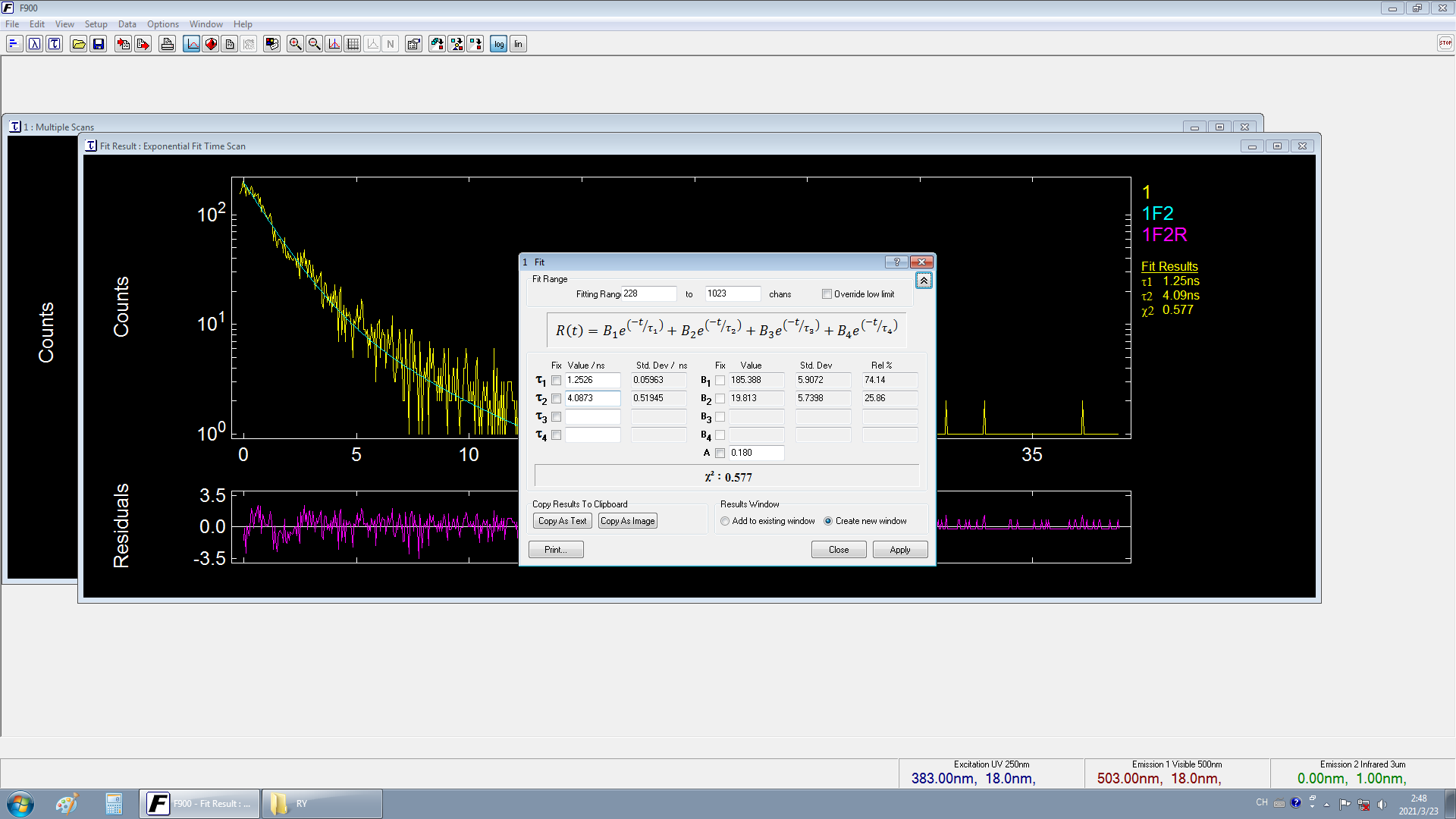
**

**3a The fluorescence lifetime in DMF is 1.9857 ns.**

**
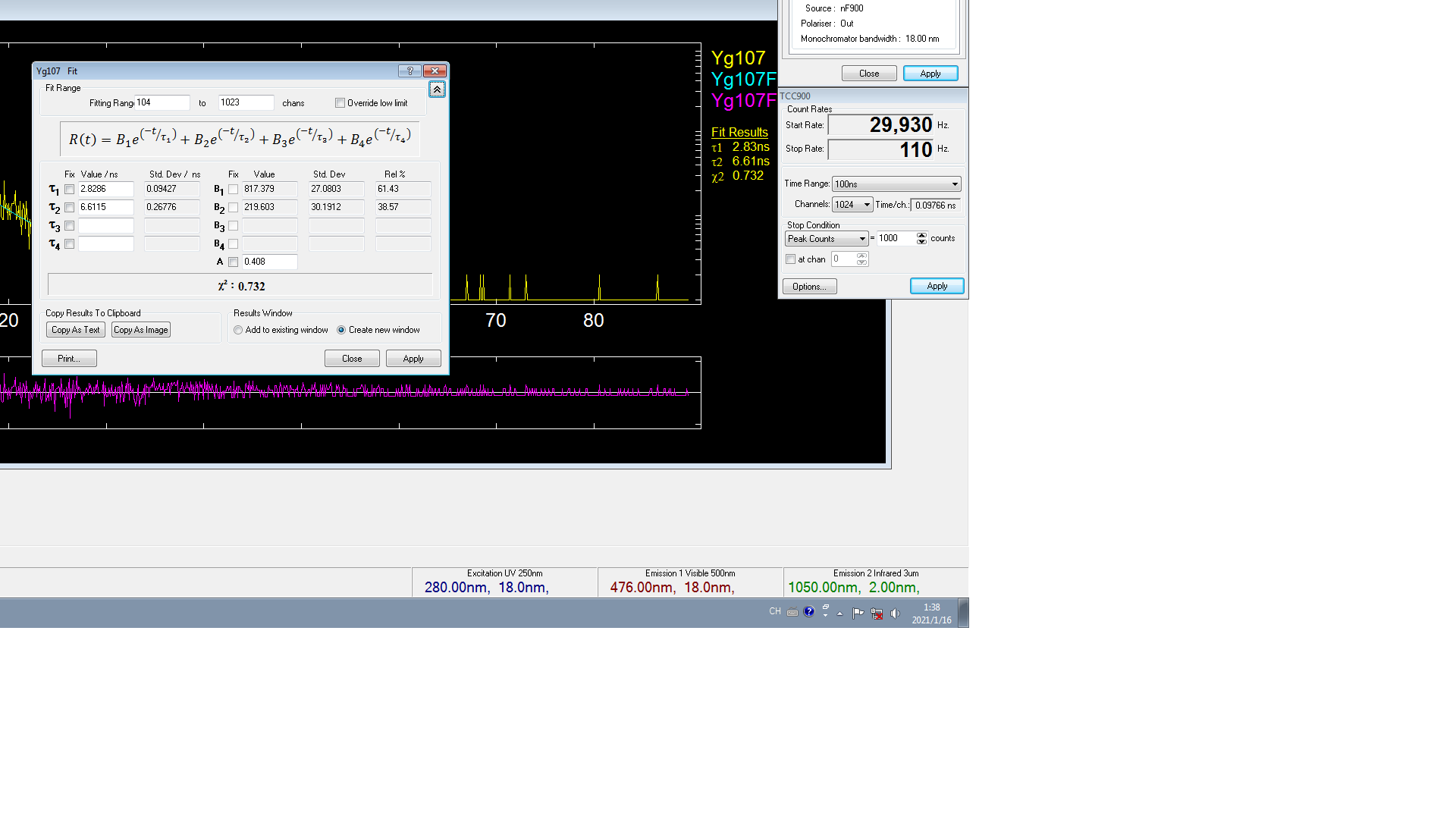
3d the average fluorescence lifetime of a solid is 4.2877 ns.**

**
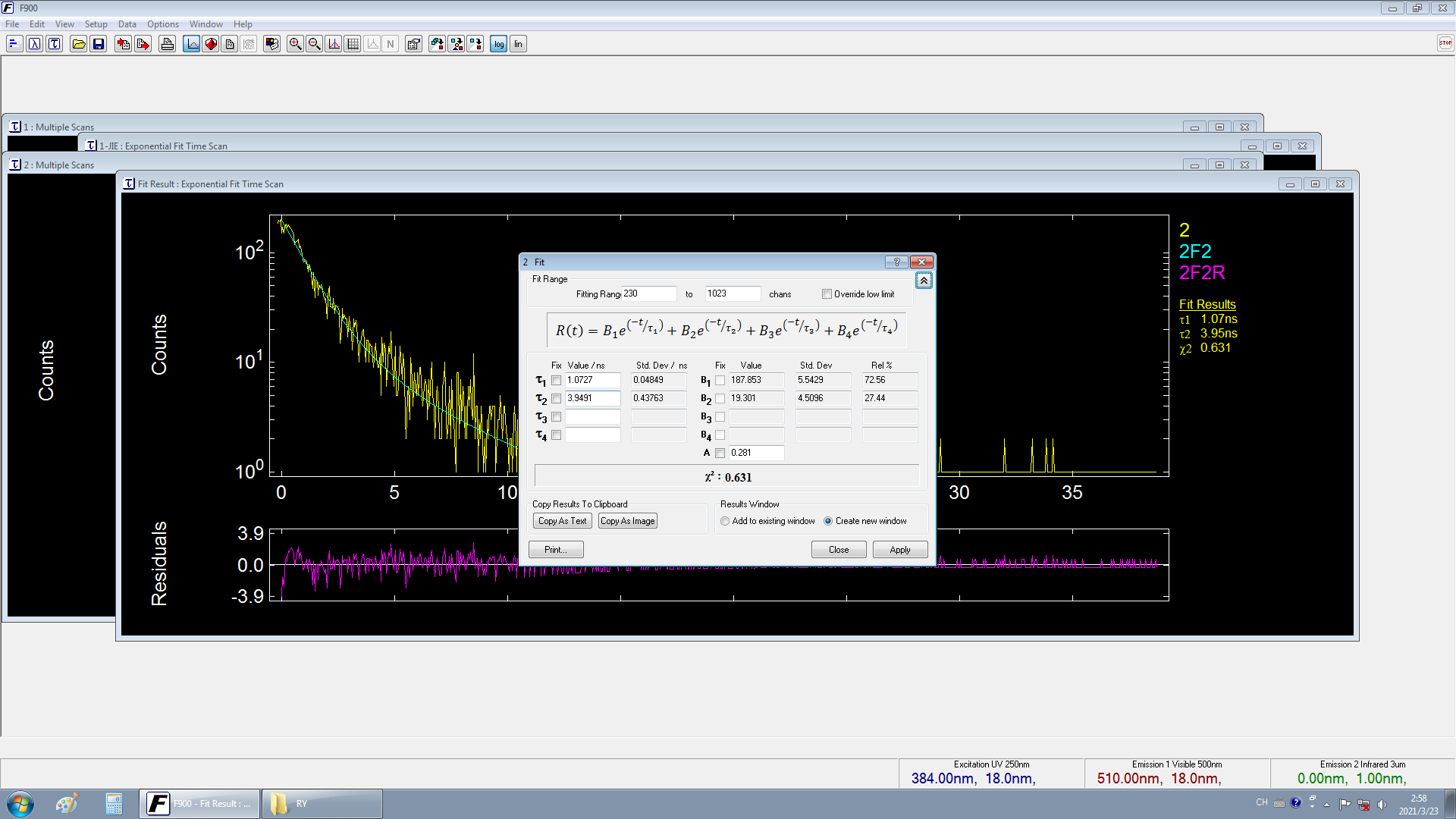
**

**3d** **the fluorescence lifetime in DMF is 1.862 ns.**

**
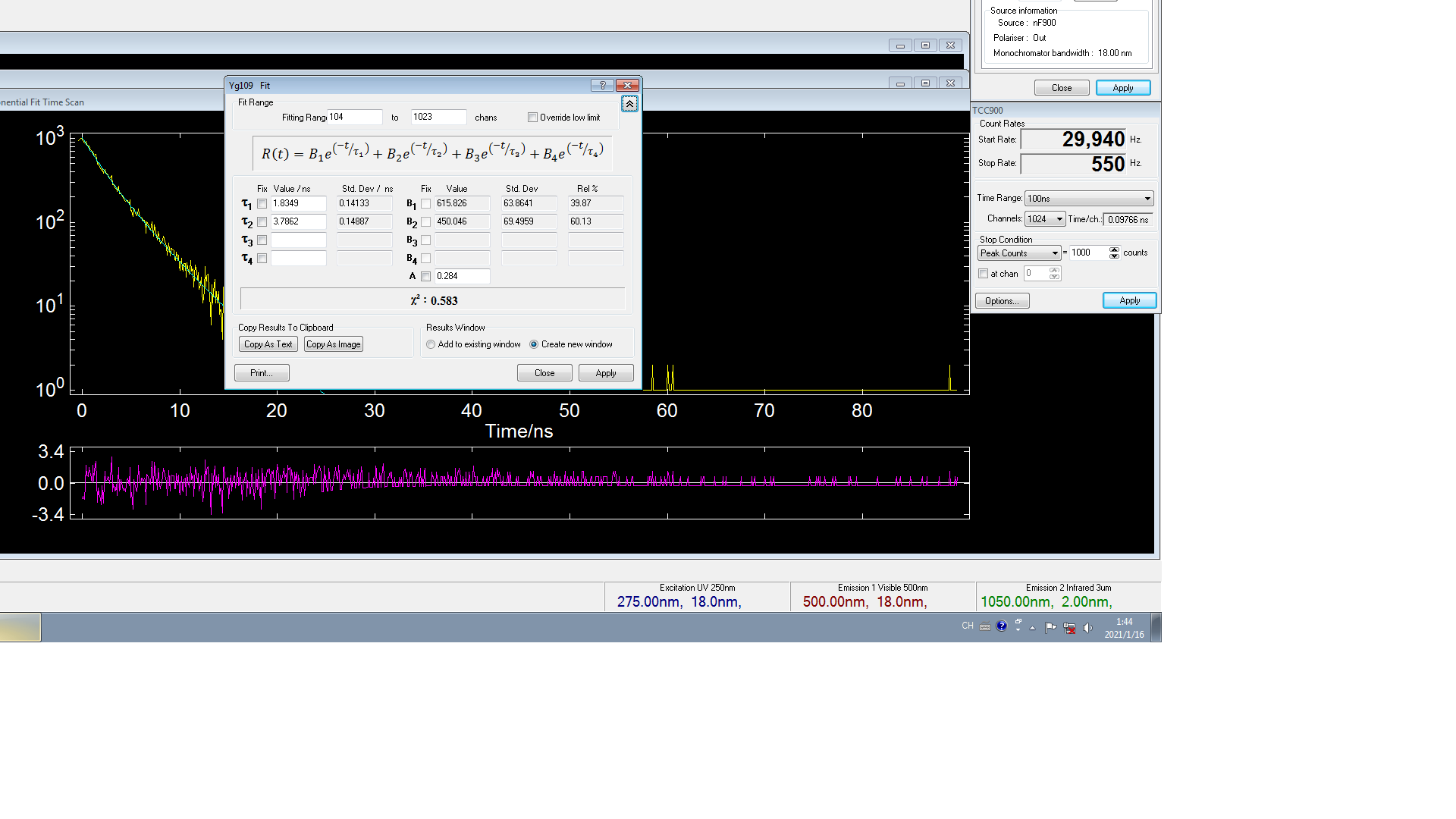
**

**3f the average fluorescence lifetime of a solid is 3.0082 ns.**

**
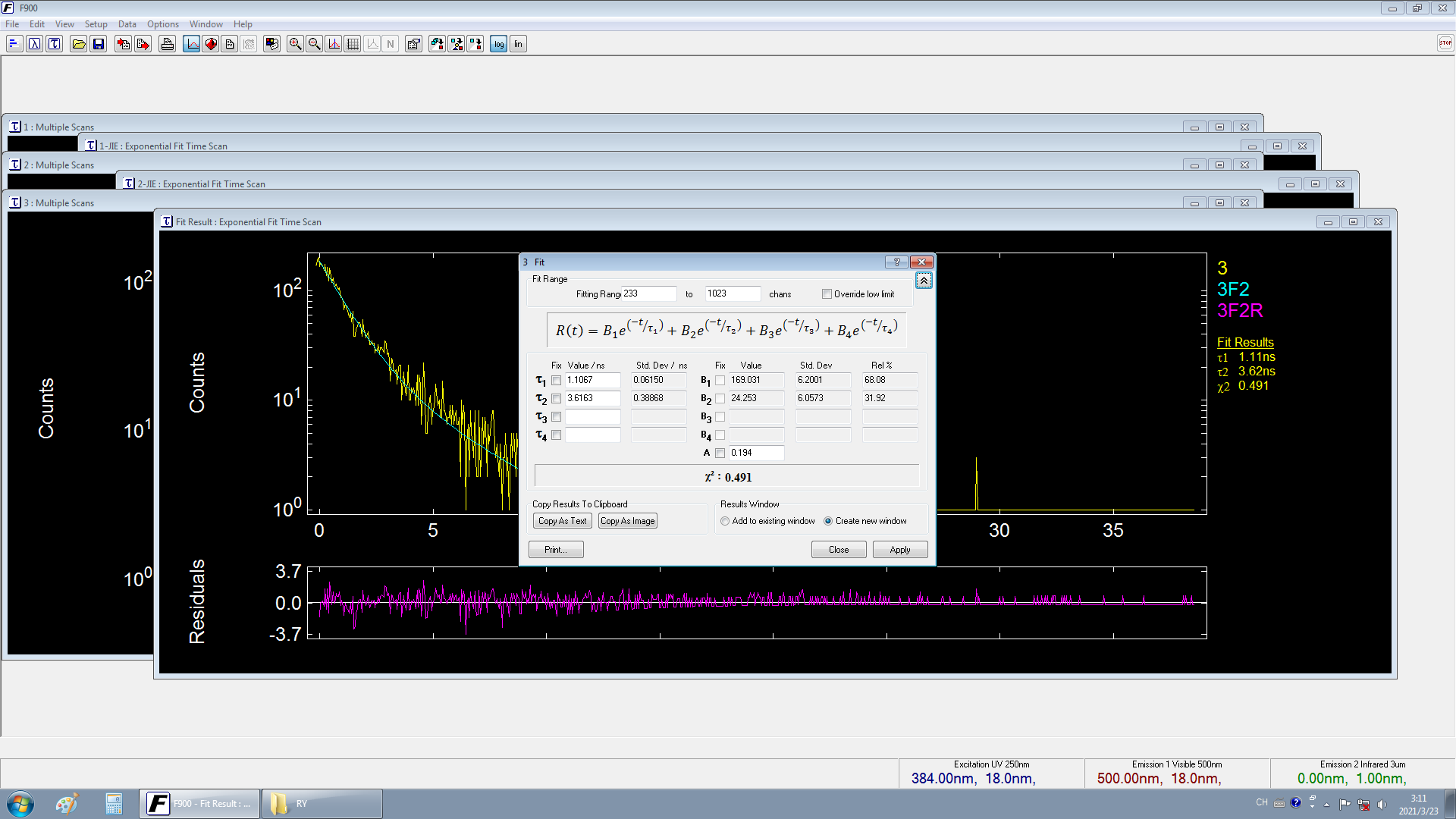
**

**3f** **the fluorescence lifetime in DMF is 1.9078 ns.**

**
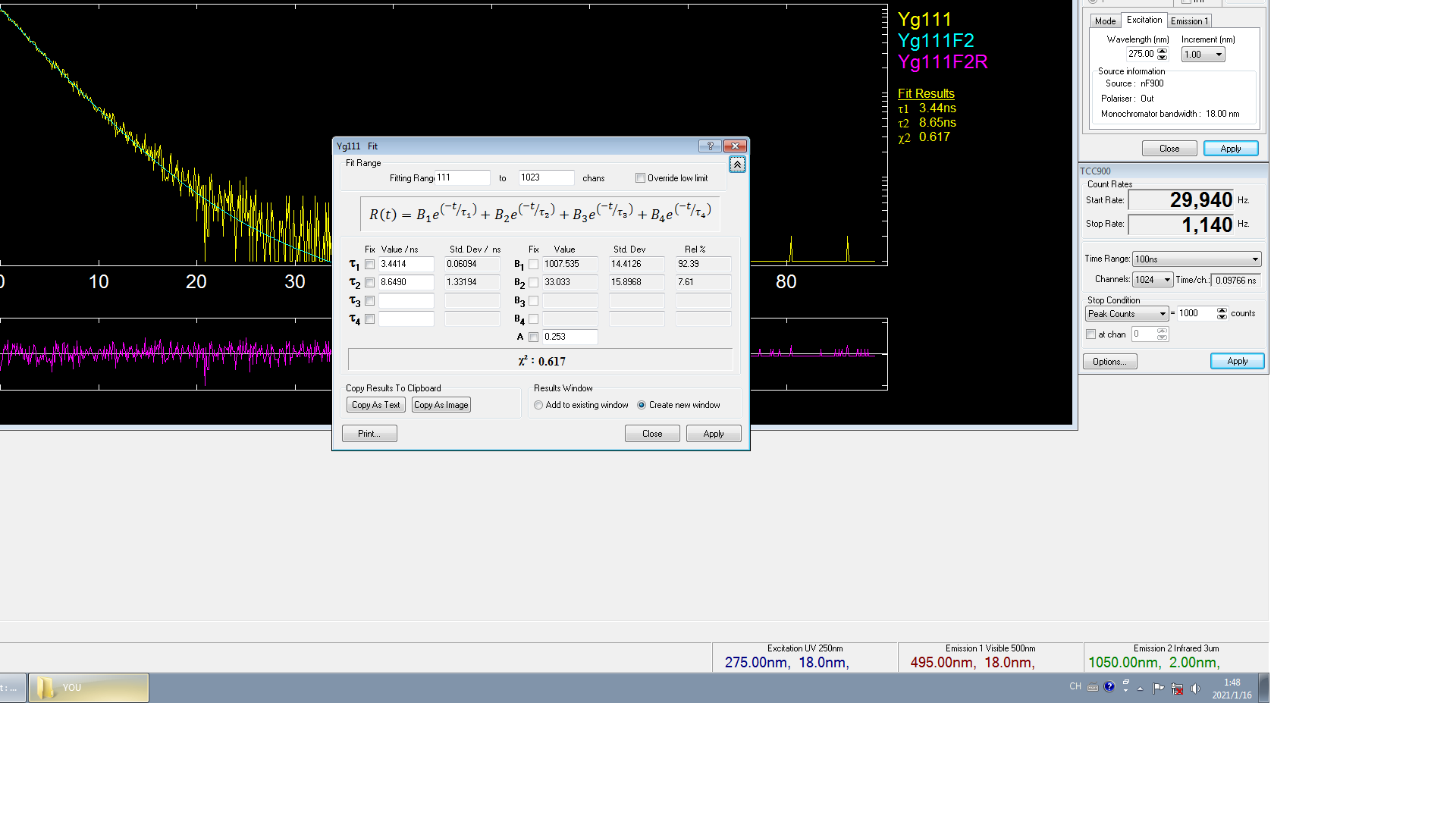
**

**3g the average fluorescence lifetime of a solid is 3.8377 ns.**

**
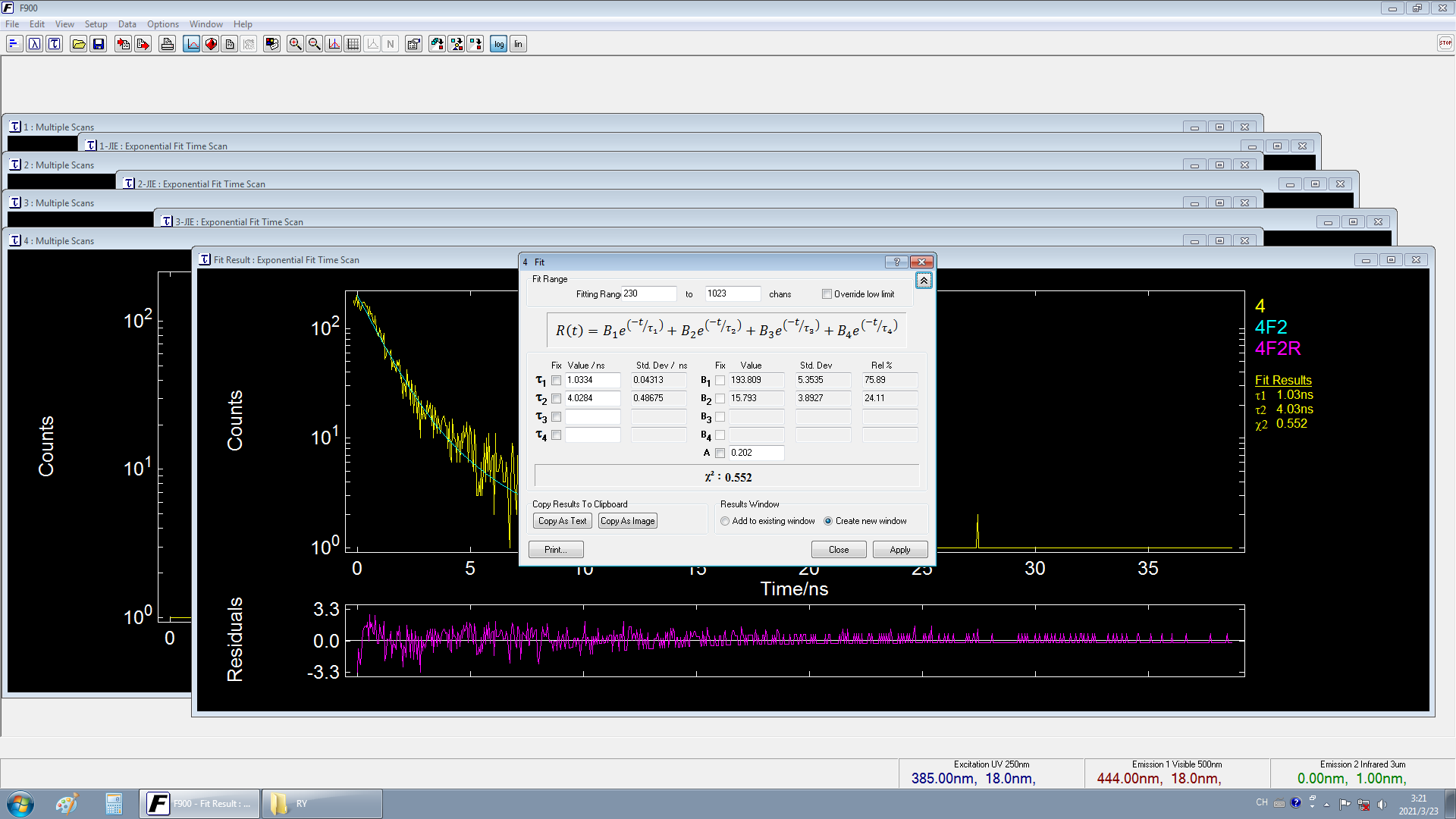
**

**3g** **the fluorescence lifetime in DMF is 1.7555 ns.**

**
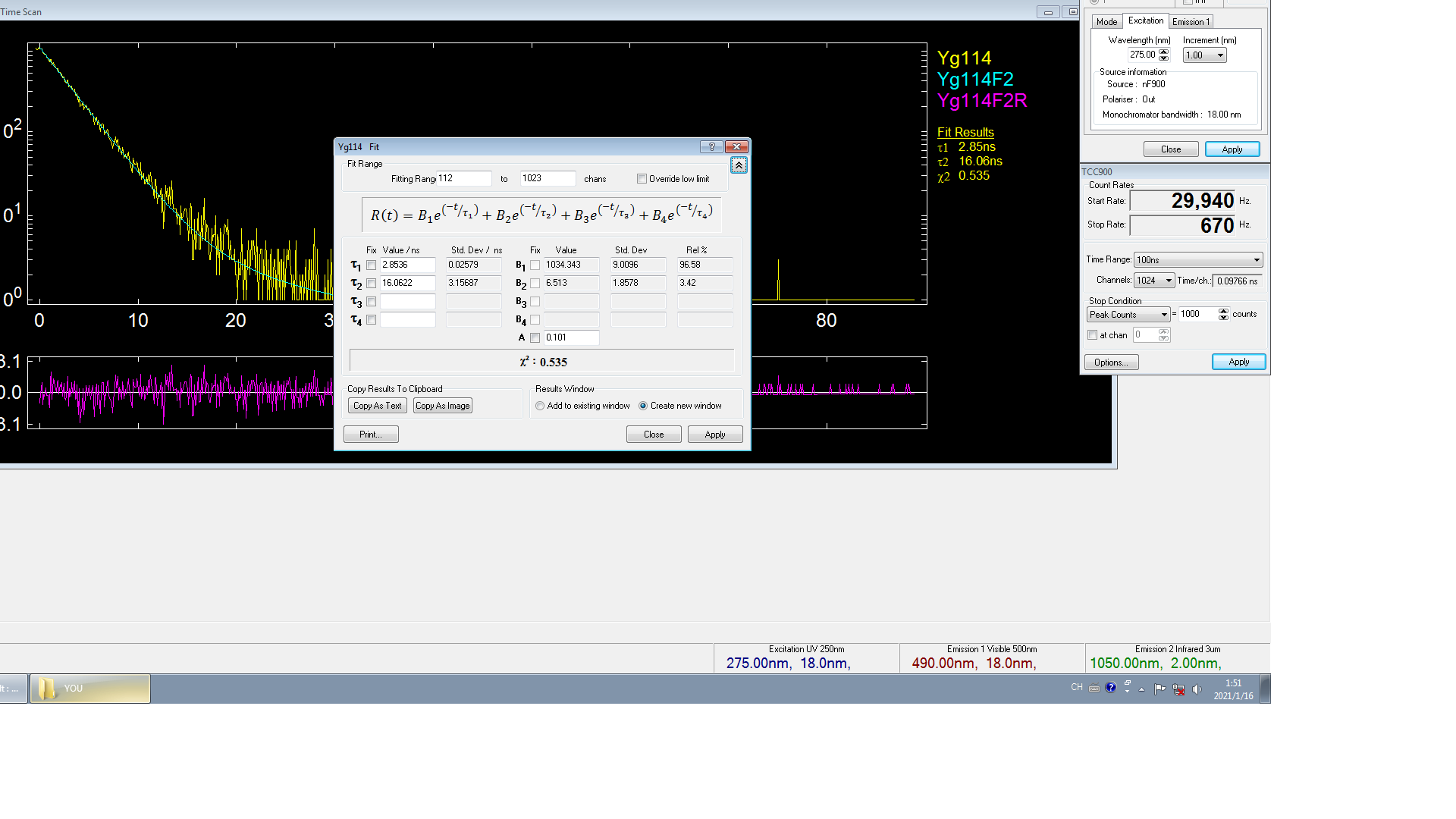
3h the average fluorescence lifetime of a solid is 3.3053 ns.**

**
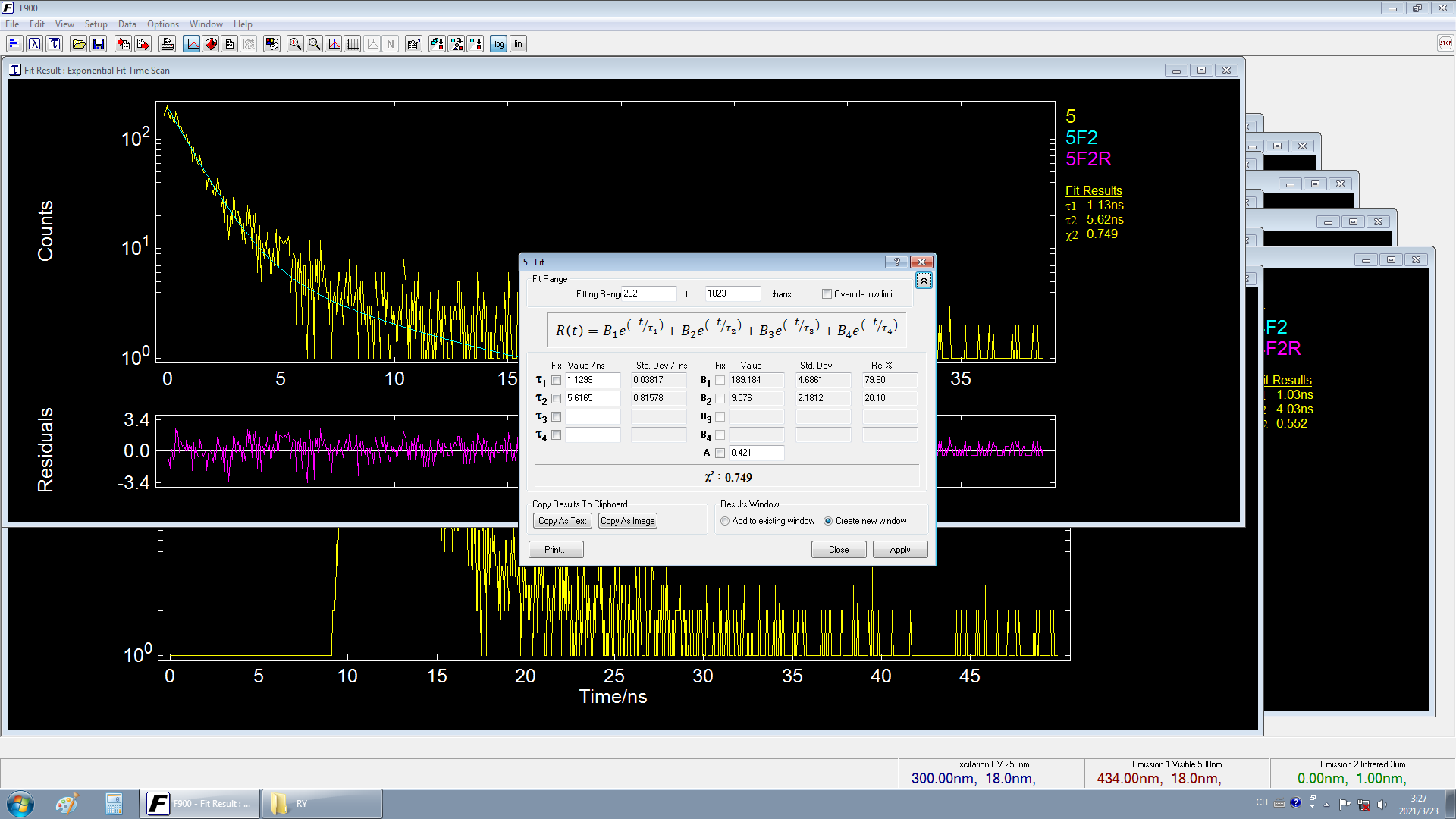
**

**3h** **the fluorescence lifetime in DMF is 2.0317 ns.**

**
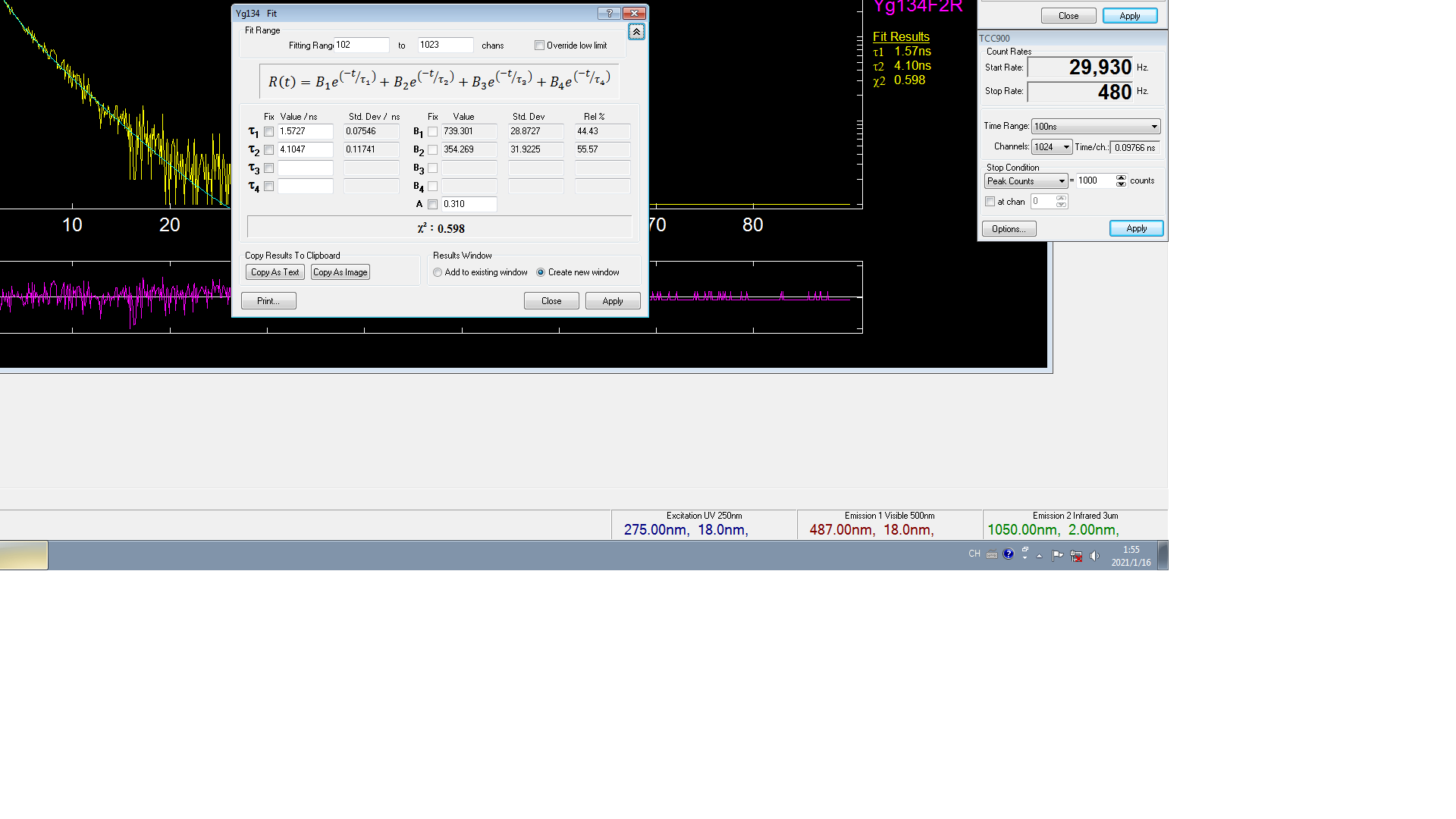
3m the average fluorescence lifetime of a solid is 2.9797 ns.**

**
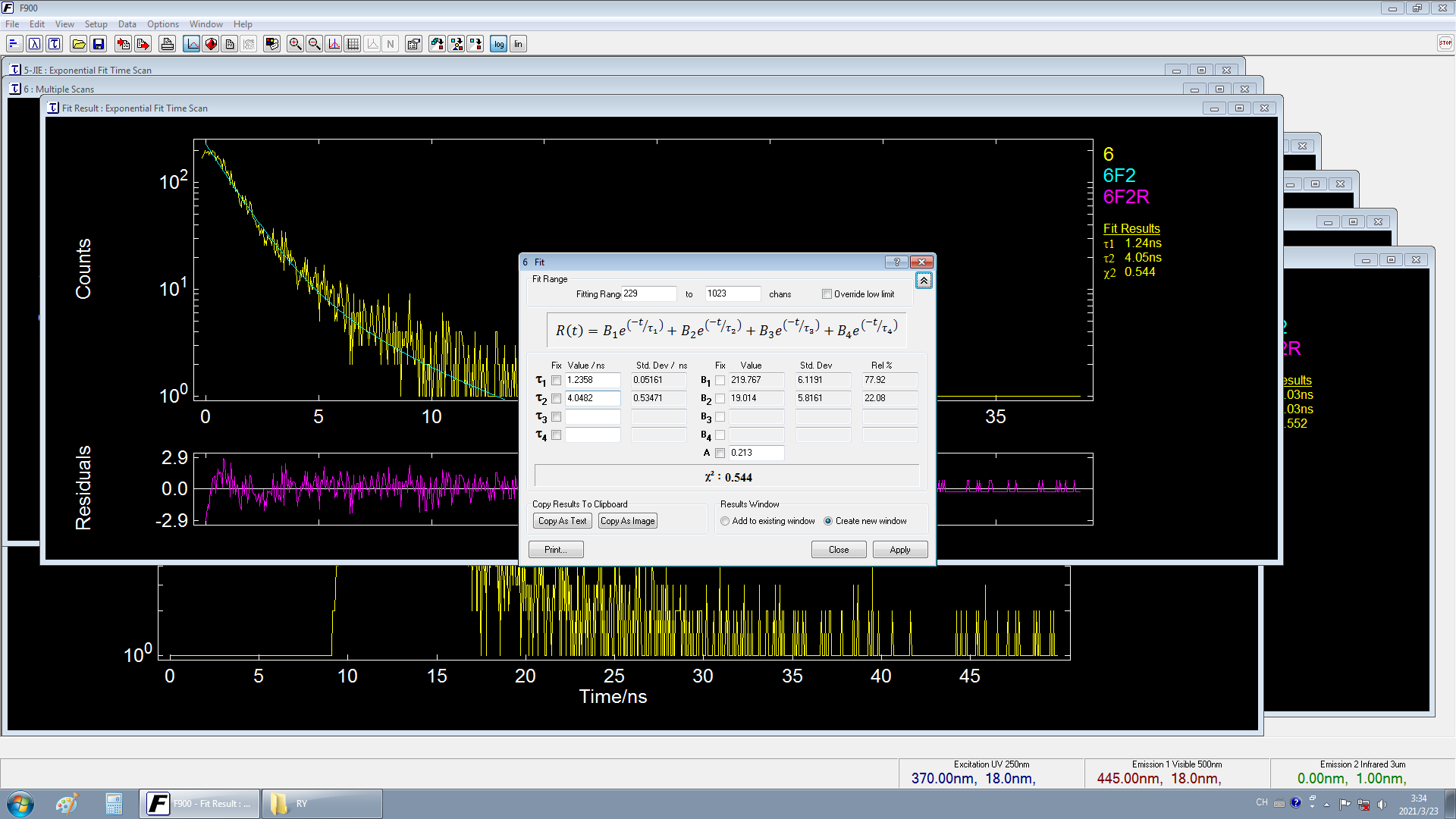
**

**3m** **the fluorescence lifetime in DMF is 1.8568 ns.**

**
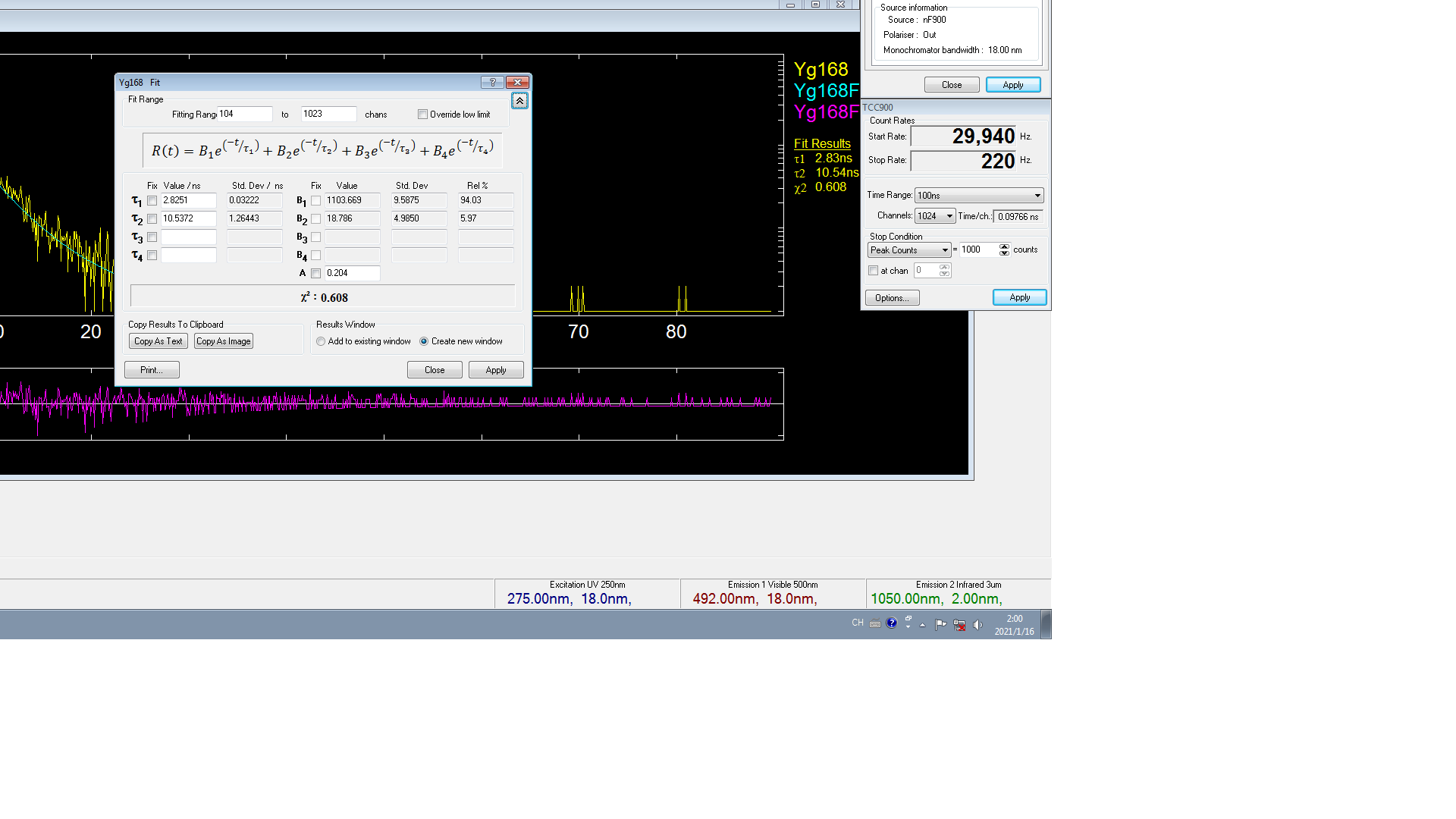
**

**3w the average fluorescence lifetime of a solid is 3.2855 ns.**

**
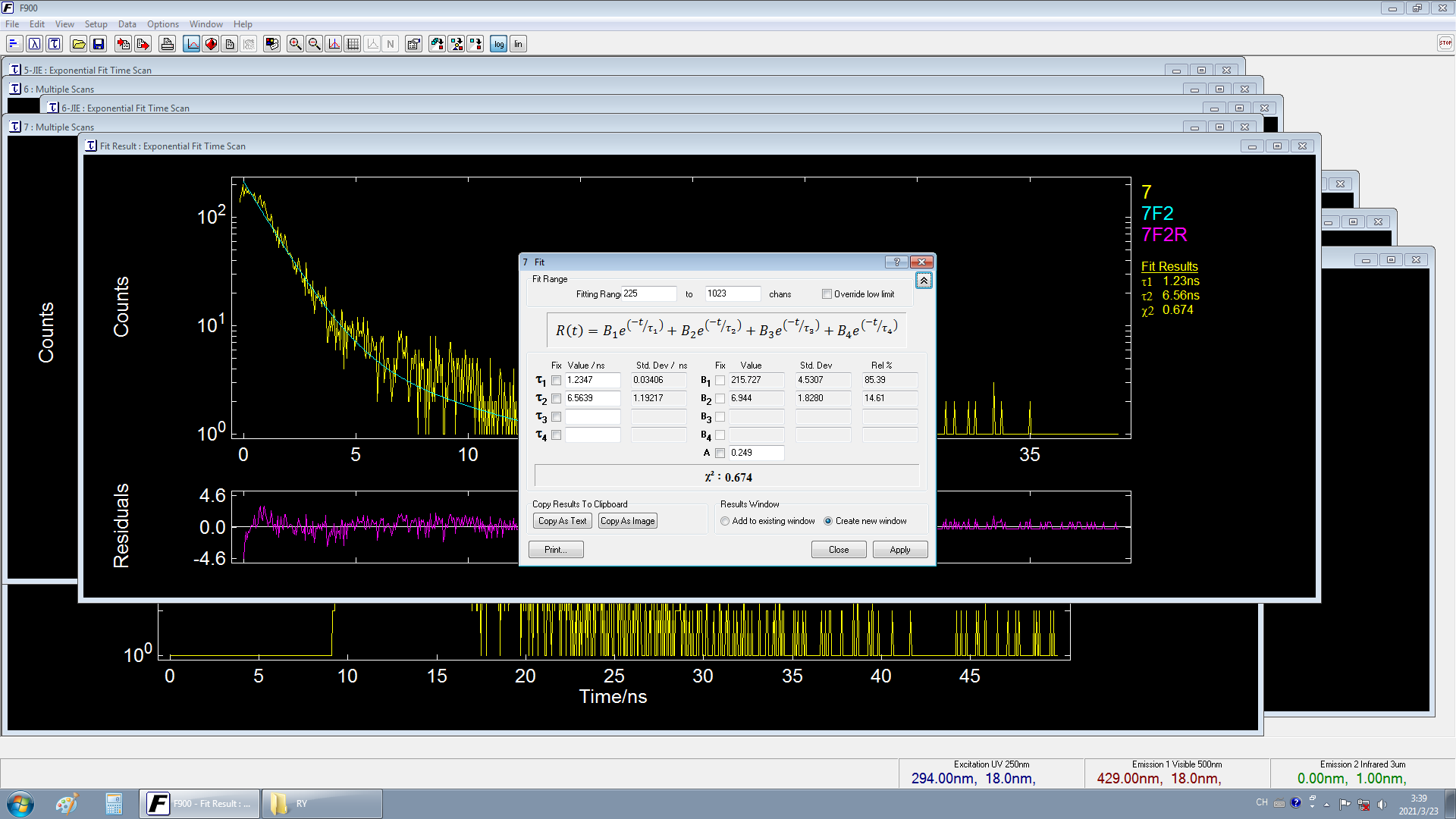
**

**3w** **the fluorescence lifetime in DMF is 2.0133 ns.**

1. **Cartesian coordinates**
2. **3a**

B -0.37549400 0.09206800 -0.16520100

C 1.96529500 -0.44739100 0.89971200

C 2.51306500 0.63403100 0.19897300

H 1.86635700 1.31727800 -0.33290000

C 3.88400600 0.84618000 0.18813900

H 4.28299500 1.69084800 -0.36086400

C 4.73733000 -0.01024700 0.87304900

H 5.80727300 0.15765000 0.86485800

C 4.19680200 -1.08406000 1.56892800

H 4.84665200 -1.76287000 2.10875300

C 2.82624900 -1.31085100 1.58958500

H 2.42382400 -2.14556900 2.14059900

C -0.08907400 -1.48799000 1.69964700

C -1.53525400 -1.47871600 1.29701100

C -2.55733900 -2.21248300 1.87202300

H -2.32715400 -2.85286100 2.71342800

C -3.82521400 -2.08267800 1.32445000

H -4.65921500 -2.63623500 1.73860800

C -4.02446100 -1.23745500 0.23194300

H -5.00195000 -1.12359200 -0.21632700

C -2.95119400 -0.53455200 -0.28476600

H -3.02726600 0.13742600 -1.13024000

C -0.63594600 1.65006500 0.17634300

C -1.35163000 2.48301200 -0.69452600

H -1.66233900 2.10537100 -1.66499100

C -1.66198000 3.79776800 -0.35875400

H -2.21512100 4.41838800 -1.05471400

C -1.25470700 4.31699000 0.86481200

H -1.48860100 5.34219400 1.12771100

C -0.53949400 3.51234900 1.74485500

H -0.21090000 3.90931600 2.69865900

C -0.24048600 2.19745400 1.40254000

H 0.32556800 1.58690700 2.09975800

C -0.04140500 -0.29737400 -1.69597100

C -0.20131800 -1.62255900 -2.12186500

H -0.56490600 -2.37125100 -1.42131300

C 0.10374500 -2.01967800 -3.41815300

H -0.02889500 -3.05370100 -3.71532400

C 0.58742400 -1.08828300 -4.33150400

H 0.83051400 -1.39102700 -5.34340700

C 0.76535500 0.23086100 -3.93347900

H 1.15223200 0.96123100 -4.63515400

C 0.45268100 0.61646700 -2.63254500

H 0.60297100 1.65149500 -2.34164700

N 0.56168100 -0.63854100 0.86494600

N -1.73959200 -0.66915600 0.25948000

O 0.30451400 -2.18407900 2.61460500
